# Without 1α-hydroxylation, the gene expression profile of 25(OH)D_3_ treatment overlaps deeply with that of 1,25(OH)_2_D_3_ in prostate cancer cells

**DOI:** 10.1038/s41598-018-27441-x

**Published:** 2018-06-13

**Authors:** Takao Susa, Masayoshi Iizuka, Hiroko Okinaga, Mimi Tamamori-Adachi, Tomoki Okazaki

**Affiliations:** 1 Department of Biochemistry, Teikyo University School of Medicine, 2-11-1 Kaga, Itabashi-ku, Tokyo, 173-8605 Japan; 2 Departments of Internal Medicine, Teikyo University School of Medicine, 2-11-1 Kaga, Itabashi-ku, Tokyo, 173-8605 Japan

## Abstract

Recently, the antiproliferative action of 1,25(OH)_2_D_3_ (1,25D3), an active metabolite of vitamin D_3_, in the management of prostate cancer has been argued rigorously. In this study, we found that at a physiological concentration, 25(OH)D_3_ (25D3), the precursor of 1,25D3 and an inactive form of vitamin D because of its much weaker binding activity to the vitamin D receptor (VDR) compared with 1,25D3, had a gene expression profile similar to that of 1,25D3 in prostate cancer LNCaP cells. By immunocytochemistry, western blotting, and *CYP27B1* and/or *VDR* knockdown by small interfering RNAs, we found that 10^−7^ M 25D3, which is within its uppermost physiological concentration in the bloodstream, induced VDR nuclear import and robustly activated its target genes in the virtual absence of *CYP27B1* expression. Comprehensive microarray analyses verified 25D3 bioactivity, and we found that 25D3 target gene profiles largely matched those of 1,25D3, while the presence a small subset of 25D3- or 1,25D3-specific target genes was not excluded. These results indicated that 25D3 shares bioactivity with 1,25D3 without conversion to the latter. Metallothionein 2A was identified as a 1,25D3-specific repressive target gene, which might be a prerequisite for 1,25D3, but not 25D3, to exert its anti-proliferative action in LNCaP cells.

## Introduction

The prohormone 25(OH)D_3_ (25D3) is considered a metabolic intermediate to elaborate an active form of vitamin D_3_, 1α,25(OH)_2_D_3_ (1,25D3)^1–4^. The synthesis of 1,25D3 is accomplished by a series of hydroxylase reactions^4,5^, which begins with the production of previtamin D_3_ from 7-dehydrocholesterol in association with ultraviolet radiation from sunlight in the skin, followed by spontaneous isomerization to vitamin D_3_ (also called cholecalciferol). Vitamin D_3_ is converted to 25D3 by C-25 hydroxylase (CYP2R1 and CYP27A1) in the liver. Subsequently, 25D3 is converted to 1,25D3 by 1α-hydroxylase (CYP27B1), mainly in the kidney. 1,25D3 robustly induces CYP24A1 mRNA/protein expression, which has C-24 hydroxylase activity for 25D3 and 1,25D3, resulting in the inactivation of both forms of vitamin D_3_, while it also represses *CYP27B1* expression^5–7^. As a result, a feedback mechanism maintains the serum level and activity of 1,25D3 within a narrow physiological range.

1,25D3 is generally regarded as a unique bioactive ligand among vitamin D_3_ metabolites for the vitamin D_3_ receptor (VDR), although a secondary bile acid, lithocholic acid, also activates the VDR as another selective agonist in different physiological settings^8,9^. The 1,25D3-VDR axis has multiple functions in calcium and phosphate metabolism, the mineralization of bone, and with additional roles such as cell differentiation, muscle strength, immunity, and anti-cancer action^10,11^. On the other hand, some reports argue that 25D3 itself possesses hormonal activity through direct activation of the VDR in cell and organ culture experiments. The expression of *Pth* mRNA was suppressed by 25D3 in bovine parathyroid cells treated with the general cytochrome P450 inhibitor clotrimazole to inhibit 1α-hydroxylase^12^. It is also reported that 25D3 induces *Cyp24a1* expression in mammary gland organ culture cells derived from *Cyp27b1* knockout mice^13^. Furthermore, 25D3 stimulates *Cyp24a1* expression in kidney, prostate, and skin cells from *Cyp27b1* knockout mice^14^.

Despite these reports, 25D3 has gained little attention as a direct ligand for the VDR because of several drawbacks. One is that the binding affinity of 25D3 to the VDR, which is several hundred-fold lower than that of 1,25D3, is thought to be too weak to evoke substantial gene regulation by the VDR^12,15,16^. However, its actual serum concentration is indeed high enough to exert the biological activity of 1,25D3 via its interaction with the VDR. Given that the serum concentration of 25D3 (25–200 nM)^17^ is 1000 times higher than that of 1,25D3 (50–150 pM)^18^, it might well overcome its low gene transcriptional activity due to its weaker binding potential to VDR compared with that of 1,25D3. Furthermore, *CYP27B1*, which is mainly expressed in the kidney, is also produced from several peripheral tissues such as the skin, lymph nodes, colon, pancreas, adrenal medulla, brain, placenta^19^, parathyroid^20^, ovary^21^, vascular endothelial cells^22^, mammary gland^23^, and prostate^24^, resulting in the local production of 1,25D3 in these tissues. Such versatile *CYP27B1* expression has often confused the interpretation of clinical studies or basic research using experimental animals or cultured cells in which 25D3 as well as 1,25D3 were utilised to examine their respective hormonal effects, at least in some cells, either concomitantly or independently.

Several epidemiological studies suggested that high serum vitamin D_3_ levels, estimated by measuring 25D3, play important roles in the prevention of various forms of cancer, including breast, colon, and prostate cancer^25–27^. Some *in vitro* studies using VDR-expressing prostate cancer LNCaP cells reported that EB1089^28^, a potent analogue of 1,25D3 as well as 1,25D3^29^ has inhibitory effects on the growth of cancer cells, implying that 1,25D3 might be effective as a therapeutic reagent for prostate cancer in some cases. However, the growth inhibitory action of 25D3 on prostate cancer is still controversial. In immortalised human prostate cell lines, such as PZ-HPV-7 cells, 10 nM of 25D3 was reported to have growth inhibitory activity^30^. It was also confirmed that PZ-HPV-7 cells barely possessed sufficient metabolic activity to produce 1,25D3 from administered 25D3 because of a lack of CYP27B1, which was confirmed by high-performance liquid chromatography and mass spectrometry experiments. This result indicates that 25D3 itself shows an anti-proliferative effect on PZ-HPV-7 cells^30^. Nevertheless, 25D3 did not show an anti-proliferative effect on LNCaP cells, which are also unable to convert 25D3 to 1,25D3^31^, implying that the anti-proliferative effect of 25D3 occurs in a cell type-specific manner. Of particular interest, the role of the VDR on the pathogenesis and outcome of prostate cancer has been discussed widely^32,33^.

In this study, we investigated the gene expression profiles and intracellular behaviour of the VDR after the administration of either vitamin D preparation to LNCaP cells to elucidate whether 25D3 showed overall hormonal activity that was similar to that of 1,25D3. First, quantitative real-time PCR (qRT-PCR), immunohistochemistry examining VDR nuclear translocation, and introduction of the corresponding small interfering RNAs (siRNAs) showed that LNCaP cells possess a functional 25D3-VDR signalling system in the absence of CYP27B1. Then, we performed microarray experiments to compare the exhaustive target gene profiles of 25D3 with those of 1,25D3.

## Results

### 25D3 regulates CYP24A1 gene expression without CYP27B1 activity

Several researchers reported that only a few prostate cells express abundant amounts of CYP27B1 protein, which can convert 25D3 to 1,25D3, while it was reported that LNCaP cells have no detectable CYP27B1 activity^24,31,34^. We reconfirmed the absence of CYP27B1 activity in LNCaP cells by using an ultrasensitive liquid chromatography-tandem mass spectrometry (LC-MS/MS) assay (see Supplemental Materials and Methods) (ASKA Pharmaceutical Co., Ltd., Tokyo, Japan). LNCaP cells were administered 10^−7^ M of 25D3 for 24 h after incubating the cells with serum-free RPMI-1640 medium minus 25D3 for 24 h. Then, the cell pellets obtained from 5.0 × 10^6^ cells and the culture medium were subjected to an LC-MS/MS assay for the quantification of vitamin D metabolites. The lower limit of detection of this assay was 1.2 × 10^−11^ M. We found that there was essentially no 1,25D3 in either the cell lysate or medium (Supplemental Table S2), indicating that LNCaP cells do not have CYP27B1 activity.

**Figure 1:**
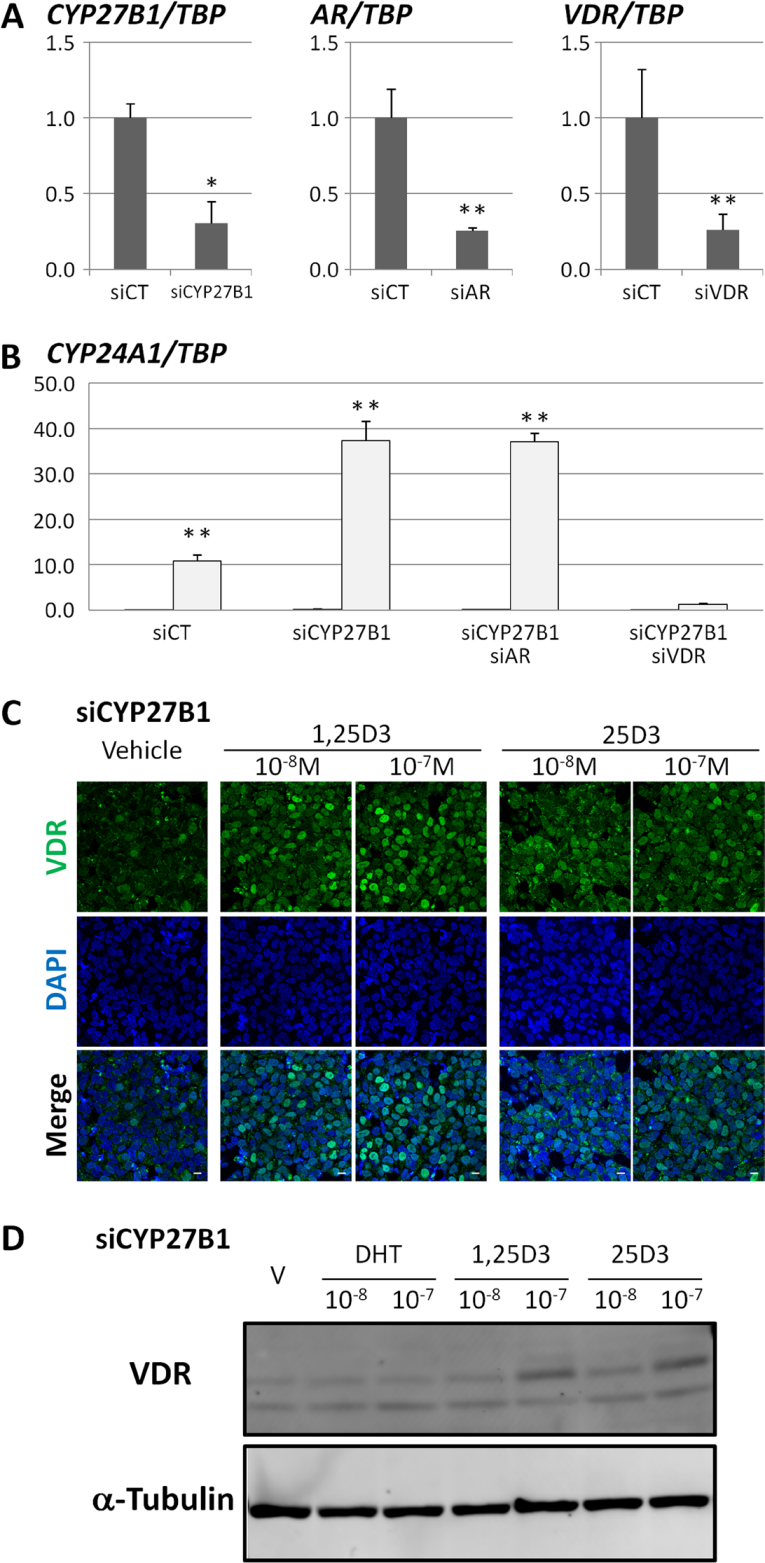
25D3 stimulates *CYP24A1* gene expression along with nuclear translocation of the VDR. (**A**) Non-targeting negative control siRNA (siCT), *CYP27B1* siRNA (siCYP27B1), *AR* siRNA (siAR), or *VDR* siRNA (siVDR) were transfected into LNCaP cells. At 24 h after transfection, *CYP27B1*, *AR*, and *VDR* expression was analysed by qRT-PCR. Expression of *TBP* was used as an internal control. Relative expression with respect to each siCT is presented. **P < 0.01 and *P < 0.05 compared with siCT. (**B**) At 24 h after the introduction of each siRNA, LNCaP cells were treated with 10^−7^ M of 25D3 for another 24 h. Expression of *CYP24A1* was analysed by qRT-PCR. Black and light grey bars indicate the results after vehicle and 25D3 treatment, respectively. Relative expression with respect to vehicle treatment is presented. **P < 0.01 compared with each vehicle. (**C**) Immunocytochemical analysis with an anti-VDR antibody was performed using siCYP27B1-introduced LNCaP cells. At 24 h after treatment with each hormone, western blotting was performed using an anti-VDR antibody. (Upper panel) VDR visualised with an Alexa 546-labelled secondary antibody (green). (Middle panel) Nuclear staining with DAPI (blue). (Lower panel) Merged images. Scale bar = 10 μm. (**D**) Western blotting of VDR proteins in LNCaP cells transfected with siCYP27B1 by using an anti-VDR antibody. An anti-α-tubulin antibody was used as a loading control. Uncropped version of the western blots are shown in Supplemental Fig. S2.

To elucidate whether 25D3 exerted physiological activity similar to that of 1,25D3, we investigated the effects of 1,25D3 and 25D3 on *CYP24A1* expression, which is a robust target gene of the 1,25D3-VDR axis, by qRT-PCR^7^. Endogenous *CYP24A1* mRNA expression in LNCaP cells transfected with non-targeting siRNA as a negative control (siCT cells) was stimulated by 1,25D3 in a dose-dependent manner (11,377-fold for 10^−9^ M, 88,243-fold for 10^−8^ M, and 933,762-fold for 10^−7^ M) (Table 1). Of note, *CYP24A1* gene expression was also up-regulated by 25D3 in a dose-dependent manner, although the dose of 25D3 required to obtain an effect comparable with that of 1,25D3 was 10–20 times higher, despite being used within its physiological range (see Discussion). Indeed, 10^−7^ M of 25D3 exerted a stimulatory effect that was several hundred-fold stronger than at 10^−8^ M and 70% weaker than at 10^−7^ M of 1,25D3 (Table 1). In the siRNA experiment, we employed *CYP27B1*-specific siRNA, which further decreased the inherently negligible amount of *CYP27B1* mRNA to 30% (Fig. 1A). In the cells transfected with siRNA specific for *CYP27B1* (siCYP27B1 cells) to dampen 1,25D3 generation more thoroughly as shown above, a concentration of 25D3 as high as 10^−7^ M again showed obvious stimulatory effects on *CYP24A1* stimulation (Table 1). These results indicated that 25D3 exerts hormonal activity on *CYP24A1* gene regulation even when there is no CYP27B1 activity in LNCaP cells.

**Table 1:** 1,25D3 and 25D3 induce *CYP24A1* expression in LNCaP cells.

siRNA	Hormone		Relative expression of *CYP24A1* toward to *TBP*
siCT	Vehicle		1 ± 1
	1,25D3	1.0 × 10^−9^ M	11377 ± 2941
		1.0 × 10^−8^ M	88243 ± 13597
		1.0 × 10^−7^ M	933763 ± 91765
	25D3	1.0 × 10^−9^ M	97 ± 123
		1.0 × 10^−8^ M	1054 ± 456
		1.0 × 10^−7^ M	242151 ± 42115
siCYP27B1	Vehicle		1254 ± 962
	1,25D3	1.0 × 10^−9^ M	85339 ± 2072
		1.0 × 10^−8^ M	565408 ± 35900
		1.0 × 10^−7^ M	4178137 ± 802982
	25D3	1.0 × 10^−9^ M	3125 ± 2986
		1.0 × 10^−8^ M	31561 ± 1706
		1.0 × 10^−7^ M	2149846 ± 383240

*CYP24A1* values are shown relative to them in vehicle-treated siCT cells.

### 25D3 induces nuclear localization of the VDR

To elucidate which nuclear receptor was involved in the activation of *CYP24A1* by 25D3, we performed co-knockdown experiments using siRNAs specific to either the androgen receptor (*AR*) or *VDR* in addition to siRNA for *CYP27B1*. We confirmed the efficient knockdown of each nuclear receptor gene by qRT-PCR; *AR* was reduced to 25% and *VDR* was reduced to 26% (Fig. 1A). We administered 10^−7^ M of 25D3 to each siRNA-transfected LNCaP cell line for 24 h, then examined *CYP24A1* expression by qRT-PCR. We found that knockdown of the VDR alone in siCYP27B1 cells, but not the AR, contributed to the disappearance of the 25D3-dependent activation of *CYP24A1* expression (Fig. 1B). These results validated our hypothesis that 25D3-dependent *CYP24A1* expression is mediated by the VDR.

Then, we analysed the effect of 25D3 on the intracellular localization of the VDR in siCYP27B1 cells by immunocytochemistry and western blotting experiments with a VDR-specific antibody (D2K6W). In vehicle-treated siCYP27B cells, intranuclear VDR staining was observed in a small number of cells (Fig. 1C). On the other hand, the VDR in cells administered 1,25D3 showed strong nuclear signals in almost all cells in a dose-dependent manner, which was reproducibly and consistently observed in 25D3-treated siCYP27B1-LNCaP cells (Fig. 1C). This nuclear accumulation of the VDR was seen when we used a dose as low as 10^−8^ M of 25D3 and 1,25D3. Western blotting experiments from each hormone-treated LNCaP cell line also confirmed that 25D3 and 1,25D3 induced the nuclear accumulation of the VDR (Fig. 1D).

### Exhaustive gene expression profiles reveal that the effects of 25D3 are almost exclusively mediated by the VDR

**Figure 2:**
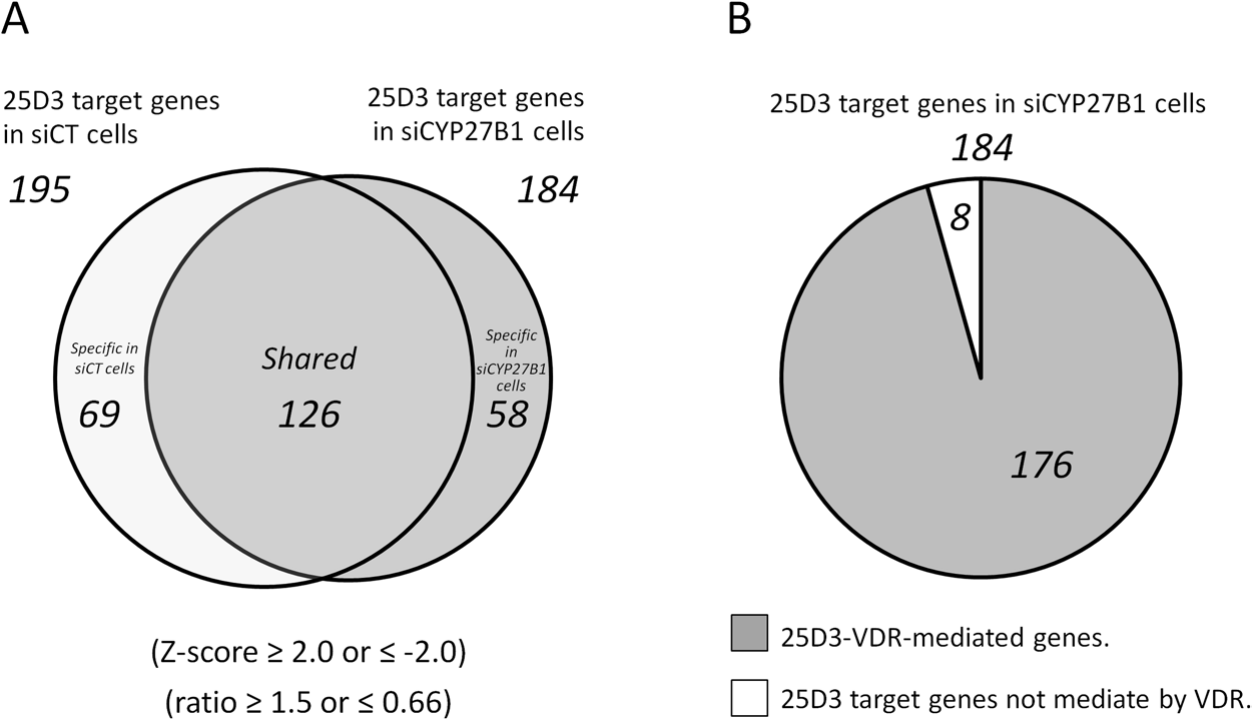
25D3 target gene profile mediated by the VDR. The exhaustive target gene profiles of 25D3 in LNCaP cells are presented. Venn diagram indicates the distribution of the 25D3 target genes in siCT cells and siCYP27B1 cells. The number of probes in each group is shown. (**A**) 25D3 target genes in siCT cells and siCYP27B1 cells were collected satisfying the conditions of a Z-score ≥ 2.0 or ≤−2.0 and ratio ≥1.5 or ≤0.66. (**B**) The proportion of genes whose regulation was mediated by the VDR in the grey area of (**A**) is presented. In this profile, genes showing a ratio ≥0.66 and ≤1.5 in doubly introduced siCYP27B1 + siVDR cells compared with vehicle-treated cells are presented in the grey area as ‘25D3-VDR-mediated target genes’.

To reveal the gene regulatory profiles induced by 25D3, we performed a series of microarray analyses. Gene expression profiles were determined using total mRNA from either siCT cells or siVDR cells, together with or without the transient introduction of siCYP27B1 followed by treatment with 10^−7^ M of 25D3 for 24 h. The Z-score and ratio of activity after 25D3 treatment were calculated. At first, we focused on the common genes induced by 25D3 in both siCT cells and siCYP27B1 cells. We isolated 25D3 target genes satisfying the Z-score criteria ≥2.0 or ≤−2.0 and a ratio ≥1.5 or ≤0.66 in both siCT and siCYP27B1 cells. As a result, 195 probes and 184 probes were isolated as 25D3 target genes in siCT cells and siCYP27B1 cells, respectively (Fig. 2A: the gene list is presented in Supplemental Table S3A and B). A Venn diagram of the target genes indicated that 126 probes were shared between siCT cells and siCYP27B1 cells (Fig. 2A: the gene list is presented in Supplemental Table S3C), while 69 targets were present specifically in siCT cells and 58 targets were present specifically in siCYP27B1 cells (Fig. 2A: the gene list is presented in Supplemental Table S3D and E), although the implications of these results, such as the notion that a concentration of 10^−7^ M of 25D3 was still too low in some cases, remain unresolved.

Then, to investigate how the VDR contributed to the gene regulation program of 25D3 in siCYP27B1 cells, we performed microarray analysis using mRNAs derived from LNCaP cells with doubly introduced siCYP27B1 and siVDR. From 184 probes of the genes regulated by 25D3 in siCYP27B1 cells (Fig. 2A), we isolated genes satisfying our ratio criteria of ≤1.5 and ≥0.66 after treatment with 10^−7^ M of 25D3 in doubly introduced siCYP27B1 + siVDR cells, and we assigned them as 25D3-VDR-mediated target genes. We found that 176 probes, 96% of the 184 species of 25D3 target genes in siCYP27B1 cells, were 25D3-VDR-mediated target genes (Fig. 2B; the gene list is presented in Supplemental Table S4A). The 8 25D3 target genes that were seemingly not regulated by the VDR (Fig. 2B) are listed in Supplemental Table S4B. These results indicated that the VDR has a crucial role as a nuclear receptor in 25D3 signalling in LNCaP cells.

### The 25D3 target gene profile is within the range of the 1,25D3 target gene profile

To verify the bioactivity of 25D3, comprehensive microarray analyses were performed using each mRNA sample from vehicle- vs 1,25D3-administered siCT cells and vehicle- vs 25D3-administered siCYP27B1 cells, respectively. We collected their target gene profiles satisfying a Z-score ≥ 2.0 or ≤−2.0 and a ratio ≥1.5 or ≤0.66. As shown in Supplemental Tables S3B and S5, 263 probes were identified as 1,25D3 target genes in siCT cells and 184 probes were identified as 25D3 target genes in siCYP27B1 cells. Gene ontology (GO) term analysis was performed using the term ‘Biological Process’, revealing that 1,25D3 and 25D3 regulated diverse GO terms. For the 1,25D3 target genes, several metallothionein (*MT*) genes (*MT1B*, *MT1E*, *MT1F*, *MT1G*, *MT1L*, *MT1X*, and *MT2A*) formed the GO terms of ‘Cellular response to zinc ion’, ‘Negative regulation of growth’, and ‘Cellular response to cadmium ion’ (Table 2). On the other hand, for the 25D3 target genes, GO terms of ‘Flavonoid biosynthetic process’ and ‘Flavonoid glucuronidation’ were distinguished and consisted of the *UGT2B* gene family (*UTG2B7*, *UGT2B10*, *UGT2B11*, and *UGT2B15*), although *UGT2B7* and *UGT2B10* were also listed in ‘lipid metabolic process’ of the 1,25D3 GO term (Table 2).

**Table 2:** GO analysis of 1,25D3 and 25D3 target genes.

Hormone	GO Term (Biological Process)	Gene Count	Gene Name	P-Value
1,25D3	Cellular response to zinc ion	7	MT1B, MT1E, MT1F, MT1G, MT1L, MT1X, MT2A	4.0E-08
	Negative regulation of growth	7	MT1B, MT1E, MT1F, MT1G, MT1L, MT1X, MT2A	4.0E-08
	Cellular response to cadmium ion	4	MT1E, MT1F, MT1G, MT1X	8.0E-04
	Negative regulation of sequence-specific DNA binding transcription factor activity	5	SMAD7, RNF2, RLIM, TRIB1, TNSF4	4.3E-03
	Regulation of inflammatory response	5	BCL6, LYN, SLC7A2, TNFSF4, ZYX	5.1E-03
	Response to metal ion	3	MT1X, MT2A, NEDD4L	5.1E-03
	Lipid metabolic process	7	STARD3, UGT2B10, UGT2B7, CLU, G6PD, GPCPD1, NPHP3	8.0E-03
	Somitogenesis	4	LFNG, NKX3.1, XRCC2, LEF1	9.0E-03
25D3	Flavonoid biosynthetic process	4	UGT2B10, UGT2B11, UGT2B15, UGT2B7	4.1E-04
	Flavonoid glucuronidation	4	UGT2B10, UGT2B11, UGT2B15, UGT2B7	5.5E-04
	Negative regulation of peptidyl-threonine phosphorylation	3	DDIT4, SMAD7, CALM1	4.6E-03
	Negative regulation of sequence-specific DNA binding transcription factor activity	4	SMAD7, ID2, TRIB1, TNFSF4	9.9E-03

**Figure 3:**
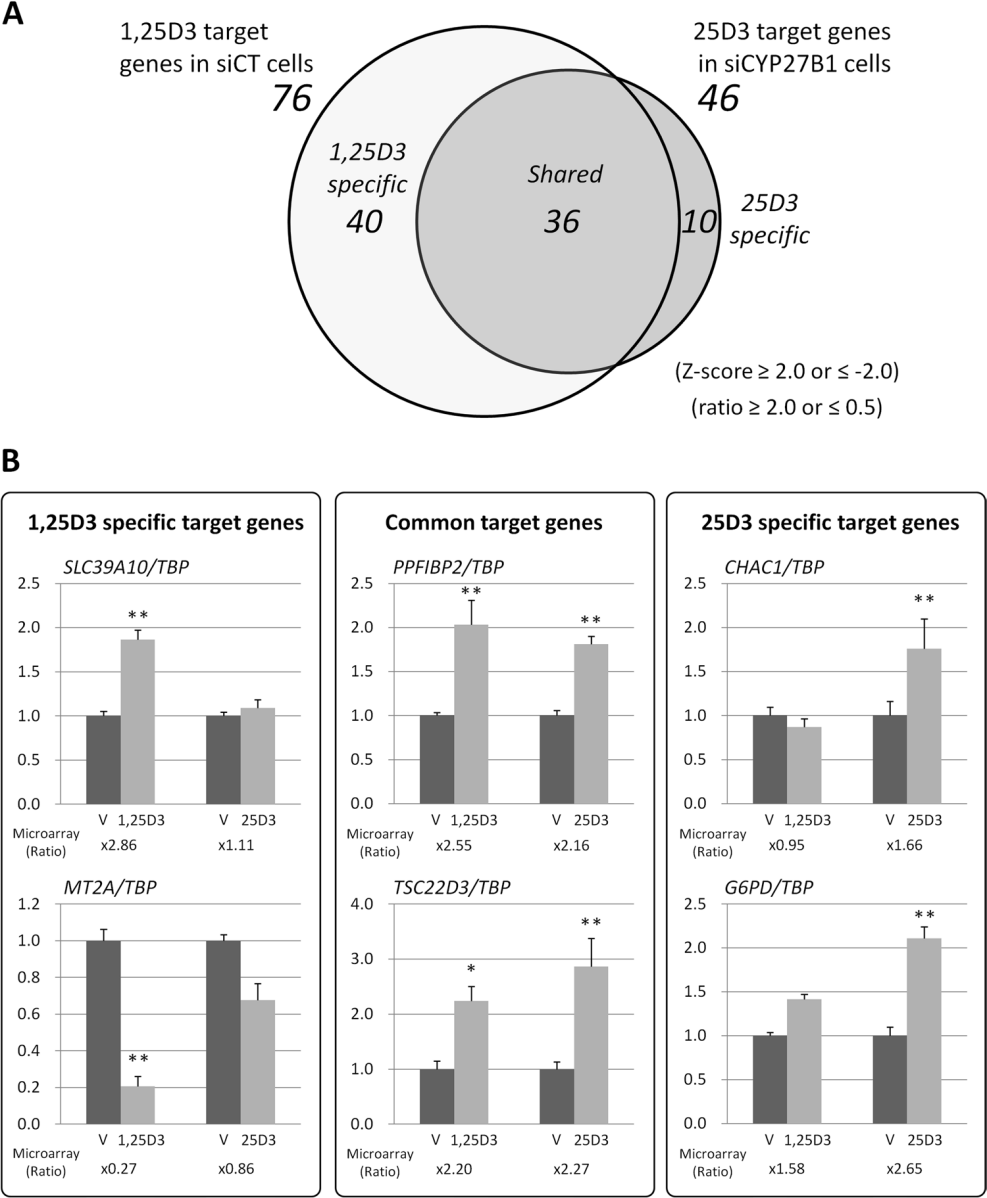
Comparison of 1,25D3 and 25D3 target gene profiles in LNCaP cells. (**A**) Venn diagram showing the distribution of 1,25D3 target genes in siCT cells and 25D3 target genes in siCYP27B1 cells. Screening condition: Z-score ≥ 2.0 or ≤−2.0 and ratio ≥2.0 or ≤0.5. (**B**) qRT-PCR analyses of some genes selected from each target population are shown. *SLC39A10* and *MT2A* were selected from the 1,25D3-specific target genes in siCT cells. *CHAC1* and *G6PD* were selected from the 25D3-specific target genes in siCYP27B1 cells. *PPFIBP2* and *TSC22D3* were selected as common target genes in both groups. Black and light grey bars indicate the results after vehicle (V) and 1,25D3 or 25D3 treatment, respectively. Each ratio calculated from the microarray data is presented below each graph. **P < 0.01 and *P < 0.05 compared with each vehicle. Data are shown relative to vehicle treatment and presented as the mean ± SD (n = 4).

To reveal the comprehensive gene regulatory profiles, we applied more strict criteria (Z-score ≥ 2.0 or ≤−2.0 and ratio ≥2.0 or ≤0.5). As shown in Fig. 3A, 76 probes were identified as 1,25D3 target genes in siCT cells and 46 probes were identified as 25D3 target genes in siCYP27B1 cells (the gene list is provided in Supplemental Table S6A and B). These two groups shared 36 probes in common (the gene list is provided in Supplemental Table S6C). Interestingly, the 25D3-specific population was small and consisted of 22% of the 25D3 target genes (Fig. 3A: 10/46; the gene list is presented in Supplemental Table S6E), while the 1,25D3-specific population consisted of approximately 53% of the 1,25D3 target genes (Fig. 3A: 40/76; the gene list is presented in Supplemental Table S6D). These results indicated that the gene profiles of 1,25D3 and 25D3 were largely overlapping, while 1,25D3 could independently regulate a certain set of genes which 25D3 could not regulate, even at the highest concentration used in the present study. We then reconfirmed the microarray results of some genes selected from each target group using qRT-PCR. For example, *SLC39A10* and *MT2A* were screened as 1,25D3-specific targets, although even 10^−7^ M of 25D3 had no discernible effect on their expression; *PPFIBP2* and *TSC22D3* were screened as common targets; and *CHAC1* and *G6PD* were assessed as 25D3-specific targets (Fig. 3B). These results supported the hypothesis that 25D3-specific targets exist, but form a small population. Again, we raise the possibility that even 10^−7^ M of 25D3 was still too low in some cases. The significance of the result that 10 genes, 2 of which (right panel of Fig. 3B) were analysed further by qRT-PCR, were 25D3-specific genes remains unsolved and is currently being investigated in our laboratory.

### Repression of *MT2A* reduces the proliferation of LNCaP cells

**Figure 4:**
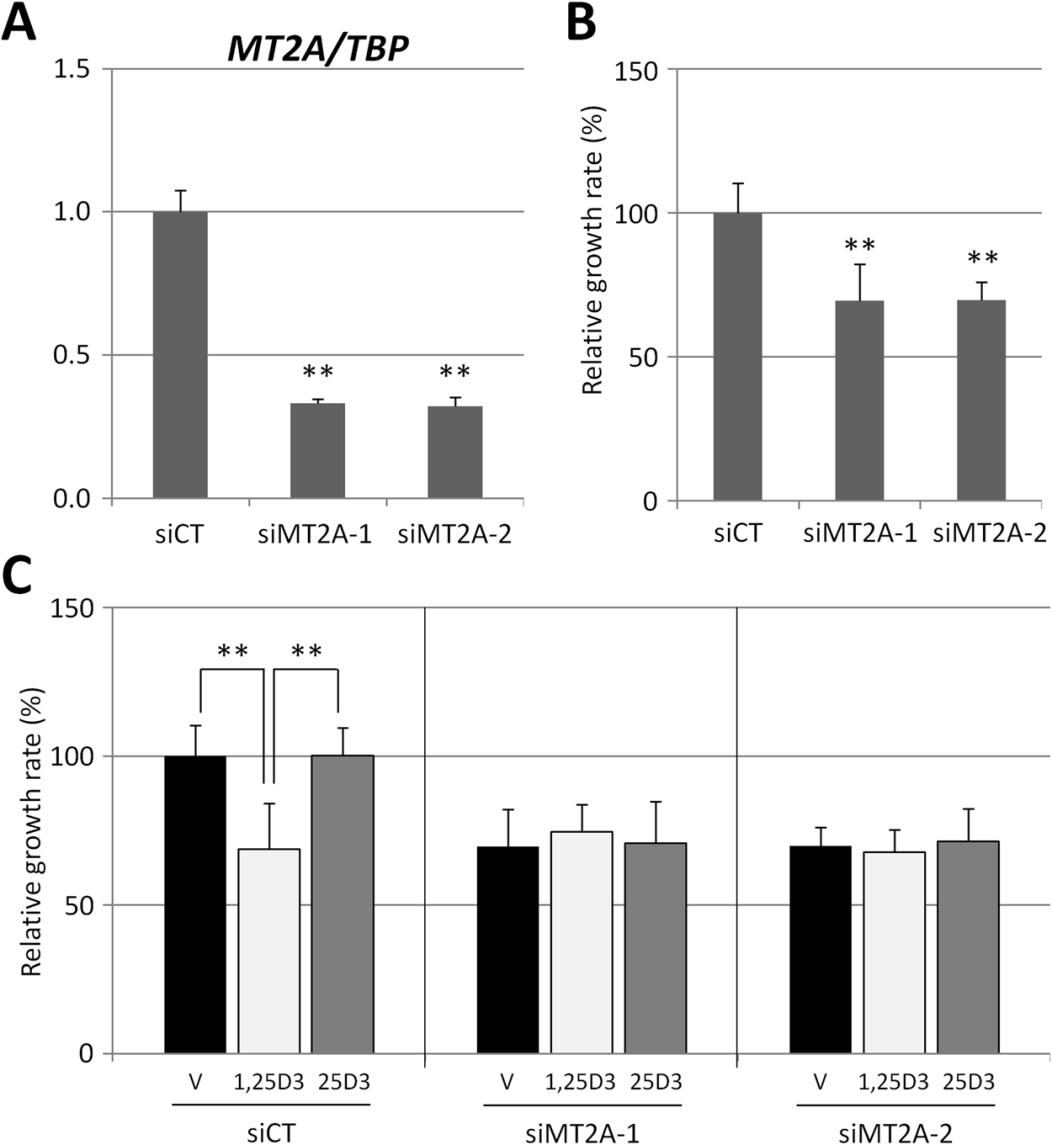
Knockdown of *MT2A* reduces the cellular proliferation of LNCaP cells. (**A**) Control siRNA (siCT) and two different *MT2A* siRNAs (siMT2A-1 and siMT2A-2) were introduced into LNCaP cells, respectively. At 24 h after transfection, *MT2A* mRNA was analysed by qRT-PCR. Data were normalised to *TBP* and presented as a relative ratio to siCT. **P < 0.01 compared with siCT. (**B**) siCT, siMT2A-1, and siMT2A-2 cells were seeded with the introduction of each siRNA in the presence of 10% CS-FBS-medium, respectively. Where indicated, relative cell numbers were counted in comparison to vehicle after incubation for 96 h. Data are shown relative to vehicle (%) and presented as the mean ± SD (n = 8). **P < 0.01 compared with vehicle. (**C**) Introduction of siRNAs and cell seeding were performed in a similar way to (**B**). After incubation for 24 h, the cells were treated with vehicle (V), 10^−7^ M of either 1,25D3 or 25D3 for another 72 h, and then the number of cells was counted. Data are shown relative to siCT-vehicle (%) and presented as the mean ± SD (n = 8). **P < 0.01.

Hitherto, we identified several *MT* genes as 1,25D3 targets (Table 2). Especially, *MT2A*, which showed the highest expression among them in LNCaP cells (Supplemental Table S7), was repressed specifically by 1,25D3, but not 25D3 (Fig. 3B). We hypothesised that this repression of *MT2A* was involved in the 1,25D3-specific anti-proliferative function in LNCaP cells because recent reports suggested that the elevated expression of *MTs* was correlated with many types of cancer and their lethality^35,36^. Quantitative reduction of *MT2A* expression by introducing two different specific siRNAs for the *MT2A* gene was performed, resulting in its expression decreasing to 33% by siMT2A-1 and 32% by siMT2A-2 (Fig. 4A). Using the cultured cells after the introduction of each siRNA for 96 h, we investigated their proliferation. As a result, both of their relative growth rates were decreased significantly to 70% compared with the proliferation of siCT cells (Fig. 4B). Subsequently, we investigated the anti-proliferative action of 1,25D3 and 25D3 in these siMT2A cells. It has been reported that 1,25D3 shows an anti-proliferative effect on the growth of LNCaP cells^31^. We confirmed that 1,25D3 decreased cell proliferation in a dose-dependent manner, while 25D3 completely lacked such an effect in LNCaP cells (Supplemental Fig. S1). siCT cells showed a significant decrement in proliferation to 69% after 1,25D3 administration (Fig. 4C). However, in both siMT2A-1 and siMT2A-2 cells, neither 1,25D3 nor 25D3 exhibited further growth repressive effects (Fig. 4C). These results suggested that repression of *MT2A* expression was important for 1,25D3 to exert its anti-proliferative action.

## Discussion

Prostate cancer is one of the most common malignancies in men. As the carcinoma advances, lethal metastasis often reduces quality of life. Aside from antiandrogens, one unanticipated, but promising, alternative is the use of vitamin D3, 1,25D3, or calcitriol, which exerts prodifferentiative, antiproliferative, and proapoptotic effects on prostate cancer cells both *in vitro* and *in vivo*^37^. To date, clinical trials of calcitriol, either alone or in combination with other chemotherapeutic agents have been conducted in patients with castrate-resistant prostate cancer^38–44^, resulting in limited antitumor benefits. More recently, the relationship between the VDR and prostate cancer has been investigated rigorously^45,46^. Conversely, the additive therapeutic role of vitamin D metabolites has been discussed widely in the treatment of oestrogen receptor-negative breast cancer in which the effects of anti-oestrogenic compounds seem to have some beneficial roles^47^.

We hypothesised that 25D3 is an active vitamin D_3_ ligand for the VDR in certain experimental conditions and cell lines. To strengthen our hypothesis, it is imperative to shut down the intracellular production of 1,25D3 by CYP27B1. It was reported that the mRNA expression and/or 1α-hydroxylase activity of CYP27B1 tend to decline during the progression of tumour development and the acquisition of higher malignant characteristics in prostate cancer^24^. Whitlatch and colleagues investigated CYP27B1 expression in several prostate tissues, including primary cultures of normal prostate, benign prostatic hyperplasia, and prostate cancer and its cell lines^34^. They found that normal prostate cells exhibited the highest expression of CYP27B1 among the tissues examined, while its expression was decreased in the following order: normal prostate, benign prostatic hyperplasia, and finally prostate cancer and its cell lines. These findings suggest that the malignant progression of prostate tissue certainly reduces *CYP27B1* expression. Indeed, some reports have shown^24,48^, and we have also confirmed, that its activity was extremely low or non-detectable in LNCaP cells using an ultrasensitive LC-MS/MS assay (Supplemental Table S2), indicating that LNCaP cells lose CYP27B1 activity. We further treated LNCaP cells with siRNA for CYP27B1 to dampen further any residual CYP27B1 activity, if any. Thus, we concluded that the series of responses induced by 25D3 observed in this study were ascribed to *bona fide* 25D3 action.

We realised that there might be a cell type-specific outcome for the anti-proliferative effect of 25D3 on prostate cancer cells. We reconfirmed that 25D3, unlike 1,25D3, lacked the anti-proliferative function in LNCaP cells (Supplemental Fig. S1) reported by others^31^. On the other hand, PZ-HPV-7 cells, which were derived from normal prostate tissue, were demonstrated to be sensitive to the anti-proliferative action of 25D3; an analogue of 25D3, 25(OH)-19-nor-D3, which is rarely subjected to 1α-hydroxylation, and 25D3 itself showed anti-proliferative effects in this cell line^30,49^. Such discrepant sensitivity to 25D3 may be due to the fact that PZ-HPV-7 cells have acquired a distinct functional machinery along with specific proliferative apparatus in response to 25D3. Gene profiling of LNCaP cells revealed that the MT genes *MT1B*, *MT1E*, *MT1F*, *MT1G*, *MT1L*, *MT1X*, and *MT2A* were repressive target genes of 1,25D3 but not 25D3 (Table 2); especially, *MT2A* was found to be markedly repressed by 1,25D3 by using qRT-PCR (Fig. 3B). MTs, which form a gene family consisting of several *MT1s*, *MT2A*, *MT3*, and *MT4*, were identified originally as heavy metal ion binding proteins and responsible for the maintenance of cellular metal homeostasis^50,51^. Recently, the expression of *MT* genes was reported to be correlated with the carcinogenesis of several tumours and tumour progression, including prostate cancer^35,36^. The robust repression of *MT2A* by 1,25D3, but not by a supraphysiological concentration of 25D3, might contribute to its selective anti-proliferation activity. We confirmed that the quantitative reduction of *MT2A* expression by the introduction of its siRNA inhibited cell proliferation. In addition, neither 1,25D3 nor 25D3 exhibited further growth repressive effects in these cells (Fig. 4C). These results indicated that repression of *MT2A* might be a prerequisite for 1,25D3 to exert its anti-proliferative action. It has been reported that *MT* genes exert their proliferative function via multiple pathways in certain cancer cells. In human prostate cancer PC-3 cells, a lack of *MT2A* was shown to induce apoptosis with the down-regulation of c-MYC and BCL-2 using a sequence-specific ribozyme technique^52^. In other reports, MTs were demonstrated to interact directly with p53, which needs a zinc atom to perform its DNA-binding and transcriptional activity^53^. MTs function as metal chelators in this complex, resulting in the repression of p53 function^54^. From this perspective, the 1,25D3-dependent repression of *MT2A* expression might lead to a gain of p53 function, resulting in the induction of p53 signalling-dependent cell cycle arrest or apoptosis.

The cellular uptake of 25D3 is believed to be greater than that of 1,25D3 due to its higher hydrophobicity. Further, 25D3 is more stable than 1,25D3; the plasma half-life of 25D3 is approximately 2 weeks, while that of 1,25D3 is less than 4 h^55–57^. Given that the plasma concentration of 25D3 is 500–1000 times higher than that of 1,25D3, some authors have proposed that such a physiological concentration of 25D3 binds to and activates the VDR. However, the concept that a 1/500 ratio of total hormones can directly correspond to free active hormone is unlikely owing to the variable concentrations of vitamin D-binding protein (DBP). As it is known that DBP binds to 25D3 with a higher affinity than to 1,25D3, the ratio of free 1,25D3 to free 25D3 might be higher than the 1/500–1/1000 ratio of total 1,25D3 to 25D3. As such, an estimation based on both the concentration of DBP and the relative affinity of both metabolites to the VDR gives approximate concentrations of 10 and 1 pM for free 25D3 and 1,25D3, respectively^58^. Indeed, 10^−8^ M of 1,25D3 had a stimulatory effect on *CYP24A1* expression, which was only 3-4-fold lower than that after treatment with 10^−7^ M of 25D3 (Table 1). Thus, from the physiological viewpoint, 25D3 can bind to and activate the VDR *in vitro*. *In vivo*, relevant evidence for a direct effect of 25D3 is provided by the ability of high-dose cholecalciferol to prevent osteomalacia and hypocalcaemia in 1-α-hydroxylase knockout mice^59,60^. In another example, it was shown that extracellular matrix mineralization was induced by 25D3, but not by 1,25D3, leading the authors to reach a conclusion that 25D3, despite its lower affinity to the VDR, can stabilise the VDR-ligand binding domain in its agonistic conformation in the same way as 1,25 D3 does^14^.

Hewison’s group reported recently that high muscle strength was provided by 25D3, but not by 1,25D3^61^. In their report, there was no significant correlation between the serum levels of 25D3 and 1,25D3. In contrast, serum 25(OH)D_3_, 24,25(OH)_2_D_3_, and 3-epi-25(OH)D_3_ levels were strongly correlated. They also reported that parallel analysis of *CYP27B1* in muscle biopsies did not reveal the significant expression of this gene, suggesting that the localised conversion of 25(OH)D_3_ to 1α,25(OH)_2_2D_3_ is not a prominent feature of muscle, as was reported for the LNCaP cells shown here. Although they did not examine whether the 25D3-VDR axis functions in muscle, they found that out of the 92 genes examined, the expression of 24 skeletal muscle genes was strongly correlated with serum 25D3. These genes are involved in hormonal/intracellular signalling, cell stress, proteasomal activity, protein translation, amino acid metabolism, muscle contraction, myogenesis, and mitochondrial function. 1,25D3 was also associated with the expression of genes involved in skeletal muscle metabolism, protection against cell stress, and protein degradation. Thus, there might be another signal transduction system via which the effect of 25D3 is exerted by a non-VDR-mediated pathway in muscle.

Among the genes whose expression was affected by the single action of either 25D3 or 1,25D3 as well as their combined action in prostatic cancer LNCaP cells, we could not find any commonality, except that approximately 47% (Fig. 3A: 36/73) of the 1,25D3 target genes were also under the control of 25D3. We could not resolve the possibility that a higher dose of 25D3 than used in this study might complement the 1,25D3 target gene profile completely. *CYP24A1* shown here is a typical example (Table 1). Altogether, in LNCaP cells, we compared the individual target gene profiles of 1,25D3 and 25D3 to clarify their interrelationship. We found that 25D3 largely regulated the expression of its target genes in common with 1,25D3 via the VDR when the VDR is expressed in prostate cancer cells, although the anti-proliferative activity of 25D3 was absent. We surmise that the ability to suppress the expression of *MT* genes may be one of the most important mechanisms for 1,25D3, but not 25D3, to exert its anti-proliferative action. Further study is warranted to examine the effectiveness of 25D3 in combating prostate cancer.

## Methods

### Cell cultures and hormones

Monolayer culture and maintenance of LNCaP cells were described previously^62^. For the proliferation assay, RPMI phenol red-free medium containing 10% charcoal-stripped foetal bovine serum with antibiotics (10% CS-FBS-medium) was used. 1α,25(OH)_2_D_3_ (referred to as 1,25D3) and 25(OH)D_3_ (referred to as 25D3) were supplied by DSM (Het Overloon, Netherlands).

### siRNA transfection and qRT-PCR

Non-targeting siRNA and a mixture of four different siRNAs specific for *VDR* were obtained from Dharmacon (Thermo Fisher Scientific, Waltham, MA). siRNA for *CYP27B1* was obtained from Invitrogen (Thermo Fisher Scientific). siRNAs for *MT2A* (MT2A-1 and MT2A-2) were obtained from Sigma Aldrich (Merck KGaA, Darmstadt, Germany). Transfection of siRNA using Lipofectamine RNAiMAX (Thermo Fisher Scientific) was described previously^62^. Methods for the isolation of total RNA from cells, synthesis of cDNA, and qRT-PCR were also described previously^62,63^. The primer sets used in this study are shown in Supplemental Table S1. Data were normalised to the expression of TATA-binding protein (*TBP*) and presented relative to the result of vehicle ethanol treatment. Values are expressed as the mean ± standard deviation (SD) (n = 4).

### Western blotting

The details of western blotting analysis were described previously^62^. For the detection of VDR-specific immunobands, a rabbit antibody against human VDR diluted at 1:1000 (D2K6W; Cell Signaling Technology, Danvers, MA) was used. The anti-VDR antibody used in this study recognised N-terminal amino acid residues, resulting in a reaction with both VDRB1 and VDRB2 isoforms.

### Immunocytochemistry

The details of immunocytochemistry were described previously^62^. A rabbit antibody against human VDR diluted at 1:2000 (D2K6W; Cell Signaling Technology) was used.

### Microarray analysis

Isolation of total RNA from siCT, siCYP27B1, or siVDR cells, which were treated previously with 10^−7^ M of 25D_3_ or 1,25D3 for another 24 h, was performed using an RNeasy Mini Kit (QIAGEN, Hilden, Germany). Using RNA samples, cDNA microarray analysis was performed by APRO Life Science Institute (Tokushima, Japan) and Cell Innovator (Fukuoka, Japan) as described previously^62^. The GEO accession number is GSE107438. We selected the probes that called the ‘P’ flag and the cut-off signal was smaller than 100. To identify regulated genes, we calculated the Z-score and the ratio (non-log scaled fold-change) for comparisons between each vehicle- and hormone-treatment sample.

### Statistical analysis

Data are expressed as the mean ± SD of quadruplicate assay, unless otherwise stated. Student’s *t*-test (Fig. 1A) or one-factor analysis of variance and Tukey’s test (Figs 1B, 3B and 4 and S1) were carried out.

### Data availability

The datasets generated during and/or analysed during the current study are available from the corresponding author on reasonable request.

## Electronic supplementary material

Supplementary Information
