# Supplementary material for: Without 1α-hydroxylation, the gene expression profile of 25(OH)D3 treatment overlaps deeply with that of 1,25(OH)2D3 in prostate cancer cells
Source: Sci Rep. 2018 Jun 13;8:9024. doi: 10.1038/s41598-018-27441-x (PMC5998076; doi:10.1038/s41598-018-27441-x)

Supplementary Information

Title

Without 1α-hydroxylation, the gene expression profile of 25(OH)D_3 treatment overlaps deeply with that of 1,25$(OH)_2D_3$ in prostate cancer cells

Authors/Affiliations

Takao Susa[1], Masayoshi Iizuka[1], Hiroko Okinaga[2], Mimi Tamamori-Adachi[1], and Tomoki Okazaki[1*]

[1] Department of Biochemistry, Teikyo University School of Medicine, 2-11-1 Kaga, Itabashi-ku, Tokyo, 173-8605, Japan

[2] Departments of Internal Medicine, Teikyo University School of Medicine, 2-11-1 Kaga, Itabashi-ku, Tokyo, 173-8605, Japan

* Corresponding author: okbgeni@med.teikyo-u.ac.jp

Supplementary Information:

Supplemental methods, Supplemental Tables S1-S7, and Supplemental Figs. S1-S2.

Supplemental methods

LC-MS/MS experiment for the quantification of metabolized 1,25D3 in LNCaP cell.

Before sample preparation, a cell precipitation was suspended in 0.5 ml of distilled water by Ultra-Turrax homogenizer (IKA, Staufen, Germany). As internal standards, 200 pg of $^{2}H_6$-1,25-$(OH)_2D_3$, and was added to the cell suspensions and cell cultured medium (0.2 mL), respectively. The sample was added to acetonitrile (MeCN) (2 ml), vortex-mixed and centrifuged. The supernatant was evaporated to dryness, and then, the residue was added to 1 ml of 1 M sodium hydroxide (NaOH) solution. 1,25-$(OH)_2D_3$ and 25-OHD_3 were extracted by 4 ml of hexane/methyl *tert*-butyl ether (MTBE) (4:1). After the organic layer was evaporated to dryness, the extracts were dissolved in 0.5 ml of MeCN and diluted with 1 ml of distilled water, and then applied to Oasis MAX cartridge (Waters Co., Milford, MA, USA) which had been successively conditioned with 3 ml of methanol, 3 ml of distilled water. The cartridge was washed with 1 ml of 0.2 M NaOH solution, 2 ml of distilled water and 1 ml of MeCN/distilled water (2:3) solution, and then, 1,25-$(OH)_2D_3$ was eluted with 1 ml of MeCN/MTBE (3:2). After evaporation, each the residue was subjected to derivatization described below. The sample was reacted with 50 μl of 0.5 mg 4-phenyl-1,2,4-triazole-3,5-dione in MeCN for 30 minutes at room temperature. After the reaction, the sample was dissolved in 0.5 ml of ethyl acetate/hexane/acetic acid (15:35:1) and the mixture was applied to InertSep SI cartridge (GL sciences Int., Tokyo, Japan) which had been successively conditioned with 3 ml of acetone and 3 ml of hexane. The cartridge was washed with 0.5 ml of hexane and 2 ml of acetone/hexane (2:3), and then the sample was eluted with 2.5 ml of acetone/hexane (4:1). After evaporation, the residue was dissolved in 0.1 ml of acetonitrile/ distilled water (2:3) and 0.02 ml of the solution was subjected to an LC-MS/MS instrument which was described below. An API-4000 triple stage mass spectrometer equipped with an ESI ion source (SCIEX, Framingham, MA, USA) and Nexera UHPLC systems (Shimadzu Co. Ltd., Kyoto, Japan) was employed. ACQUITY UPLC HSS C18 SB column (1.8 μm, 150×2.1 mm i.d. , Waters Co., Milford, MA, USA) was used at 40°C. The mobile phase consisting of 0.1% formic acid (Solvent A) and acetonitorile (Solvent B) was used with a gradient elution of A:B=65:35 to 50:50 (0 - 1.0 min), 50:50 to 25:75 (1.0 - 3.0 min), 25:75 to 20:80 (3.0 - 5.0 min), 20:80 to 0:100 (5.0 - 5.9 min), 0:100 (5.9 - 6.4 min) and 40:60 (6.4 - 7.0 min) at a flow rate of 0.45 ml/min. the transitions of *m/z* 574.5/314.0 and 580.2/314.2 were selected for 1,25-$(OH)_2D_3$ and $^{2}H_6$-1,25-$(OH)_2D_3$, respectively. The lower limits of quantification of 1,25-$(OH)_2D_3$ was 1 pg/tube.

Cell proliferation assay

For the cell proliferation assay, LNCaP cells approximately (approximately 3.0×10^4

cells/100 μL/well) were seeded in 96-well plates in 10% CS-FBS-medium. After incubation for 24 h, the cells were treated with each steroid hormone in 10% CS-FBS-medium for another 144 h, and then the number of cells was counted. Relative cell numbers are presented with respect to vehicle treatment. The means ± SD was calculated after quadruplicate assays. Statistical analysis was carried out using one-factor analysis of variance and Tukey's test. *$P < 0.05$ compared with each vehicle.

Supplemental Table S1. Primer sets used for real-time PCR.

Gene	Forward	Reverse
CYP27B1	TTGGCAAGCGCAGCTGTAT	TGTGTTAGGATCTGGGCCAAA
VDR	CACTATTCACCTGCCCCTTC	CTCCCTCCACCATCATTCAC
AR	CCTGGCTTCCGCAACTTACAC	GGACTTGTGCATGCGGTACTC
SLC39A10	TGCAGGCATGTTCCTCTATG	CCACAGGACAAAAGCCATGT
MT2A	GACTCTAGCCGCCTCTTCAG	*TGCATTTGCACTCTTTGCAT*
PPFIBP2	TGACCACCACAGCCAAAGT	GGCAAATGTAGTCCGTCGAT
TSC22D3	TGGTGGCCATAGACAACAAG	TGCTCCTTCAGGATCTCCAC
CHAC1	AGGGAGACACCTTCCATCG	GTATGCCACGCCCCAAGT
G6PD	CCAGATTGAGCTGGAGAAGC	TGGAAACCCACTCTCTTCATC
TBP	GTTCTGGGAAAATGGTGTGC	GCTGGAAAACCCAACTTCTG

Supplemantl Table S2. LC-MS/MS for the quantification of 1,25D3 in LNCaP cell.

Sample	siRNA	Used Hormone	1α,25(OH)2D3
Cell lysate	siCT	25OHD3 (10^{-7} M)	N.D. (mol)
Cell lysate	siCYP27B1	25OHD3 (10^{-7} M)	N.D. (mol)
Culture media	siCT	25OHD3 (10^{-7} M)	N.D. (M)
Culture media	siCYP27B1	25OHD3 (10^{-7} M)	N.D. (M)

N.D.: not detected

Supplemental Table S3 Gene list of 25D3 target genes in LNCaP cells.

Supplemental Table S3A 25D3 target genes in siCT cells (195).

ProbeID	GeneSymbol	siCT_V1_signal	siCT_1,25D3_signal	siCT_V2_signal	siCT_25D3_signal	siCYP27B1_V_signal	siCYP27B1_25D3_signal	siCYP27B1_siVDR_V_signal	siCYP27B1_siVDR_25D3_signal	siCT_1,25D3/siCT_V1_Zscore	siCT_1,25D3/siCT_V1_ratio	siCT_25D3/siCT_V2_Zscore	siCT_25D3/siCT_V2_ratio	siCYP27B1_25D3/siCYP27B1_V_Zscore	siCYP27B1_25D3/siCYP27B1_ratio	siCYP27B1_siVDR_25D3/siCYP27B1_siVDR_V_Zscore	siCYP27B1_siVDR_25D3/siCYP27B1_siVDR_V_ratio	siCYP27B1_25/V/siCT_25D3/V2_ratio	siCYP27B1_siVDR_25D3/V/siCYP27B1_25D3/V_ratio	siC27B1_25D3/V/siCT_1,25D3/V1_ratio	siCT_V1_flag	siCT_1,25D3_flag	siCT_V2_flag	siCT_25D3_flag	siCYP27B1_V_flag	siCYP27B1_25D3_flag	siCYP27B1_siVDR_V_flag	siCYP27B1_siVDR_25D3_flag	PrimaryAccession
A_21_P0011866	RPS2	180193	185340	133545	213992	140491	149477	136172	141974	0.69	1.03	6.81	1.60	0.90	1.06	1.02	1.04	0.66	0.98	1.03	P	P	P	P	P	P	P	P	NM_002952
A_23_P16523	GDF15	40397	108529	141974	269139	102700	185340	60947	93882	12.33	2.69	9.24	1.90	8.54	1.80	5.54	1.54	0.95	0.85	0.67	P	P	P	P	P	P	P	P	NM_004864
A_33_P3285545	CLDN4	31397	48717	50313	80442	38373	64904	38181	45207	5.47	1.55	5.94	1.60	6.16	1.69	2.15	1.18	1.06	0.70	1.09	P	P	P	P	P	P	P	P	NM_001305
A_23_P106844	MT2A	27944	7668	29692	14177	30083	25807	33511	24943	-11.66	0.27	-7.40	0.48	-1.81	0.86	-3.83	0.74	1.80	0.87	3.13	P	P	P	P	P	P	P	P	NM_005953
A_24_P208345	SLC45A3	11280	16965	11201	20087	9303	17392	7790	8784	3.68	1.50	5.87	1.79	5.98	1.87	0.69	1.13	1.04	0.60	1.24	P	P	P	P	P	P	P	P	NM_033102
A_33_P3233645	MT1G	10806	2088	6493	2983	9896	7785	8926	6027	-11.83	0.19	-5.96	0.46	-2.27	0.79	-2.16	0.68	1.71	0.86	4.07	P	P	P	P	P	P	P	P	NM_005950
A_33_P3220837	MAFB	9972	16102	14410	22495	22148	32617	16495	17917	4.32	1.61	4.48	1.56	4.53	1.47	0.48	1.09	0.94	0.74	0.91	P	P	P	P	P	P	P	P	NM_005461
A_23_P427703	MT1L	8823	1995	5265	2586	6471	5358	6418	4653	-10.70	0.23	-5.45	0.49	-1.78	0.83	-5.14	0.73	1.69	0.88	3.66	P	P	P	P	P	P	P	P	NR_001447
A_23_P37983	MT1B	8565	2176	6045	2895	7709	6365	7465	5245	-9.86	0.25	-5.64	0.48	-1.81	0.83	-1.94	0.70	1.72	0.85	3.25	P	P	P	P	P	P	P	P	NM_005947
A_23_P113111	AR	7199	19386	7131	19811	7680	22300	8545	12200	8.93	2.69	10.26	2.78	10.18	2.90	2.00	1.43	1.05	0.49	1.08	P	P	P	P	P	P	P	P	NM_000044
A_33_P3315314	MT1HL1	7062	2098	5552	2867	7058	5971	6508	4742	-8.73	0.30	-5.07	0.52	-1.58	0.85	-5.06	0.73	1.64	0.86	2.85	P	P	P	P	P	P	P	P	NM_001276687
A_23_P206724	MT1E	7058	1617	4099	1958	5511	4716	4990	3578	-10.60	0.23	-5.66	0.48	-1.14	0.86	-5.31	0.72	1.79	0.84	3.73	P	P	P	P	P	P	P	P	NM_175617
A_23_P69573	GUCY1A3	6022	4366	9759	5702	8708	5006	8498	8051	-2.31	0.73	-5.37	0.58	-5.26	0.57	-0.28	0.95	0.98	1.65	0.79	P	P	P	P	P	P	P	P	NM_000856
A_33_P3350056	MT1X	5959	2087	3706	2150	4575	4111	4709	3490	-7.55	0.35	-4.18	0.58	-0.79	0.90	-4.79	0.74	1.55	0.82	2.57	P	P	P	P	P	P	P	P	NM_005952
A_24_P252497	TRIB1	5552	11989	6847	14438	5608	11955	4526	6993	6.94	2.16	7.50	2.11	7.24	2.13	6.94	1.54	1.01	0.72	0.99	P	P	P	P	P	P	P	P	NM_025195
A_23_P382188	STAP2	4876	7721	4088	7511	5905	9139	4463	4569	4.15	1.58	4.65	1.84	4.18	1.55	0.37	1.02	0.84	0.66	0.98	P	P	P	P	P	P	P	P	NM_001013841
A_23_P401568	MAT2A	4795	7941	4686	8200	5471	8029	5492	5608	4.55	1.66	5.63	1.75	3.68	1.47	0.33	1.02	0.84	0.70	0.89	P	P	P	P	P	P	P	P	NM_005911
A_21_P0012331		4424	11175	2797	7283	1774	7627	2929	3406	8.36	2.53	7.32	2.60	10.65	4.30	2.40	1.16	1.65	0.27	1.70	P	P	P	P	P	P	P	P	ENST00000546734
A_33_P3288364	SPATA2L	4122	6216	3066	4634	2556	3735	2749	2932	2.96	1.51	3.16	1.51	2.77	1.46	1.03	1.07	0.97	0.73	0.97	P	P	P	P	P	P	P	P	NM_152339
A_23_P87560	BTG1	3886	9903	3652	8522	6035	9194	5010	6221	8.44	2.55	6.48	2.33	4.03	1.52	3.45	1.24	0.65	0.82	0.60	P	P	P	P	P	P	P	P	NM_001731
A_23_P164179	TOB1	3853	2866	3298	2154	2738	1755	2501	2249	-2.13	0.74	-3.27	0.65	-3.25	0.64	-1.70	0.90	0.98	1.40	0.86	P	P	P	P	P	P	P	P	NM_005749
A_33_P3239347	NKX3-1	3733	6664	1639	3527	2511	4842	2007	2107	4.17	1.79	5.86	2.15	4.79	1.93	0.77	1.05	0.90	0.54	1.08	P	P	P	P	P	P	P	P	NM_006167
A_33_P3358158	CRYBB2P1	3632	3431	2138	3223	5398	3912	3767	3299	-0.41	0.94	3.14	1.51	-2.36	0.72	-2.12	0.88	0.48	1.21	0.77	P	P	P	P	P	P	P	P	NR_033733
A_33_P3237634	TSC22D3	3595	7923	3169	7926	2443	6807	1953	2526	5.69	2.20	7.01	2.50	7.48	2.79	4.11	1.29	1.11	0.46	1.26	P	P	P	P	P	P	P	P	NM_004089
[illegible]	[illegible]	[illegible]	[illegible]	[illegible]	[illegible]	[illegible]	[illegible]	[illegible]	[illegible]	[illegible]	[illegible]	[illegible]	[illegible]	[illegible]	[illegible]	[illegible]	[illegible]	[illegible]	[illegible]	[illegible]	[illegible]	[illegible]	[illegible]	[illegible]	[illegible]	[illegible]	[illegible]	[illegible]	[illegible]

Supplemental Table S3B 25D3 target genes in siCYP27B1 cells (184).

ProbeID	GeneSymbol	siCT_V1_signal	siCT_1,25D3_signal	siCT_V2_signal	siCT_25D3_signal	siCYP27B1_V_signal	siCYP27B1_25D3_signal	siCYP27B1_siVDR_V_signal	siCYP27B1_siVDR_25D3_signal	siCT_1,25D3/siCT_V1_Zscore	siCT_1,25D3/siCT_V1_ratio	siCT_25D3/siCT_V2_Zscore	siCT_25D3/siCT_V2_ratio	siCYP27B1_25D3/siCYP27B1_V_Zscore	siCYP27B1_25D3/siCYP27B1_ratio	siCYP27B1_siVDR_25D3/siCYP27B1_siVDR_V_Zscore	siCYP27B1_siVDR_25D3/siCYP27B1_siVDR_V_ratio	siCYP27B1_25/V/siCT_25D3/V2_ratio	siCYP27B1_siVDR_25D3/V/siCYP27B1_25D3/V_ratio	siC27B1_25D3/V/siCT_1,25D3/V1_ratio	siCT_V1_flag	siCT_1,25D3_flag	siCT_V2_flag	siCT_25D3_flag	siCYP27B1_V_flag	siCYP27B1_25D3_flag	siCYP27B1_siVDR_V_flag	siCYP27B1_siVDR_25D3_flag	PrimaryAccession
[illegible]	[illegible]	[illegible]	[illegible]	[illegible]	[illegible]	[illegible]	[illegible]	[illegible]	[illegible]	[illegible]	[illegible]	[illegible]	[illegible]	[illegible]	[illegible]	[illegible]	[illegible]	[illegible]	[illegible]	[illegible]	[illegible]	[illegible]	[illegible]	[illegible]	[illegible]	[illegible]	[illegible]	[illegible]	[illegible]

Supplemental Table S3C Common target genes between 25D3 in siCT and in siCYP27B1 (126).

ProbeID	GeneSymbol	siCT_V1_signal	siCT_1,25D3_signal	siCT_V2_signal	siCT_25D3_signal	siCYP27B1_V_signal	siCYP27B1_25D3_signal	siCYP27B1_siVDR_V_signal	siCYP27B1_siVDR_25D3_signal	siCT_1,25D3/siCT_V1_Zscore	siCT_1,25D3/siCT_V1_ratio	siCT_25D3/siCT_V2_Zscore	siCT_25D3/siCT_V2_ratio	siCYP27B1_25D3/siCYP27B1_V_Zscore	siCYP27B1_25D3/siCYP27B1_ratio	siCYP27B1_siVDR_25D3/siCYP27B1_siVDR_V_Zscore	siCYP27B1_siVDR_25D3/siCYP27B1_siVDR_V_ratio	siCYP27B1_25/V/siCT_25D3/V2_ratio	siCYP27B1_siVDR_25D3/V/siCYP27B1_25D3/V_ratio	siC27B1_25D3/V/siCT_1,25D3/V1_ratio	siCT_V1_flag	siCT_1,25D3_flag	siCT_V2_flag	siCT_25D3_flag	siCYP27B1_V_flag	siCYP27B1_25D3_flag	siCYP27B1_siVDR_V_flag	siCYP27B1_siVDR_25D3_flag	PrimaryAccession
[illegible]	[illegible]	[illegible]	[illegible]	[illegible]	[illegible]	[illegible]	[illegible]	[illegible]	[illegible]	[illegible]	[illegible]	[illegible]	[illegible]	[illegible]	[illegible]	[illegible]	[illegible]	[illegible]	[illegible]	[illegible]	[illegible]	[illegible]	[illegible]	[illegible]	[illegible]	[illegible]	[illegible]	[illegible]	[illegible]

A_24_P20630	LEF1	1460	767	3361	1610	3217	1697	2461	2111	-4.34	0.53	-5.64	0.48	-4.68	0.53	-2.45	0.86	1.10	1.63	1.00	P	P	P	P	P	P	P	P	NM_016269
A_21_P0005618		1351	2562	5443	9053	6931	10666	3909	4792	4.61	1.90	5.12	1.66	4.13	1.54	3.24	1.23	0.93	0.80	0.81	P	P	P	P	P	P	P	P	ENST00000561761
A_24_P50753	NUDT4	1299	926	1683	1005	1388	854	1221	1090	-2.29	0.71	-3.78	0.60	-3.41	0.62	-1.46	0.89	1.03	1.45	0.86	P	P	P	P	P	P	P	P	NM_199040
A_24_P80135	PTPN18	1215	1735	1338	2043	1167	1954	1393	1569	2.40	1.43	3.24	1.53	3.76	1.67	1.54	1.13	1.10	0.67	1.17	P	P	P	P	P	P	P	P	NM_014369
A_23_P395172	ABHD2	1165	1999	1267	2007	1176	1923	1145	1349	3.63	1.72	3.52	1.58	3.59	1.64	2.12	1.18	1.03	0.72	0.95	P	P	P	P	P	P	P	P	NM_007011
A_23_P45361	GLUD2	1125	2124	1198	2110	942	1590	987	1144	4.58	1.89	4.33	1.76	3.70	1.69	1.91	1.16	0.96	0.69	0.89	P	P	P	P	P	P	P	P	NM_012084
A_23_P7172	PGM2	1091	2050	1968	3638	1863	3271	1802	2193	4.24	1.88	4.70	1.85	4.11	1.76	3.13	1.22	0.95	0.69	0.93	P	P	P	P	P	P	P	P	NM_018290
A_33_P3269110	GUCY1A3	1047	763	1632	945	1385	817	1522	1386	-2.14	0.73	-4.00	0.58	-3.71	0.59	-1.20	0.91	1.02	1.54	0.81	P	P	P	P	P	P	P	P	NM_001130687
A_23_P381102	CCDC74B	934	1695	155	290	102	194	115	127	4.01	1.81	3.66	1.88	3.25	1.90	0.97	1.11	1.01	0.58	1.05	P	P	P	P	P	P	P	P	NM_207310
A_23_P394567	KIAA1467	934	1308	726	1123	430	679	539	577	2.26	1.40	3.20	1.55	3.25	1.58	0.88	1.07	1.02	0.68	1.13	P	P	P	P	P	P	P	P	NM_020853
A_33_P3368785	DLGAP1-AS1	913	1562	992	2083	1046	2120	721	1002	3.61	1.71	5.44	2.10	5.16	2.03	4.24	1.39	0.97	0.68	1.19	P	P	P	P	P	P	P	P	NR_024101
A_21_P0002781	LINC01213	901	1310	1454	2595	1087	1726	1084	1233	2.51	1.45	4.43	1.78	3.37	1.59	1.67	1.14	0.89	0.72	1.09	P	P	P	P	P	P	P	P	NR_110167
A_23_P29067	TMPRSS2	900	21619	469	11703	478	8022	430	1637	22.88	24.01	24.63	24.96	20.59	16.77	17.24	3.81	0.67	0.23	0.70	P	P	P	P	P	P	P	P	NM_005656
A_23_P210581	KCNG1	875	2016	491	1035	876	1671	884	1149	5.62	2.30	5.47	2.11	4.57	1.91	3.39	1.30	0.90	0.68	0.83	P	P	P	P	P	P	P	P	NM_002237
A_33_P3409159	SLC22A23	863	1634	718	1191	707	1217	617	717	4.30	1.89	3.72	1.66	3.84	1.72	1.95	1.16	1.04	0.68	0.91	P	P	P	P	P	P	P	P	NM_015482
A_33_P3314386	TOR1AIP1	806	1496	937	1434	957	1699	719	849	4.16	1.86	3.12	1.53	4.06	1.77	2.14	1.18	1.16	0.66	0.96	P	P	P	P	P	P	P	P	NM_001267578
A_23_P103011	RAB36	802	449	789	489	568	335	616	546	-3.92	0.56	-3.51	0.62	-3.70	0.59	-1.55	0.89	0.95	1.50	1.05	P	P	P	P	P	P	P	P	NM_004914
A_23_P31407	AGR2	764	294	268	134	278	156	350	296	-6.44	0.38	-3.99	0.50	-2.92	0.56	-1.53	0.85	1.11	1.52	1.45	P	P	P	P	P	P	P	P	NM_006408
A_33_P3292028	DPP9	759	1563	769	1352	681	1214	759	862	4.85	2.06	4.14	1.76	4.09	1.78	1.65	1.14	1.01	0.64	0.87	P	P	P	P	P	P	P	P	NM_139159
A_23_P65307	SLITRK6	758	333	1314	660	1265	752	1200	1055	-5.55	0.44	-5.04	0.50	-3.66	0.59	-1.65	0.88	1.18	1.48	1.35	P	P	P	P	P	P	P	P	NM_032229
A_23_P93269	ZNF165	750	2237	828	2020	914	2085	917	1026	7.36	2.98	6.55	2.44	6.02	2.28	1.45	1.12	0.93	0.49	0.76	P	P	P	P	P	P	P	P	NM_003447
A_23_P140450	SLC27A2	747	1116	922	1408	934	1468	1114	1160	2.70	1.49	3.11	1.53	3.20	1.57	0.53	1.04	1.03	0.66	1.05	P	P	P	P	P	P	P	P	NM_003645
A_23_P394064	PTRF	738	921	246	369	316	488	189	182	1.49	1.25	2.36	1.50	3.08	1.54	-0.36	0.96	1.03	0.62	1.24	P	P	P	P	P	P	P	P	NM_012232
A_23_P139418	GALNT18	689	2079	1218	3488	1373	3558	1139	1520	7.43	3.02	8.05	2.86	6.95	2.59	3.72	1.33	0.91	0.51	0.86	P	P	P	P	P	P	P	P	NM_198516
A_21_P0012258	MAD1L1	678	2240	528	1145	464	1082	426	555	8.04	3.30	5.68	2.17	5.98	2.33	3.42	1.30	1.08	0.56	0.71	P	P	P	P	P	P	P	P	NM_003550
A_33_P3569068	MAD1L1	672	2182	549	1140	443	1108	431	591	7.92	3.25	5.36	2.08	6.48	2.50	4.09	1.37	1.20	0.55	0.77	P	P	P	P	P	P	P	P	NM_003550
A_21_P0014862		653	918	473	795	423	685	492	520	2.29	1.41	3.81	1.68	3.41	1.62	0.72	1.06	0.96	0.65	1.15	P	P	P	P	P	P	P	P	
A_33_P3403044	MAT2A	652	1073	495	881	510	776	520	590	3.35	1.65	4.24	1.78	2.97	1.52	1.64	1.14	0.85	0.75	0.92	P	P	P	P	P	P	P	P	NM_005911
A_23_P92860	CCNO	641	348	276	148	213	113	231	170	-4.12	0.54	-3.60	0.54	-3.17	0.53	-2.86	0.73	0.99	1.38	0.98	P	P	P	P	P	P	P	P	NM_021147
A_23_P502957	CDH26	626	968	289	523	250	482	204	237	2.93	1.55	3.43	1.81	3.31	1.93	1.39	1.16	1.07	0.60	1.25	P	P	P	P	P	P	P	P	NM_021810
A_32_P115505	LTN1	620	388	365	236	303	157	280	278	-3.16	0.63	-2.53	0.65	-3.28	0.52	-0.07	0.99	0.81	1.91	0.83	P	P	P	P	P	P	P	P	NM_015565
A_21_P0011196		596	1447	408	1095	248	954	405	493	5.97	2.43	7.24	2.68	9.52	3.85	2.54	1.22	1.43	0.32	1.59	P	P	P	P	P	P	P	P	ENST00000550892
A_23_P254888	ZYX	580	1034	673	1072	575	1186	467	552	3.89	1.78	3.42	1.59	5.12	2.06	2.18	1.18	1.29	0.57	1.16	P	P	P	P	P	P	P	P	NM_003461
A_21_P0000925	LINC01341	572	1399	419	1080	384	1024	266	343	6.02	2.45	6.95	2.58	6.92	2.66	2.36	1.29	1.03	0.48	1.09	P	P	P	P	P	P	P	P	AK022825
A_23_P128215	SOCS2	570	283	806	488	1049	539	938	875	-3.17	0.50	-3.67	0.61	-4.68	0.51	-0.89	0.93	0.85	1.81	1.04	P	P	P	P	P	P	P	P	NM_003877
A_24_P313186	CALM1	568	851	717	1308	645	1070	693	738	2.72	1.50	4.41	1.82	3.58	1.66	0.83	1.07	0.91	0.64	1.11	P	P	P	P	P	P	P	P	NM_006888
A_23_P34093	G6PD	567	897	847	2130	740	1958	1006	1338	3.09	1.58	6.77	2.52	6.88	2.65	3.68	1.33	1.05	0.50	1.67	P	P	P	P	P	P	P	P	NM_000402
A_21_P0000924	LINC01341	565	1390	464	1199	409	1018	268	363	6.06	2.46	6.96	2.58	6.44	2.49	2.82	1.36	0.96	0.55	1.01	P	P	P	P	P	P	P	P	AK022825
A_33_P3301445		550	953	508	990	447	762	470	476	3.69	1.73	4.90	1.95	3.78	1.71	0.17	1.01	0.87	0.59	0.98	P	P	P	P	P	P	P	P	
A_21_P0000662	RAB6C-AS1	543	808	700	1175	655	1160	681	704	2.67	1.49	3.80	1.68	4.05	1.77	0.43	1.03	1.06	0.58	1.19	P	P	P	P	P	P	P	P	NR_036537
A_23_P316381	ACOX3	538	754	345	649	251	385	265	282	2.27	1.40	4.64	1.88	2.17	1.53	0.58	1.06	0.81	0.69	1.09	P	P	P	P	P	P	P	P	NM_003501
A_23_P64808	HOXC13	534	1405	450	1150	570	1181	421	538	6.51	2.63	6.89	2.56	5.16	2.07	3.18	1.28	0.81	0.62	0.79	P	P	P	P	P	P	P	P	NM_017410
A_33_P3216890	PAG1	532	942	502	841	357	711	413	501	3.85	1.77	3.78	1.67	4.86	1.99	2.50	1.21	1.19	0.61	1.12	P	P	P	P	P	P	P	P	NM_018440
A_23_P210763	JAG1	525	387	368	230	484	250	306	294	-2.05	0.74	-2.72	0.63	-3.32	0.52	-0.38	0.96	0.83	1.86	0.70	P	P	P	P	P	P	P	P	NM_000214
A_23_P105002	ROM1	517	963	887	2156	679	2924	769	1056	4.19	1.86	6.52	2.43	10.66	4.30	4.09	1.37	1.77	0.32	2.31	P	P	P	P	P	P	P	P	NM_000327
A_33_P3357651	KRTAP10-12	513	817	532	1054	528	806	367	475	3.13	1.59	5.02	1.98	2.99	1.53	2.39	1.29	0.77	0.85	0.96	P	P	P	P	P	P	P	P	NM_198699
A_23_P92727	RAI14	499	726	409	642	520	797	478	622	2.53	1.46	3.32	1.57	3.02	1.53	3.40	1.30	0.97	0.85	1.05	P	P	P	P	P	P	P	P	NM_015577
A_33_P3305790	NOS3	498	397	1519	930	1736	1051	534	561	-1.54	0.80	-3.59	0.61	-3.53	0.61	0.65	1.05	0.99	1.74	0.76	P	P	P	P	P	P	P	P	NM_000603
A_23_P409623	PPFIBP2	464	1182	319	691	281	609	276	321	6.30	2.55	5.67	2.17	5.46	2.16	1.41	1.16	1.00	0.54	0.85	P	P	P	P	P	P	P	P	NM_003621
A_32_P179138	GUCY1A3	461	327	716	396	506	333	548	527	-1.56	0.71	-4.33	0.55	-2.93	0.66	-0.51	0.96	1.19	1.46	0.93	P	P	P	P	P	P	P	P	NM_000856
A_23_P217755	SHROOM2	444	724	243	600	256	628	232	331	3.29	1.63	5.25	2.48	6.34	2.45	3.31	1.43	0.99	0.58	1.50	P	P	P	P	P	P	P	P	NM_001649
A_23_P103756	OVGP1	440	226	413	192	445	205	403	309	-3.02	0.51	-4.41	0.47	-3.89	0.46	-2.45	0.77	0.99	1.67	0.90	P	P	P	P	P	P	P	P	NM_002557
A_23_P143662	PISD	421	649	341	551	392	589	346	459	2.90	1.54	3.51	1.61	2.87	1.50	2.62	1.33	0.93	0.88	0.97	P	P	P	P	P	P	P	P	NM_014338
A_33_P3296862	C16orf89	421	234	1125	636	1806	1008	939	757	-2.66	0.56	-4.17	0.57	-4.10	0.56	-2.78	0.81	0.99	1.44	1.00	P	P	P	P	P	P	P	P	NM_152459
A_23_P8452	LFNG	408	239	383	169	443	282	368	349	-2.42	0.59	-4.75	0.44	-3.16	0.64	-0.48	0.95	1.45	1.49	1.09	P	P	P	P	P	P	P	P	NM_001040167
A_32_P61684	PAG1	408	693	691	1177	557	985	540	760	3.56	1.70	3.90	1.70	4.04	1.77	4.40	1.41	1.04	0.79	1.04	P	P	P	P	P	P	P	P	NM_018440
A_23_P500353	KCNN2	401	4810	388	6010	660	8110	689	3314	16.73	12.00	20.09	15.50	18.32	12.29	20.25	4.81	0.79	0.39	1.02	P	P	P	P	P	P	P	P	NM_021614
A_33_P3314176	FAM46C	351	560	414	658	585	930	446	522	3.14	1.60	3.39	1.59	3.29	1.59	2.01	1.17	1.00	0.73	1.00	P	P	P	P	P	P	P	P	NM_017709
A_32_P19840	LOC100507507	343	976	704	1416	721	2176	435	607	7.04	2.85	5.13	2.01	7.80	3.02	4.30	1.40	1.50	0.46	1.06	P	P	P	P	P	P	P	P	XM_006710194
A_24_P921897	HOOK1	341	1062	282	850	315	966	236	318	7.64	3.11	8.08	3.01	7.91	3.07	2.77	1.35	1.02	0.44	0.98	P	P	P	P	P	P	P	P	NM_015888
A_21_P0000053	C16orf52	331	911	433	1310	556	1667	514	591	6.80	2.75	8.11	3.02	7.76	3.00	1.82	1.15	0.99	0.38	1.09	P	P	P	P	P	P	P	P	NM_001164579
A_23_P55518	SMAD7	329	556	269	468	287	461	271	317	3.52	1.69	3.21	1.74	3.35	1.61	1.47	1.17	0.92	0.73	0.95	P	P	P	P	P	P	P	P	NM_005904
A_21_P0014204	LOC101928915	309	547	674	1053	616	988	1051	1073	3.84	1.77	3.28	1.56	3.34	1.60	0.27	1.02	1.03	0.64	0.91	P	P	P	P	P	P	P	P	XR_242359
A_23_P31073	MYB	302	442	686	1112	737	1176	1129	1202	1.72	1.46	3.54	1.62	3.31	1.60	0.82	1.06	0.99	0.67	1.09	P	P	P	P	P	P	P	P	NM_005375
A_33_P3409844	HOOK1	301	706	177	430	190	461	175	160	5.74	2.35	5.13	2.42	4.47	2.42	-0.85	0.91	1.00	0.38	1.03	P	P	P	P	P	P	P	P	NM_015888
A_24_P67946	NUDT4	292	196	499	237	362	211	350	297	-1.81	0.67	-4.31	0.47	-2.70	0.58	-1.51	0.85	1.23	1.45	0.87	P	P	P	P	P	P	P	P	NM_199040
A_23_P169003	SH2D4A	278	649	259	524	181	350	199	252	5.69	2.33	4.08	2.02	3.35	1.94	2.20	1.27	0.96	0.65	0.83	P	P	P	P	P	P	P	P	NM_022071
A_24_P406060	RNF144B	275	223	462	271	364	202	302	250	-0.96	0.81	-3.10	0.59	-2.95	0.56	-1.75	0.83	0.95	1.49	0.69	P	P	P	P	P	P	P	P	NM_182757
A_23_P369899	TMEM158	275	170	334	173	552	250	369	301	-2.16	0.62	-3.80	0.52	-5.58	0.45	-1.89	0.81	0.87	1.80	0.73	P	P	P	P	P	P	P	P	NM_015444
A_23_P126836	TNFSF4	274	779	355	860	158	357	192	255	7.04	2.85	6.48	2.42	4.13	2.26	2.64	1.33	0.94	0.59	0.79	P	P	P	P	P	P	P	P	NM_003326
A_19_P00317897	LOC101928673	271	504	249	492	346	585	316	414	2.81	1.86	3.94	1.97	3.71	1.69	2.50	1.31	0.86	0.78	0.91	P	P	P	P	P	P	P	P	XR_241149
A_23_P71170	TRPV6	270	1294	398	2149	499	2375	342	570	10.54	4.79	12.37	5.40	11.02	4.76	6.60	1.67	0.88	0.35	0.99	P	P	P	P	P	P	P	P	NM_018646
A_33_P3364854	TRPV6	261	1250	286	1814	399	2066	301	432	10.55	4.79	13.56	6.35	11.61	5.18	3.35	1.44	0.82	0.28	1.08	P	P	P	P	P	P	P	P	NM_018646
A_33_P3250953	SLC35B4	248	529	283	524	248	426	257	323	3.42	2.13	3.56	1.85	2.74	1.72	2.11	1.26	0.93	0.73	0.81	P	P	P	P	P	P	P	P	NM_032826
A_23_P414273	SMIM3	244	428	349	561	288	524	289	352	2.53	1.75	3.49	1.61	4.24	1.82	1.84	1.22	1.13	0.67	1.04	P	P	P	P	P	P	P	P	NM_032947
A_24_P187921	MANEA	243	385	275	414	354	548	244	282	2.08	1.58	2.39	1.51	3.10	1.55	1.34	1.16	1.03	0.75	0.98	P	P	P	P	P	P	P	P	NM_024641
A_33_P3333982	PDE4DIP	241	104	526	323	330	201	371	300	-3.79	0.43	-2.82	0.61	-2.47	0.61	-1.94	0.81	0.99	1.33	1.41	P	P	P	P	P	P	P	P	NM_022359
A_23_P257834	ALB	238	154	323	212	376	230	586	504	-1.98	0.65	-2.42	0.66	-2.47	0.61	-1.96	0.86	0.93	1.40	0.95	P	P	P	P	P	P	P	P	NM_000477
A_23_P131899	SDCBP2	233	126	473	165	2093	960	533	402	-2.79	0.54	-6.11	0.35	-5.70	0.46	-3.63	0.75	1.32	1.65	0.85	P	P	P	P	P	P	P	P	NM_080489
A_23_P19624	BMP6	230	477	176	905	389	1473	155	175	3.31	2.08	9.47	5.13	9.40	3.79	1.14	1.13	0.74	0.30	1.82	P	P	P	P	P	P	P	P	NM_001718
A_33_P3352522	GAS6-AS1	228	172	271	150	468	300	342	340	-1.27	0.75	-3.43	0.55	-3.13	0.64	-0.03	1.00	1.16	1.56	0.85	P	P	P	P	P	P	P	P	AK092862
A_21_P0011335	EFTUD1	223	545	197	425	163	405	158	171	4.04	2.44	4.47	2.16	4.59	2.48	0.76	1.09	1.15	0.44	1.02	P	P	P	P	P	P	P	P	NM_024580
A_21_P0012089	FAR2P1	213	518	278	585	183	382	138	223	4.01	2.43	4.31	2.10	3.73	2.09	4.44	1.62	0.99	0.77	0.86	P	P	P	P	P	P	P	P	NR_026758
A_21_P0012336	GGTLC1	209	282	194	312	212	329	161	219	1.34	1.35	2.75	1.61	2.23	1.55	2.84	1.36	0.97	0.88	1.15	P	P	P	P	P	P	P	P	NM_178311
A_23_P107981	SULT2B1	209	283	189	297	112	196	134	162	1.37	1.35	2.62	1.57	2.82	1.74	1.77	1.21	1.11	0.69	1.29	P	P	P	P	P	P	P	P	NM_004605
A_21_P0010662	LOC100506023	204	371	257	532	204	339	200	272	2.69	1.82	4.21	2.07	2.56	1.66	2.83	1.36	0.80	0.82	0.91	P	P	P	P	P	P	P	P	NR_037845
A_24_P140608	HBEGF	202	427	248	624	154	370	137	177	3.38	2.11	5.36	2.52	4.43	2.40	2.41	1.30	0.95	0.54	1.14	P	P	P	P	P	P	P	P	NM_001945
A_24_P305570	RIN2	189	370	124	206	195	317	137	155	3.04	1.96	2.93	1.66	2.47	1.63	1.13	1.13	0.98	0.69	0.83	P	P	P	P	P	P	P	P	NM_018993
A_23_P53193	SYTL2	181	110	363	239	403	223	245	231	-2.24	0.61	-2.42	0.66	-2.97	0.55	-0.53	0.94	0.84	1.71	0.91	P	P	P	P	P	P	P	P	NM_032943
A_33_P3393088	RAB6C-AS1	176	357	267	464	216	353	193	206	3.21	2.03	3.21	1.74	2.48	1.63	0.62	1.07	0.94	0.65	0.80	P	P	P	P	P	P	P	P	BC044241
A_23_P339053		172	598	157	695	205	800	241	274	5.63	3.48	8.63	4.43	9.61	3.90	1.17	1.13	0.88	0.29	1.12	P	P	P	P	P	P	P	P	ENST00000569656
A_24_P191664	GOLIM4	129	371	124	277	191	383	133	199	4.77	2.88	4.65	2.23	3.52	2.00	3.67	1.49	0.90	0.74	0.70	P	P	P	P	P	P	P	P	NM_014498
A_21_P0014276		118	195	151	265	148	327	167	206	2.25	1.65	3.27	1.76	4.01	2.21	1.96	1.24	1.26	0.56	1.34	P	P	P	P	P	P	P	P	
A_23_P147805	UPP1	118	229	153	284	121	250	105	111	3.00	1.94	3.60	1.86	3.68	2.07	0.21	1.06	1.11	0.51	1.07	P	P	P	P	P	P	P	P	NM_001287428
A_23_P118042	LRRC36	115	232	217	454	246	403	176	207	3.19	2.02	4.28	2.09	2.49	1.64	1.52	1.18	0.78	0.72	0.81	P	P	P	P	P	P	P	P	NM_018296
A_33_P3307955	ABCD3	114	230	231	639	162	353	207	254	3.19	2.02	5.91	2.77	3.94	2.18	1.90	1.23	0.79	0.56	1.08	P	P	P	P	P	P	P	P	NM_001122674

Supplemental Table S3D Specific target genes for 25D3 in siCT cells (69).

ProbeID	GeneSymbol	siCT_V1_signal	siCT_1,25D3_signal	siCT_V2_signal	siCT_25D3_signal	siCYP27B1_V_signal	siCYP27B1_25D3_signal	siCYP27B1_siVDR_V_signal	siCYP27B1_siVDR_25D3_signal	siCT_1,25D3/siCT_V1_Zscore	siCT_1,25D3/siCT_V1_ratio	siCT_25D3/siCT_V2_Zscore	siCT_25D3/siCT_V2_ratio	siCYP27B1_25D3/siCYP27B1_V_Zscore	siCYP27B1_25D3/siCYP27B1_ratio	siCYP27B1_siVDR_25D3/siCYP27B1_siVDR_V_Zscore	siCYP27B1_siVDR_25D3/siCYP27B1_siVDR_V_ratio	siCYP27B1_25/V/siCT_25D3/V2_ratio	siCYP27B1_siVDR_25D3/V/siCYP27B1_25D3/V_ratio	siC27B1_25D3/V/siCT_1,25D3/V1_ratio	siCT_V1_flag	siCT_1,25D3_flag	siCT_V2_flag	siCT_25D3_flag	siCYP27B1_V_flag	siCYP27B1_25D3_flag	siCYP27B1_siVDR_V_flag	siCYP27B1_siVDR_25D3_flag	PrimaryAccession
A_21_P0011866	RPS2	180193	185340	133545	213992	140491	149477	136172	141974	0.69	1.03	6.81	1.60	0.90	1.06	1.02	1.04	0.66	0.98	1.03	P	P	P	P	P	P	P	P	NM_002952
A_23_P106844	MT2A	27944	7668	29692	14177	30083	25807	33511	24943	-11.66	0.27	-7.40	0.48	-1.81	0.86	-3.83	0.74	1.80	0.87	3.13	P	P	P	P	P	P	P	P	NM_005953
A_33_P3233645	MT1G	10806	2088	6493	2983	9896	7785	8926	6027	-11.83	0.19	-5.96	0.46	-2.27	0.79	-2.16	0.68	1.71	0.86	4.07	P	P	P	P	P	P	P	P	NM_005950
A_33_P3220837	MAFB	9972	16102	14410	22495	22148	32617	16495	17917	4.32	1.61	4.48	1.56	4.53	1.47	0.48	1.09	0.94	0.74	0.91	P	P	P	P	P	P	P	P	NM_005461
A_23_P427703	MT1L	8823	1995	5265	2586	6471	5358	6418	4653	-10.70	0.23	-5.45	0.49	-1.78	0.83	-5.14	0.73	1.69	0.88	3.66	P	P	P	P	P	P	P	P	NR_001447
A_23_P37983	MT1B	8565	2176	6045	2895	7709	6365	7465	5245	-9.86	0.25	-5.64	0.48	-1.81	0.83	-1.94	0.70	1.72	0.85	3.25	P	P	P	P	P	P	P	P	NM_005947
A_33_P3315314	MT1HL1	7062	2098	5552	2867	7058	5971	6508	4742	-8.73	0.30	-5.07	0.52	-1.58	0.85	-5.06	0.73	1.64	0.86	2.85	P	P	P	P	P	P	P	P	NM_001276687
A_23_P206724	MT1E	7058	1617	4099	1958	5511	4716	4990	3578	-10.60	0.23	-5.66	0.48	-1.14	0.86	-5.31	0.72	1.79	0.84	3.73	P	P	P	P	P	P	P	P	NM_175617
A_33_P3350056	MT1X	5959	2087	3706	2150	4575	4111	4709	3490	-7.55	0.35	-4.18	0.58	-0.79	0.90	-4.79	0.74	1.55	0.82	2.57	P	P	P	P	P	P	P	P	NM_005952
A_23_P401568	MAT2A	4795	7941	4686	8200	5471	8029	5492	5608	4.55	1.66	5.63	1.75	3.68	1.47	0.33	1.02	0.84	0.70	0.89	P	P	P	P	P	P	P	P	NM_005911
A_33_P3288364	SPATA2L	4122	6216	3066	4634	2556	3735	2749	2932	2.96	1.51	3.16	1.51	2.77	1.46	1.03	1.07	0.97	0.73	0.97	P	P	P	P	P	P	P	P	NM_152339
A_33_P3358158	CRYBB2P1	3632	3431	2138	3223	5398	3912	3767	3299	-0.41	0.94	3.14	1.51	-2.36	0.72	-2.12	0.88	0.48	1.21	0.77	P	P	P	P	P	P	P	P	NR_033733
A_23_P17345	MAFB	3379	4913	4330	6691	6739	8847	4732	5098	2.70	1.45	3.33	1.55	2.61	1.31	1.18	1.08	0.85	0.82	0.90	P	P	P	P	P	P	P	P	NM_005461
A_32_P5276	ARHGEF26	3294	4835	3584	5432	4971	7387	5386	5969	2.76	1.47	3.18	1.52	3.79	1.49	1.64	1.11	0.98	0.75	1.01	P	P	P	P	P	P	P	P	NM_001251962
A_23_P255663	MANEA	2352	4124	2158	3440	3824	5352	2248	2775	4.04	1.75	3.57	1.59	2.45	1.40	3.36	1.23	0.88	0.88	0.80	P	P	P	P	P	P	P	P	NM_024641
A_23_P428129	CDKN1C	2300	1635	1618	1028	3361	2251	1712	1396	-2.45	0.71	-3.32	0.64	-2.93	0.67	-2.62	0.82	1.05	1.22	0.94	P	P	P	P	P	P	P	P	NM_000076
A_23_P110212	ACSL1	2142	4416	1343	2444	1589	2355	1861	2152	5.21	2.06	4.58	1.82	2.87	1.48	2.31	1.16	0.81	0.78	0.72	P	P	P	P	P	P	P	P	NM_001995
A_24_P943393	AHNAK	1812	1166	2549	1603	3016	2339	2372	2885	-2.98	0.64	-3.56	0.63	-1.86	0.78	3.12	1.22	1.23	1.57	1.21	P	P	P	P	P	P	P	P	NM_001620
A_32_P47107		1603	2744	4604	7632	6179	9008	3421	4141	3.87	1.71	3.87	1.66	3.61	1.46	3.05	1.21	0.88	0.83	0.85	P	P	P	P	P	P	P	P	ENST00000561761
A_24_P161973	ATP11A	1546	2269	1107	1697	1139	1683	986	1124	2.76	1.47	3.14	1.53	2.85	1.48	1.70	1.14	0.96	0.77	1.01	P	P	P	P	P	P	P	P	NM_015205
A_19_P00809119	CASC15	1191	782	595	370	1352	1189	589	1025	-2.84	0.66	-3.48	0.62	-0.89	0.88	7.16	1.74	1.41	1.98	1.34	P	P	P	P	P	P	P	P	NR_015410
A_23_P133470	PJA2	1128	2049	866	1548	581	810	746	850	4.02	1.82	4.26	1.79	2.35	1.39	1.69	1.14	0.78	0.82	0.77	P	P	P	P	P	P	P	P	NM_014819
A_23_P4494	DSC2	1013	730	2427	1415	1559	1046	1461	1392	-2.21	0.72	-4.14	0.58	-2.80	0.67	-0.62	0.95	1.15	1.42	0.93	P	P	P	P	P	P	P	P	NM_024422
A_23_P80048	FER1L4	980	1409	712	1127	963	1032	700	751	2.44	1.44	3.37	1.58	0.50	1.07	0.92	1.07	0.68	1.00	0.75	P	P	P	P	P	P	P	P	NR_119376
A_32_P126311	SLC38A9	855	1482	1194	1797	1427	1924	1664	1753	3.70	1.73	3.00	1.51	2.18	1.35	0.83	1.05	0.90	0.78	0.78	P	P	P	P	P	P	P	P	NM_173514
A_23_P319423	KCNK5	770	583	1008	614	666	483	724	574	-1.88	0.76	-3.63	0.61	-2.26	0.72	-2.99	0.79	1.19	1.09	0.96	P	P	P	P	P	P	P	P	NM_003740
A_23_P345460	PLEKHG4	758	913	653	1004	779	1155	597	649	1.24	1.20	3.16	1.54	2.79	1.48	1.08	1.09	0.96	0.73	1.23	P	P	P	P	P	P	P	P	NM_015432
A_33_P3302957	PLEKHG4	742	978	448	679	562	726	427	410	1.86	1.32	3.06	1.52	1.82	1.29	-0.37	0.96	0.85	0.74	0.98	P	P	P	P	P	P	P	P	NM_015432
A_23_P217319	FGF13	675	448	1728	1081	1816	1340	1336	1259	-2.76	0.66	-3.44	0.63	-2.23	0.74	-0.75	0.94	1.18	1.28	1.11	P	P	P	P	P	P	P	P	NM_004114
A_32_P117170	NAPEPLD	597	1184	981	1645	1557	2072	1196	1391	4.61	1.98	3.80	1.68	2.09	1.33	1.96	1.16	0.79	0.87	0.67	P	P	P	P	P	P	P	P	NM_001122838
A_33_P3627001	PEBP4	595	763	198	301	364	443	240	257	1.68	1.28	2.42	1.52	1.40	1.22	0.62	1.07	0.80	0.88	0.95	P	P	P	P	P	P	P	P	NM_144962
A_24_P38347	DPYSL2	577	388	544	345	700	511	706	618	-2.67	0.67	-3.33	0.63	-2.21	0.73	-1.70	0.88	1.15	1.20	1.08	P	P	P	P	P	P	P	P	NM_001386
A_32_P141724	COMMD7	531	658	639	1072	568	704	573	626	1.44	1.24	3.80	1.68	1.53	1.24	1.14	1.09	0.74	0.88	1.00	P	P	P	P	P	P	P	P	NM_053041
A_23_P145529	PKIB	517	832	649	1085	853	1172	553	558	3.20	1.61	3.77	1.67	2.25	1.37	0.12	1.01	0.82	0.73	0.85	P	P	P	P	P	P	P	P	NM_181795
A_33_P3231156		499	149	179	107	215	205	235	186	-5.48	0.30	-2.96	0.60	-0.24	0.95	-2.16	0.79	1.59	0.83	3.20	P	P	P	P	P	P	P	P	
A_23_P23815	SLC30A1	489	228	501	305	614	609	529	471	-3.44	0.47	-2.87	0.61	-0.04	0.99	-1.49	0.89	1.63	0.90	2.12	P	P	P	P	P	P	P	P	NM_021194
A_23_P161190	VIM	475	324	461	289	454	354	305	301	-1.72	0.68	-2.69	0.63	-1.74	0.78	-0.11	0.99	1.24	1.27	1.14	P	P	P	P	P	P	P	P	NM_003380
A_33_P3230244	CSMD1	467	843	678	1089	626	885	502	568	3.98	1.81	3.48	1.61	2.45	1.41	1.60	1.13	0.88	0.80	0.78	P	P	P	P	P	P	P	P	NM_033225
A_23_P105562	VWF	445	915	555	1012	313	417	457	507	4.85	2.06	4.40	1.82	2.03	1.33	1.35	1.11	0.73	0.83	0.65	P	P	P	P	P	P	P	P	NM_000552
A_23_P161424	PLXDC2	424	344	386	254	618	437	407	422	-0.94	0.81	-2.43	0.66	-2.42	0.71	0.33	1.04	1.08	1.46	0.87	P	P	P	P	P	P	P	P	NM_032812
A_33_P3369153	KIF3C	421	415	428	278	398	301	408	403	-0.11	0.99	-2.49	0.65	-1.40	0.76	-0.12	0.99	1.16	1.31	0.77	P	P	P	P	P	P	P	P	NM_002254
A_33_P3214665	MAP2	370	269	424	257	838	701	319	313	-1.44	0.73	-2.89	0.61	-1.24	0.84	-0.17	0.98	1.38	1.17	1.15	P	P	P	P	P	P	P	P	NM_002374
A_23_P57760	PXYLP1	345	174	383	240	467	363	359	350	-3.10	0.50	-2.70	0.63	-1.75	0.78	-0.24	0.97	1.24	1.25	1.54	P	P	P	P	P	P	P	P	NM_152282
A_23_P57118	TGM3	339	204	284	166	173	118	266	225	-2.30	0.60	-3.12	0.58	-1.92	0.68	-1.55	0.84	1.17	1.24	1.13	P	P	P	P	P	P	P	P	NM_003245
A_33_P3225268	QSOX1	337	457	406	629	410	576	355	342	1.38	1.36	3.21	1.55	2.41	1.41	-0.35	0.96	0.91	0.68	1.03	P	P	P	P	P	P	P	P	NM_001004128
A_21_P0001768	LOC200772	336	397	243	365	369	470	330	351	0.75	1.18	2.36	1.50	1.71	1.27	0.58	1.06	0.85	0.84	1.08	P	P	P	P	P	P	P	P	NR_033841
A_23_P411102	DLG4	320	332	198	312	182	204	151	131	0.17	1.04	2.65	1.58	0.58	1.12	-1.32	0.87	0.71	0.77	1.08	P	P	P	P	P	P	P	P	NM_001365
A_21_P0000847	LOC100630918	303	352	312	551	377	520	383	407	0.67	1.16	3.30	1.77	2.28	1.38	0.56	1.06	0.78	0.77	1.19	P	P	P	P	P	P	P	P	NR_038942
A_24_P317907	SORBS1	299	199	235	152	199	174	155	152	-1.84	0.67	-2.53	0.65	-0.67	0.87	-0.15	0.98	1.35	1.13	1.31	P	P	P	P	P	P	P	P	NM_001034954
A_33_P3409154	SLC22A23	250	390	405	631	364	484	287	357	2.00	1.56	3.26	1.56	2.03	1.33	2.01	1.24	0.85	0.93	0.85	P	P	P	P	P	P	P	P	NM_015482
A_23_P218807	ZC3H7B	248	235	196	336	237	192	215	160	-0.25	0.95	3.12	1.71	-1.06	0.81	-2.72	0.74	0.47	0.92	0.85	P	P	P	P	P	P	P	P	NM_017590
A_33_P3628409	PKI55	246	206	223	145	173	152	156	207	-0.79	0.84	-2.49	0.65	-0.63	0.88	2.62	1.33	1.35	1.51	1.05	P	P	P	P	P	P	P	P	NR_037701
A_21_P0000651	HERC2P7	240	409	242	387	370	521	332	352	2.42	1.71	2.73	1.60	2.42	1.41	0.57	1.06	0.88	0.75	0.82	P	P	P	P	P	P	P	P	NR_036470
A_24_P69095	ENC1	221	172	539	353	576	384	404	364	-1.14	0.78	-3.09	0.66	-2.86	0.67	-0.95	0.90	1.01	1.35	0.86	P	P	P	P	P	P	P	P	NM_003633
A_33_P3349395	CECR2	218	205	233	365	695	512	447	401	-0.26	0.94	2.61	1.57	-2.14	0.74	-1.39	0.90	0.47	1.22	0.78	P	P	P	P	P	P	P	P	XM_006724079
A_23_P58390	C4orf32	208	415	479	760	479	644	447	528	3.12	2.00	3.39	1.59	2.10	1.34	2.14	1.18	0.85	0.88	0.67	P	P	P	P	P	P	P	P	NM_152400
A_33_P3368800	NBPF12	197	362	154	240	197	251	133	227	2.75	1.84	2.58	1.56	1.24	1.28	4.94	1.71	0.82	1.34	0.69	P	P	P	P	P	P	P	P	NM_001278141
A_23_P213085	SLC10A7	193	413	161	245	124	159	106	137	3.45	2.15	2.46	1.53	1.28	1.29	2.37	1.29	0.84	1.01	0.60	P	P	P	P	P	P	P	P	NM_001029998
A_23_P119778	SLC39A10	189	540	174	283	163	181	163	180	4.75	2.86	2.80	1.62	0.53	1.11	0.88	1.10	0.68	0.99	0.39	P	P	P	P	P	P	P	P	NM_020342
A_24_P398147	NEBL	178	108	320	204	285	264	213	215	-2.28	0.60	-2.59	0.64	-0.37	0.93	0.08	1.01	1.45	1.09	1.53	P	P	P	P	P	P	P	P	NM_006393
A_33_P3352307	RPS6KA1	176	219	225	361	205	293	205	222	1.00	1.25	2.75	1.61	1.80	1.43	0.72	1.08	0.89	0.76	1.14	P	P	P	P	P	P	P	P	NM_002953
A_23_P97990	HTRA1	160	172	309	478	1043	1478	422	471	0.34	1.08	2.53	1.55	2.47	1.42	1.42	1.12	0.92	0.79	1.32	P	P	P	P	P	P	P	P	NM_002775
A_33_P3503937	LOC284581	154	185	167	431	544	397	394	393	0.84	1.20	5.50	2.58	-2.22	0.73	-0.02	1.00	0.28	1.37	0.61	P	P	P	P	P	P	P	P	AK094426
A_23_P5018	CEBPA-AS1	140	148	274	165	204	226	181	211	0.26	1.06	-2.94	0.60	0.54	1.11	1.43	1.17	1.85	1.05	1.05	P	P	P	P	P	P	P	P	NR_026887
A_19_P00320295	IPW	130	161	118	197	138	187	204	218	0.95	1.23	2.96	1.67	1.57	1.36	0.62	1.07	0.82	0.78	1.10	P	P	P	P	P	P	P	P	NR_023915
A_24_P349117	GPR158	123	207	232	376	214	250	269	268	2.36	1.69	2.80	1.62	0.79	1.17	-0.01	1.00	0.72	0.85	0.69	P	P	P	P	P	P	P	P	NM_020752
A_23_P57199	GGTLC1	119	161	136	205	167	242	115	132	1.38	1.36	2.37	1.51	1.88	1.45	1.29	1.15	0.96	0.79	1.07	P	P	P	P	P	P	P	P	NM_178311
A_23_P200874	CEP85	119	127	132	216	109	141	128	139	0.30	1.07	2.88	1.64	1.32	1.30	0.75	1.08	0.79	0.84	1.21	P	P	P	P	P	P	P	P	NM_022778
A_19_P00319646	LINC00673	102	192	154	258	213	269	149	184	2.85	1.88	2.99	1.67	1.18	1.26	1.93	1.23	0.75	0.98	0.67	P	P	P	P	P	P	P	P	NR_036488

Supplemental Table S3E Specific target genes for 25D3 in siCYP27B1 cells (58).

ProbeID	GeneSymbol	siCT_V1_signal	siCT_1,25D3_signal	siCT_V2_signal	siCT_25D3_signal	siCYP27B1_V_signal	siCYP27B1_25D3_signal	siCYP27B1_siVDR_V_signal	siCYP27B1_siVDR_25D3_signal	siCT_1,25D3/siCT_V1_Zscore	siCT_1,25D3/siCT_V1_ratio	siCT_25D3/siCT_V2_Zscore	siCT_25D3/siCT_V2_ratio	siCYP27B1_25D3/siCYP27B1_V_Zscore	siCYP27B1_25D3/siCYP27B1_ratio	siCYP27B1_siVDR_25D3/siCYP27B1_siVDR_V_Zscore	siCYP27B1_siVDR_25D3/siCYP27B1_siVDR_V_ratio	siCYP27B1_25/V/siCT_25D3/V2_ratio	siCYP27B1_siVDR_25D3/V/siCYP27B1_25D3/V_ratio	siC27B1_25D3/V/siCT_1,25D3/V1_ratio	siCT_V1_flag	siCT_1,25D3_flag	siCT_V2_flag	siCT_25D3_flag	siCYP27B1_V_flag	siCYP27B1_25D3_flag	siCYP27B1_siVDR_V_flag	siCYP27B1_siVDR_25D3_flag	PrimaryAccession
A_33_P3233906	RAMP1	15283	19548	13083	17148	11417	17169	10344	11543	2.22	1.28	2.73	1.31	3.91	1.50	0.63	1.12	1.15	0.74	1.18	P	P	P	P	P	P	P	P	NM_005855
A_23_P58407	UGT2B15	15231	11268	14398	12141	15066	9545	16289	14573	-2.71	0.74	-1.69	0.84	-4.33	0.63	-0.60	0.89	0.75	1.41	0.86	P	P	P	P	P	P	P	P	NM_001076
A_21_P0012591	UGT2B11	11142	5801	5880	4425	5956	3639	6228	5752	-5.88	0.52	-2.18	0.75	-3.60	0.61	-1.28	0.92	0.81	1.51	1.17	P	P	P	P	P	P	P	P	NM_001073
A_23_P7342	UGT2B10	10174	5702	5738	4724	5874	3842	6199	5511	-5.22	0.56	-1.49	0.82	-3.11	0.65	-1.88	0.89	0.79	1.36	1.17	P	P	P	P	P	P	P	P	NM_001075
A_23_P104318	DDIT4	6656	9415	6803	9868	6291	10166	6293	7137	3.13	1.41	3.75	1.45	4.59	1.62	0.72	1.13	1.11	0.70	1.14	P	P	P	P	P	P	P	P	NM_019058
A_23_P25974	TTC7B	2009	2871	2743	4022	2626	4278	2846	3086	2.57	1.43	2.93	1.47	3.56	1.63	1.29	1.08	1.11	0.67	1.14	P	P	P	P	P	P	P	P	NM_001010854
A_23_P129413	DPEP3	1323	1189	421	402	251	387	283	300	-0.73	0.90	-0.26	0.96	2.19	1.54	0.52	1.06	1.61	0.69	1.71	P	P	P	P	P	P	P	P	NM_022357
A_23_P143143	ID2	1139	955	587	452	678	417	546	472	-1.20	0.84	-1.92	0.77	-3.42	0.61	-1.86	0.87	0.80	1.41	0.73	P	P	P	P	P	P	P	P	NM_002166
A_24_P55496	OSR2	1033	1711	561	790	415	624	365	428	3.39	1.66	2.51	1.41	2.89	1.50	1.49	1.17	1.07	0.78	0.91	P	P	P	P	P	P	P	P	NM_053001
A_23_P210015	PTPN18	955	1165	953	1327	817	1341	815	918	1.33	1.22	2.43	1.39	3.51	1.64	1.55	1.13	1.18	0.69	1.34	P	P	P	P	P	P	P	P	NM_014369
A_23_P80382	PRR5	941	1129	754	944	583	894	668	649	1.22	1.20	1.65	1.25	3.03	1.53	-0.37	0.97	1.22	0.63	1.28	P	P	P	P	P	P	P	P	NM_015366
A_23_P250164	HGD	916	811	198	139	253	160	289	279	-0.83	0.88	-2.05	0.70	-2.30	0.63	-0.32	0.97	0.90	1.53	0.71	P	P	P	P	P	P	P	P	NM_000187
A_24_P38951	RELT	894	1087	583	797	464	732	586	627	1.31	1.22	2.30	1.37	3.22	1.58	0.88	1.07	1.15	0.68	1.30	P	P	P	P	P	P	P	P	NM_032871
A_21_P0014711	EIF4E2	851	1198	700	836	805	1238	689	729	2.30	1.41	1.31	1.19	3.05	1.54	0.74	1.06	1.29	0.69	1.09	P	P	P	P	P	P	P	P	NM_001282958
A_23_P46470	ERRFI1	790	868	921	1097	947	1452	1013	1018	0.63	1.10	1.29	1.19	3.03	1.53	0.07	1.00	1.29	0.65	1.40	P	P	P	P	P	P	P	P	NM_018948
A_33_P3385870	CCNG2	764	525	842	581	900	578	931	805	-2.53	0.69	-2.72	0.69	-3.11	0.64	-1.86	0.87	0.93	1.35	0.93	P	P	P	P	P	P	P	P	NM_004354
A_23_P33791	SSBP2	734	450	998	758	1076	702	930	951	-3.29	0.61	-2.01	0.76	-3.00	0.65	0.29	1.02	0.86	1.57	1.06	P	P	P	P	P	P	P	P	NM_012446
A_33_P3369969	ATXN1L	700	1008	868	1133	621	969	758	840	2.45	1.44	1.96	1.31	3.15	1.56	1.32	1.11	1.20	0.71	1.08	P	P	P	P	P	P	P	P	NM_001137675
A_21_P0014553		652	997	530	761	633	972	760	772	2.85	1.53	2.66	1.44	3.04	1.54	0.20	1.02	1.07	0.66	1.00	P	P	P	P	P	P	P	P	ENST00000548057
A_33_P3376971	CHAC1	587	558	586	586	556	923	678	727	-0.35	0.95	0.01	1.00	3.59	1.66	0.90	1.07	1.66	0.65	1.75	P	P	P	P	P	P	P	P	NM_024111
A_23_P500381	HTR7	547	783	510	717	497	746	502	543	2.40	1.43	2.50	1.41	2.89	1.50	1.03	1.08	1.07	0.72	1.05	P	P	P	P	P	P	P	P	NM_019859
A_23_P152406	CAPNS2	540	858	490	702	297	471	346	403	3.11	1.59	2.63	1.43	3.26	1.58	1.43	1.17	1.11	0.74	1.00	P	P	P	P	P	P	P	P	NM_032330
A_23_P21495	FCGBP	516	545	631	504	1775	1094	657	859	0.36	1.06	-1.65	0.80	-3.54	0.62	3.46	1.31	0.77	2.12	0.58	P	P	P	P	P	P	P	P	NM_003890
A_32_P174572	HTR7P1	505	771	547	738	507	825	566	595	2.84	1.53	2.20	1.35	3.44	1.63	0.65	1.05	1.20	0.65	1.06	P	P	P	P	P	P	P	P	NR_002774
A_21_P0002852	SEMA3B-AS1	480	499	486	718	346	538	234	252	0.25	1.04	2.86	1.48	3.13	1.56	0.69	1.08	1.05	0.69	1.50	P	P	P	P	P	P	P	P	NR_110702
A_24_P322395	EGLN2	465	592	651	952	545	842	509	601	1.62	1.27	2.79	1.46	3.08	1.54	2.15	1.18	1.06	0.76	1.21	P	P	P	P	P	P	P	P	NM_080732
A_32_P133840	TMCC2	430	368	197	197	179	103	147	166	-0.70	0.86	0.00	1.00	-2.79	0.57	1.08	1.12	0.57	1.96	0.67	P	P	P	P	P	P	P	P	NM_014858
A_23_P137470	SIPA1L2	410	339	498	337	902	507	515	444	-0.86	0.83	-2.25	0.68	-4.05	0.56	-1.93	0.86	0.83	1.53	0.68	P	P	P	P	P	P	P	P	NM_020808
A_24_P350228	SLC22A23	403	414	291	340	189	302	214	263	0.17	1.03	0.91	1.17	2.39	1.60	1.90	1.23	1.37	0.77	1.56	P	P	P	P	P	P	P	P	NM_021945
A_23_P418234	PHLPP2	393	590	425	607	407	618	441	560	2.73	1.50	2.61	1.43	2.95	1.52	3.08	1.27	1.06	0.84	1.01	P	P	P	P	P	P	P	P	NM_015020
A_23_P55998	SLC1A5	361	461	470	634	373	570	535	594	1.63	1.28	2.19	1.35	3.01	1.53	1.35	1.11	1.14	0.73	1.20	P	P	P	P	P	P	P	P	NM_005628
A_21_P0013145		340	459	210	313	213	359	220	213	1.36	1.35	2.31	1.49	2.65	1.69	-0.31	0.97	1.13	0.57	1.25	P	P	P	P	P	P	P	P	ENST00000428896
A_24_P225616	RRM2	338	258	399	337	302	182	739	633	-1.23	0.76	-0.97	0.85	-2.55	0.60	-2.00	0.86	0.71	1.42	0.79	P	P	P	P	P	P	P	P	NM_001034
A_33_P3393091		328	476	334	492	262	445	248	248	1.68	1.45	2.24	1.47	2.68	1.70	-0.01	1.00	1.15	0.59	1.17	P	P	P	P	P	P	P	P	DA197111
A_32_P167471	CLMN	315	486	345	427	276	453	274	314	1.96	1.54	1.24	1.24	3.52	1.64	1.26	1.15	1.33	0.70	1.07	P	P	P	P	P	P	P	P	NM_024734
A_32_P69368	ID2	314	238	210	179	259	163	207	190	-1.25	0.76	-0.93	0.85	-2.31	0.63	-0.77	0.92	0.74	1.46	0.83	P	P	P	P	P	P	P	P	NM_002166
A_33_P3282489	GCNT1	312	258	623	513	680	438	753	720	-0.87	0.83	-1.42	0.82	-3.09	0.64	-0.57	0.96	0.78	1.49	0.78	P	P	P	P	P	P	P	P	NM_001097634
A_24_P366777	NOTCH2NL	311	603	249	364	290	489	227	341	4.44	1.94	2.20	1.46	3.70	1.69	3.76	1.50	1.16	0.89	0.87	P	P	P	P	P	P	P	P	ENST00000344859
A_33_P3269109	GUCY1A3	280	196	299	203	304	186	372	327	-1.62	0.70	-2.24	0.68	-2.47	0.61	-1.19	0.88	0.90	1.44	0.87	P	P	P	P	P	P	P	P	ENST00000512983
A_23_P40956	GHRL	276	195	334	241	485	305	369	360	-1.57	0.71	-1.88	0.72	-3.27	0.63	-0.22	0.98	0.87	1.55	0.89	P	P	P	P	P	P	P	P	NM_016362
A_33_P3246985	PDE4DIP	262	120	312	214	267	172	366	340	-3.53	0.46	-2.18	0.69	-2.20	0.64	-0.67	0.93	0.94	1.44	1.40	P	P	P	P	P	P	P	P	NM_022359
A_33_P3358228	VWA2	257	200	147	130	277	162	189	237	-1.13	0.78	-0.72	0.88	-2.68	0.59	2.08	1.25	0.66	2.14	0.75	P	P	P	P	P	P	P	P	ENST00000603594
A_33_P3398331	MMP24	251	409	304	454	354	556	288	331	2.19	1.63	2.33	1.49	3.20	1.57	1.29	1.15	1.05	0.73	0.97	P	P	P	P	P	P	P	P	NM_006690
A_23_P210210	EPAS1	241	210	384	272	561	348	363	293	-0.62	0.87	-1.99	0.71	-3.35	0.62	-1.98	0.81	0.88	1.30	0.71	P	P	P	P	P	P	P	P	NM_001430
A_23_P410587	JADE1	239	203	319	258	324	212	335	298	-0.75	0.85	-1.23	0.81	-2.12	0.65	-1.06	0.89	0.81	1.36	0.77	P	P	P	P	P	P	P	P	NM_024900
A_33_P3215906	NBPF9	227	431	203	302	250	376	196	281	2.89	1.90	2.31	1.49	2.08	1.51	3.30	1.43	1.01	0.95	0.80	P	P	P	P	P	P	P	P	NM_001037675
A_33_P3334443	FAM69A	214	327	391	541	382	690	314	397	1.90	1.52	2.37	1.38	4.19	1.81	2.19	1.27	1.31	0.70	1.19	P	P	P	P	P	P	P	P	NM_001252271
A_21_P0002904		207	233	420	391	429	281	541	595	0.54	1.13	-0.41	0.93	-2.13	0.65	1.23	1.10	0.70	1.68	0.58	P	P	P	P	P	P	P	P	ENST00000495159
A_23_P170901	PACRG	199	122	296	218	202	131	361	374	-2.23	0.61	-1.75	0.74	-2.15	0.65	0.34	1.04	0.88	1.59	1.06	P	P	P	P	P	P	P	P	NM_152410
A_32_P172141	CDON	196	332	197	294	191	352	184	233	2.39	1.70	2.33	1.49	3.09	1.84	2.16	1.26	1.23	0.69	1.09	P	P	P	P	P	P	P	P	NM_016952
A_23_P152428	MARVELD3	194	301	173	259	173	286	164	194	1.97	1.55	2.34	1.50	2.56	1.66	1.55	1.18	1.11	0.71	1.07	P	P	P	P	P	P	P	P	NM_052858
A_21_P0014313		170	109	162	145	199	121	210	211	-2.00	0.64	-0.62	0.90	-2.50	0.61	0.05	1.00	0.68	1.66	0.94	P	P	P	P	P	P	P	P	ENST00000573315
A_23_P125596	RPS6KA6	162	289	183	254	108	170	151	165	2.60	1.78	1.91	1.39	2.30	1.57	0.80	1.09	1.13	0.69	0.89	P	P	P	P	P	P	P	P	NM_014496
A_24_P879740	MAP1B	158	142	535	383	414	264	452	491	-0.50	0.90	-2.44	0.72	-2.25	0.64	1.09	1.09	0.89	1.70	0.71	P	P	P	P	P	P	P	P	NM_005909
A_23_P35684	INPP5F	154	158	202	175	164	247	178	167	0.12	1.03	-0.81	0.87	2.07	1.50	-0.56	0.94	1.73	0.63	1.46	P	P	P	P	P	P	P	P	NM_014937
A_33_P3540143	IL17RA	120	161	138	184	128	202	182	196	1.35	1.35	1.69	1.34	2.30	1.57	0.69	1.08	1.18	0.68	1.17	P	P	P	P	P	P	P	P	NM_014339
A_33_P3719214		108	113	162	153	124	189	132	119	0.18	1.04	-0.33	0.94	2.16	1.53	-0.89	0.91	1.62	0.59	1.47	P	P	P	P	P	P	P	P	ENST00000456627
A_24_P497186	IRF2BP2	108	103	175	150	238	153	185	168	-0.20	0.96	-0.89	0.86	-2.21	0.64	-0.87	0.91	0.75	1.41	0.67	P	P	P	P	P	P	P	P	NM_182972

Supplemental Table S4 Gene list of 25D3 target genes in siCYP27B1 cells.

Supplemental Table S4A 25D3 target genes mediated by VDR (176).

ProbeID	GeneSymbol	siCT_V1_signal	siCT_1,25D3_signal	siCT_V2_signal	siCT_25D3_signal	siCYP27B1_V_signal	siCYP27B1_25D3_signal	siCYP27B1_siVDR_V_signal	siCYP27B1_siVDR_25D3_signal	siCT_1,25D3/siCT_V1_Zscore	siCT_1,25D3/siCT_V1_ratio	siCT_25D3/siCT_V2_Zscore	siCT_25D3/siCT_V2_ratio	siCYP27B1_25D3/siCYP27B1_V_Zscore	siCYP27B1_25D3/siCYP27B1_ratio	siCYP27B1_siVDR_25D3/siCYP27B1_siVDR_V_Zscore	siCYP27B1_siVDR_25D3/siCYP27B1_siVDR_V_ratio	siCYP27B1_25/V/siCT_25D3/V2_ratio	siCYP27B1_siVDR_25D3/V/siCYP27B1_25D3/V_ratio	siC27B1_25D3/V/siCT_1,25D3/V1_ratio	siCT_V1_flag	siCT_1,25D3_flag	siCT_V2_flag	siCT_25D3_flag	siCYP27B1_V_flag	siCYP27B1_25D3_flag	siCYP27B1_siVDR_V_flag	siCYP27B1_siVDR_25D3_flag	PrimaryAccession
A_33_P3285545	CLDN4	31397	48717	50313	80442	38373	64904	38181	45207	5.47	1.55	5.94	1.60	6.16	1.69	2.15	1.18	1.06	0.70	1.09	P	P	P	P	P	P	P	P	NM_001305
A_33_P3233906	RAMP1	15283	19548	13083	17148	11417	17169	10344	11543	2.22	1.28	2.73	1.31	3.91	1.50	0.63	1.12	1.15	0.74	1.18	P	P	P	P	P	P	P	P	NM_005855
A_23_P58407	UGT2B15	15231	11268	14398	12141	15066	9545	16289	14573	-2.71	0.74	-1.69	0.84	-4.33	0.63	-0.60	0.89	0.75	1.41	0.86	P	P	P	P	P	P	P	P	NM_001076
A_24_P208345	SLC45A3	11280	16965	11201	20087	9303	17392	7790	8784	3.68	1.50	5.87	1.79	5.98	1.87	0.69	1.13	1.04	0.60	1.24	P	P	P	P	P	P	P	P	NM_033102
A_21_P0012591	UGT2B11	11142	5801	5880	4425	5956	3639	6228	5752	-5.88	0.52	-2.18	0.75	-3.60	0.61	-1.28	0.92	0.81	1.51	1.17	P	P	P	P	P	P	P	P	NM_001073
A_23_P7342	UGT2B10	10174	5702	5738	4724	5874	3842	6199	5511	-5.22	0.56	-1.49	0.82	-3.11	0.65	-1.88	0.89	0.79	1.36	1.17	P	P	P	P	P	P	P	P	NM_001075
A_23_P113111	AR	7199	19386	7131	19811	7680	22300	8545	12200	8.93	2.69	10.26	2.78	10.18	2.90	2.00	1.43	1.05	0.49	1.08	P	P	P	P	P	P	P	P	NM_000044
A_23_P104318	DDIT4	6656	9415	6803	9868	6291	10166	6293	7137	3.13	1.41	3.75	1.45	4.59	1.62	0.72	1.13	1.11	0.70	1.14	P	P	P	P	P	P	P	P	NM_019058
A_23_P69573	GUCY1A3	6022	4366	9759	5702	8708	5006	8498	8051	-2.31	0.73	-5.37	0.58	-5.26	0.57	-0.28	0.95	0.98	1.65	0.79	P	P	P	P	P	P	P	P	NM_000856
A_23_P382188	STAP2	4876	7721	4088	7511	5905	9139	4463	4569	4.15	1.58	4.65	1.84	4.18	1.55	0.37	1.02	0.84	0.66	0.98	P	P	P	P	P	P	P	P	NM_001013841
A_21_P0012331		4424	11175	2797	7283	1774	7627	2929	3406	8.36	2.53	7.32	2.60	10.65	4.30	2.40	1.16	1.65	0.27	1.70	P	P	P	P	P	P	P	P	ENST00000546734
A_23_P87560	BTG1	3886	9903	3652	8522	6035	9194	5010	6221	8.44	2.55	6.48	2.33	4.03	1.52	3.45	1.24	0.65	0.82	0.60	P	P	P	P	P	P	P	P	NM_001731
A_23_P164179	TOB1	3853	2866	3298	2154	2738	1755	2501	2249	-2.13	0.74	-3.27	0.65	-3.25	0.64	-1.70	0.90	0.98	1.40	0.86	P	P	P	P	P	P	P	P	NM_005749
A_33_P3239347	NKX3-1	3733	6664	1639	3527	2511	4842	2007	2107	4.17	1.79	5.86	2.15	4.79	1.93	0.77	1.05	0.90	0.54	1.08	P	P	P	P	P	P	P	P	NM_006167
A_33_P3237634	TSC22D3	3595	7923	3169	7926	2443	6807	1953	2526	5.69	2.20	7.01	2.50	7.48	2.79	4.11	1.29	1.11	0.46	1.26	P	P	P	P	P	P	P	P	NM_004089
A_21_P0012330		3582	9087	2722	7522	1818	7421	2916	3540	6.70	2.54	7.78	2.76	10.27	4.08	3.09	1.21	1.48	0.30	1.61	P	P	P	P	P	P	P	P	ENST00000546734
A_33_P3214209	GUSBP1	3507	5461	4090	6327	3851	5965	4051	4816	3.19	1.56	3.33	1.55	3.19	1.55	2.76	1.19	1.00	0.77	0.99	P	P	P	P	P	P	P	P	NR_027027
A_23_P57370	CECR5	2828	4750	2319	3953	2120	3933	2490	2741	3.73	1.68	4.08	1.70	4.51	1.86	1.53	1.10	1.09	0.59	1.10	P	P	P	P	P	P	P	P	NM_033070
A_23_P57856	BCL6	2577	6016	3369	7683	2704	5870	2519	3373	6.10	2.33	6.31	2.28	5.66	2.17	4.65	1.34	0.95	0.62	0.93	P	P	P	P	P	P	P	P	NM_001130845
A_23_P25150	HOXC9	2489	6931	2633	6541	2942	6730	2514	3516	7.37	2.78	6.96	2.48	6.04	2.29	5.35	1.40	0.92	0.61	0.82	P	P	P	P	P	P	P	P	NM_006897
A_23_P138665	GLUD1	2311	4669	2458	5116	2405	4143	2427	2811	5.06	2.02	5.61	2.08	3.97	1.72	2.34	1.16	0.83	0.67	0.85	P	P	P	P	P	P	P	P	NM_005271
A_23_P69100	ARHGEF26	2236	3751	1954	3260	2946	4686	3376	3450	3.73	1.68	3.92	1.67	3.39	1.59	0.34	1.02	0.95	0.64	0.95	P	P	P	P	P	P	P	P	NM_015595
A_33_P3369844	CD24	2209	3428	2501	3865	5080	7853	3061	3251	3.16	1.55	3.33	1.55	4.17	1.55	0.96	1.06	1.00	0.69	1.00	P	P	P	P	P	P	P	P	NM_013230
A_21_P0012332		2176	5874	1577	4326	917	3948	1762	1874	7.15	2.70	7.72	2.74	10.66	4.30	0.98	1.06	1.57	0.25	1.59	P	P	P	P	P	P	P	P	ENST00000453395
A_23_P25974	TTC7B	2009	2871	2743	4022	2626	4278	2846	3086	2.57	1.43	2.93	1.47	3.56	1.63	1.29	1.08	1.11	0.67	1.14	P	P	P	P	P	P	P	P	NM_001010854
A_23_P138435	ZMIZ1	1979	3268	2530	3893	2797	4315	2238	2925	3.61	1.65	3.29	1.54	3.16	1.54	4.27	1.31	1.00	0.85	0.93	P	P	P	P	P	P	P	P	NM_020338
A_24_P381962	AP1G1	1950	3373	2317	3890	2365	4146	2310	2735	3.94	1.73	3.96	1.68	4.10	1.75	2.69	1.18	1.04	0.68	1.01	P	P	P	P	P	P	P	P	NM_001030007
A_23_P30666	TNFRSF21	1940	1410	3444	1800	6245	3794	3166	2875	-2.29	0.73	-4.97	0.52	-3.65	0.61	-1.54	0.91	1.16	1.49	0.84	P	P	P	P	P	P	P	P	NM_014452
A_24_P125871	RIPK4	1890	3197	2334	3701	2458	3691	2206	2476	3.78	1.69	3.53	1.59	2.97	1.50	1.84	1.12	0.95	0.75	0.89	P	P	P	P	P	P	P	P	NM_020639
A_23_P135239	TLE1	1866	1042	4519	2176	5807	3478	3665	3379	-3.93	0.56	-5.60	0.48	-3.75	0.60	-1.30	0.92	1.24	1.54	1.07	P	P	P	P	P	P	P	P	NM_005077
A_23_P103588	HMGCS2	1695	677	1957	939	1617	740	1153	920	-6.19	0.40	-5.38	0.48	-5.50	0.46	-2.91	0.80	0.95	1.74	1.15	P	P	P	P	P	P	P	P	NM_005518
A_23_P136671	UGT2B7	1601	560	344	176	331	161	458	323	-7.07	0.35	-3.88	0.51	-3.61	0.49	-3.21	0.71	0.95	1.45	1.39	P	P	P	P	P	P	P	P	NM_001074
A_23_P152055	EFTUD1	1498	3669	1936	4639	2011	4493	1939	2260	6.45	2.45	6.69	2.40	5.87	2.23	2.44	1.17	0.93	0.52	0.91	P	P	P	P	P	P	P	P	NM_024580
A_24_P20630	LEF1	1460	767	3361	1610	3217	1697	2461	2111	-4.34	0.53	-5.64	0.48	-4.68	0.53	-2.45	0.86	1.10	1.63	1.00	P	P	P	P	P	P	P	P	NM_016269
A_21_P0005618		1351	2562	5443	9053	6931	10666	3909	4792	4.61	1.90	5.12	1.66	4.13	1.54	3.24	1.23	0.93	0.80	0.81	P	P	P	P	P	P	P	P	ENST00000561761
A_23_P129413	DPEP3	1323	1189	421	402	251	387	283	300	-0.73	0.90	-0.26	0.96	2.19	1.54	0.52	1.06	1.61	0.69	1.71	P	P	P	P	P	P	P	P	NM_022357
A_24_P50753	NUDT4	1299	926	1683	1005	1388	854	1221	1090	-2.29	0.71	-3.78	0.60	-3.41	0.62	-1.46	0.89	1.03	1.45	0.86	P	P	P	P	P	P	P	P	NM_199040
A_24_P80135	PTPN18	1215	1735	1338	2043	1167	1954	1393	1569	2.40	1.43	3.24	1.53	3.76	1.67	1.54	1.13	1.10	0.67	1.17	P	P	P	P	P	P	P	P	NM_014369
A_23_P395172	ABHD2	1165	1999	1267	2007	1176	1923	1145	1349	3.63	1.72	3.52	1.58	3.59	1.64	2.12	1.18	1.03	0.72	0.95	P	P	P	P	P	P	P	P	NM_007011
A_23_P143143	ID2	1139	955	587	452	678	417	546	472	-1.20	0.84	-1.92	0.77	-3.42	0.61	-1.86	0.87	0.80	1.41	0.73	P	P	P	P	P	P	P	P	NM_002166
A_23_P45361	GLUD2	1125	2124	1198	2110	942	1590	987	1144	4.58	1.89	4.33	1.76	3.70	1.69	1.91	1.16	0.96	0.69	0.89	P	P	P	P	P	P	P	P	NM_012084
A_23_P7172	PGM2	1091	2050	1968	3638	1863	3271	1802	2193	4.24	1.88	4.70	1.85	4.11	1.76	3.13	1.22	0.95	0.69	0.93	P	P	P	P	P	P	P	P	NM_018290
A_33_P3269110	GUCY1A3	1047	763	1632	945	1385	817	1522	1386	-2.14	0.73	-4.00	0.58	-3.71	0.59	-1.20	0.91	1.02	1.54	0.81	P	P	P	P	P	P	P	P	NM_001130687
A_24_P55496	OSR2	1033	1711	561	790	415	624	365	428	3.39	1.66	2.51	1.41	2.89	1.50	1.49	1.17	1.07	0.78	0.91	P	P	P	P	P	P	P	P	NM_053001
A_23_P210015	PTPN18	955	1165	953	1327	817	1341	815	918	1.33	1.22	2.43	1.39	3.51	1.64	1.55	1.13	1.18	0.69	1.34	P	P	P	P	P	P	P	P	NM_014369
A_23_P80382	PRR5	941	1129	754	944	583	894	668	649	1.22	1.20	1.65	1.25	3.03	1.53	-0.37	0.97	1.22	0.63	1.28	P	P	P	P	P	P	P	P	NM_015366
A_23_P381102	CCDC74B	934	1695	155	290	102	194	115	127	4.01	1.81	3.66	1.88	3.25	1.90	0.97	1.11	1.01	0.58	1.05	P	P	P	P	P	P	P	P	NM_207310
A_23_P394567	KIAA1467	934	1308	726	1123	430	679	539	577	2.26	1.40	3.20	1.55	3.25	1.58	0.88	1.07	1.02	0.68	1.13	P	P	P	P	P	P	P	P	NM_020853
A_23_P250164	HGD	916	811	198	139	253	160	289	279	-0.83	0.88	-2.05	0.70	-2.30	0.63	-0.32	0.97	0.90	1.53	0.71	P	P	P	P	P	P	P	P	NM_000187
A_33_P3368785	DLGAP1-AS1	913	1562	992	2083	1046	2120	721	1002	3.61	1.71	5.44	2.10	5.16	2.03	4.24	1.39	0.97	0.68	1.19	P	P	P	P	P	P	P	P	NR_024101
A_21_P0002781	LINC01213	901	1310	1454	2595	1087	1726	1084	1233	2.51	1.45	4.43	1.78	3.37	1.59	1.67	1.14	0.89	0.72	1.09	P	P	P	P	P	P	P	P	NR_110167
A_24_P38951	RELT	894	1087	583	797	464	732	586	627	1.31	1.22	2.30	1.37	3.22	1.58	0.88	1.07	1.15	0.68	1.30	P	P	P	P	P	P	P	P	NM_032871
A_23_P210581	KCNG1	875	2016	491	1035	876	1671	884	1149	5.62	2.30	5.47	2.11	4.57	1.91	3.39	1.30	0.90	0.68	0.83	P	P	P	P	P	P	P	P	NM_002237
A_33_P3409159	SLC22A23	863	1634	718	1191	707	1217	617	717	4.30	1.89	3.72	1.66	3.84	1.72	1.95	1.16	1.04	0.68	0.91	P	P	P	P	P	P	P	P	NM_015482
A_21_P0014711	EIF4E2	851	1198	700	836	805	1238	689	729	2.30	1.41	1.31	1.19	3.05	1.54	0.74	1.06	1.29	0.69	1.09	P	P	P	P	P	P	P	P	NM_001282958
A_33_P3314386	TOR1AIP1	806	1496	937	1434	957	1699	719	849	4.16	1.86	3.12	1.53	4.06	1.77	2.14	1.18	1.16	0.66	0.96	P	P	P	P	P	P	P	P	NM_001267578
A_23_P103011	RAB36	802	449	789	489	568	335	616	546	-3.92	0.56	-3.51	0.62	-3.70	0.59	-1.55	0.89	0.95	1.50	1.05	P	P	P	P	P	P	P	P	NM_004914
A_23_P46470	ERRFI1	790	868	921	1097	947	1452	1013	1018	0.63	1.10	1.29	1.19	3.03	1.53	0.07	1.00	1.29	0.65	1.40	P	P	P	P	P	P	P	P	NM_018948
A_23_P31407	AGR2	764	294	268	134	278	156	350	296	-6.44	0.38	-3.99	0.50	-2.92	0.56	-1.53	0.85	1.11	1.52	1.45	P	P	P	P	P	P	P	P	NM_006408
A_33_P3385870	CCNG2	764	525	842	581	900	578	931	805	-2.53	0.69	-2.72	0.69	-3.11	0.64	-1.86	0.87	0.93	1.35	0.93	P	P	P	P	P	P	P	P	NM_004354
A_33_P3292028	DPP9	759	1563	769	1352	681	1214	759	862	4.85	2.06	4.14	1.76	4.09	1.78	1.65	1.14	1.01	0.64	0.87	P	P	P	P	P	P	P	P	NM_139159
A_23_P65307	SLITRK6	758	333	1314	660	1265	752	1200	1055	-5.55	0.44	-5.04	0.50	-3.66	0.59	-1.65	0.88	1.18	1.48	1.35	P	P	P	P	P	P	P	P	NM_032229
A_23_P93269	ZNF165	750	2237	828	2020	914	2085	917	1026	7.36	2.98	6.55	2.44	6.02	2.28	1.45	1.12	0.93	0.49	0.76	P	P	P	P	P	P	P	P	NM_003447
A_23_P140450	SLC27A2	747	1116	922	1408	934	1468	1114	1160	2.70	1.49	3.11	1.53	3.20	1.57	0.53	1.04	1.03	0.66	1.05	P	P	P	P	P	P	P	P	NM_003645
A_23_P394064	PTRF	738	921	246	369	316	488	189	182	1.49	1.25	2.36	1.50	3.08	1.54	-0.36	0.96	1.03	0.62	1.24	P	P	P	P	P	P	P	P	NM_012232
A_23_P33791	SSBP2	734	450	998	758	1076	702	930	951	-3.29	0.61	-2.01	0.76	-3.00	0.65	0.29	1.02	0.86	1.57	1.06	P	P	P	P	P	P	P	P	NM_012446
A_33_P3369969	ATXN1L	700	1008	868	1133	621	969	758	840	2.45	1.44	1.96	1.31	3.15	1.56	1.32	1.11	1.20	0.71	1.08	P	P	P	P	P	P	P	P	NM_001137675
A_23_P139418	GALNT18	689	2079	1218	3488	1373	3558	1139	1520	7.43	3.02	8.05	2.86	6.95	2.59	3.72	1.33	0.91	0.51	0.86	P	P	P	P	P	P	P	P	NM_198516
A_21_P0012258	MAD1L1	678	2240	528	1145	464	1082	426	555	8.04	3.30	5.68	2.17	5.98	2.33	3.42	1.30	1.08	0.56	0.71	P	P	P	P	P	P	P	P	NM_003550
A_33_P3569068	MAD1L1	672	2182	549	1140	443	1108	431	591	7.92	3.25	5.36	2.08	6.48	2.50	4.09	1.37	1.20	0.55	0.77	P	P	P	P	P	P	P	P	NM_003550
A_21_P0014862		653	918	473	795	423	685	492	520	2.29	1.41	3.81	1.68	3.41	1.62	0.72	1.06	0.96	0.65	1.15	P	P	P	P	P	P	P	P	
A_33_P3403044	MAT2A	652	1073	495	881	510	776	520	590	3.35	1.65	4.24	1.78	2.97	1.52	1.64	1.14	0.85	0.75	0.92	P	P	P	P	P	P	P	P	NM_005911
A_21_P0014553		652	997	530	761	633	972	760	772	2.85	1.53	2.66	1.44	3.04	1.54	0.20	1.02	1.07	0.66	1.00	P	P	P	P	P	P	P	P	ENST00000548057
A_23_P92860	CCNO	641	348	276	148	213	113	231	170	-4.12	0.54	-3.60	0.54	-3.17	0.53	-2.86	0.73	0.99	1.38	0.98	P	P	P	P	P	P	P	P	NM_021147
A_23_P502957	CDH26	626	968	289	523	250	482	204	237	2.93	1.55	3.43	1.81	3.31	1.93	1.39	1.16	1.07	0.60	1.25	P	P	P	P	P	P	P	P	NM_021810
A_32_P115505	LTN1	620	388	365	236	303	157	280	278	-3.16	0.63	-2.53	0.65	-3.28	0.52	-0.07	0.99	0.81	1.91	0.83	P	P	P	P	P	P	P	P	NM_015565
A_21_P0011196		596	1447	408	1095	248	954	405	493	5.97	2.43	7.24	2.68	9.52	3.85	2.54	1.22	1.43	0.32	1.59	P	P	P	P	P	P	P	P	ENST00000550892
A_33_P3376971	CHAC1	587	558	586	586	556	923	678	727	-0.35	0.95	0.01	1.00	3.59	1.66	0.90	1.07	1.66	0.65	1.75	P	P	P	P	P	P	P	P	NM_024111
A_23_P254888	ZYX	580	1034	673	1072	575	1186	467	552	3.89	1.78	3.42	1.59	5.12	2.06	2.18	1.18	1.29	0.57	1.16	P	P	P	P	P	P	P	P	NM_003461
A_21_P0000925	LINC01341	572	1399	419	1080	384	1024	266	343	6.02	2.45	6.95	2.58	6.92	2.66	2.36	1.29	1.03	0.48	1.09	P	P	P	P	P	P	P	P	AK022825
A_23_P128215	SOCS2	570	283	806	488	1049	539	938	875	-3.17	0.50	-3.67	0.61	-4.68	0.51	-0.89	0.93	0.85	1.81	1.04	P	P	P	P	P	P	P	P	NM_003877
A_24_P313186	CALM1	568	851	717	1308	645	1070	693	738	2.72	1.50	4.41	1.82	3.58	1.66	0.83	1.07	0.91	0.64	1.11	P	P	P	P	P	P	P	P	NM_006888
A_23_P34093	G6PD	567	897	847	2130	740	1958	1006	1338	3.09	1.58	6.77	2.52	6.88	2.65	3.68	1.33	1.05	0.50	1.67	P	P	P	P	P	P	P	P	NM_000402
A_21_P0000924	LINC01341	565	1390	464	1199	409	1018	268	363	6.06	2.46	6.96	2.58	6.44	2.49	2.82	1.36	0.96	0.55	1.01	P	P	P	P	P	P	P	P	AK022825
A_33_P3301445		550	953	508	990	447	762	470	476	3.69	1.73	4.90	1.95	3.78	1.71	0.17	1.01	0.87	0.59	0.98	P	P	P	P	P	P	P	P	
A_23_P500381	HTR7	547	783	510	717	497	746	502	543	2.40	1.43	2.50	1.41	2.89	1.50	1.03	1.08	1.07	0.72	1.05	P	P	P	P	P	P	P	P	NM_019859
A_21_P0000662	RAB6C-AS1	543	808	700	1175	655	1160	681	704	2.67	1.49	3.80	1.68	4.05	1.77	0.43	1.03	1.06	0.58	1.19	P	P	P	P	P	P	P	P	NR_036537
A_23_P152406	CAPNS2	540	858	490	702	297	471	346	403	3.11	1.59	2.63	1.43	3.26	1.58	1.43	1.17	1.11	0.74	1.00	P	P	P	P	P	P	P	P	NM_032330
A_23_P316381	ACOX3	538	754	345	649	251	385	265	282	2.27	1.40	4.64	1.88	2.17	1.53	0.58	1.06	0.81	0.69	1.09	P	P	P	P	P	P	P	P	NM_003501
A_23_P64808	HOXC13	534	1405	450	1150	570	1181	421	538	6.51	2.63	6.89	2.56	5.16	2.07	3.18	1.28	0.81	0.62	0.79	P	P	P	P	P	P	P	P	NM_017410
A_33_P3216890	PAG1	532	942	502	841	357	711	413	501	3.85	1.77	3.78	1.67	4.86	1.99	2.50	1.21	1.19	0.61	1.12	P	P	P	P	P	P	P	P	NM_018440
A_23_P210763	JAG1	525	387	368	230	484	250	306	294	-2.05	0.74	-2.72	0.63	-3.32	0.52	-0.38	0.96	0.83	1.86	0.70	P	P	P	P	P	P	P	P	NM_000214
A_23_P105002	ROM1	517	963	887	2156	679	2924	769	1056	4.19	1.86	6.52	2.43	10.66	4.30	4.09	1.37	1.77	0.32	2.31	P	P	P	P	P	P	P	P	NM_000327
A_23_P21495	FCGBP	516	545	631	504	1775	1094	657	859	0.36	1.06	-1.65	0.80	-3.54	0.62	3.46	1.31	0.77	2.12	0.58	P	P	P	P	P	P	P	P	NM_003890
A_33_P3357651	KRTAP10-12	513	817	532	1054	528	806	367	475	3.13	1.59	5.02	1.98	2.99	1.53	2.39	1.29	0.77	0.85	0.96	P	P	P	P	P	P	P	P	NM_198699
A_32_P174572	HTR7P1	505	771	547	738	507	825	566	595	2.84	1.53	2.20	1.35	3.44	1.63	0.65	1.05	1.20	0.65	1.06	P	P	P	P	P	P	P	P	NR_002774
A_23_P92727	RAI14	499	726	409	642	520	797	478	622	2.53	1.46	3.32	1.57	3.02	1.53	3.40	1.30	0.97	0.85	1.05	P	P	P	P	P	P	P	P	NM_015577
A_33_P3305790	NOS3	498	397	1519	930	1736	1051	534	561	-1.54	0.80	-3.59	0.61	-3.53	0.61	0.65	1.05	0.99	1.74	0.76	P	P	P	P	P	P	P	P	NM_000603
A_21_P0002852	SEMA3B-AS1	480	499	486	718	346	538	234	252	0.25	1.04	2.86	1.48	3.13	1.56	0.69	1.08	1.05	0.69	1.50	P	P	P	P	P	P	P	P	NR_110702
[illegible]	[illegible]	[illegible]	[illegible]	[illegible]	[illegible]	[illegible]	[illegible]	[illegible]	[illegible]	[illegible]	[illegible]	[illegible]	[illegible]	[illegible]	[illegible]	[illegible]	[illegible]	[illegible]	[illegible]	[illegible]	[illegible]	[illegible]	[illegible]	[illegible]	[illegible]	[illegible]	[illegible]	[illegible]	[illegible]
A_21_P0010662	LOC100506023	204	371	257	532	204	339	200	272	2.69	1.82	4.21	2.07	2.56	1.66	2.83	1.36	0.80	0.82	0.91	P	P	P	P	P	P	P	P	NR_037845
A_24_P140608	HBEGF	202	427	248	624	154	370	137	177	3.38	2.11	5.36	2.52	4.43	2.40	2.41	1.30	0.95	0.54	1.14	P	P	P	P	P	P	P	P	NM_001945
A_23_P170901	PACRG	199	122	296	218	202	131	361	374	-2.23	0.61	-1.75	0.74	-2.15	0.65	0.34	1.04	0.88	1.59	1.06	P	P	P	P	P	P	P	P	NM_152410
A_32_P172141	CDON	196	332	197	294	191	352	184	233	2.39	1.70	2.33	1.49	3.09	1.84	2.16	1.26	1.23	0.69	1.09	P	P	P	P	P	P	P	P	NM_016952
A_23_P152428	MARVELD3	194	301	173	259	173	286	164	194	1.97	1.55	2.34	1.50	2.56	1.66	1.55	1.18	1.11	0.71	1.07	P	P	P	P	P	P	P	P	NM_052858
A_24_P305570	RIN2	189	370	124	206	195	317	137	155	3.04	1.96	2.93	1.66	2.47	1.63	1.13	1.13	0.98	0.69	0.83	P	P	P	P	P	P	P	P	NM_018993
A_23_P53193	SYTL2	181	110	363	239	403	223	245	231	-2.24	0.61	-2.42	0.66	-2.97	0.55	-0.53	0.94	0.84	1.71	0.91	P	P	P	P	P	P	P	P	NM_032943
A_33_P3393088	RAB6C-AS1	176	357	267	464	216	353	193	206	3.21	2.03	3.21	1.74	2.48	1.63	0.62	1.07	0.94	0.65	0.80	P	P	P	P	P	P	P	P	BC044241
A_23_P339053		172	598	157	695	205	800	241	274	5.63	3.48	8.63	4.43	9.61	3.90	1.17	1.13	0.88	0.29	1.12	P	P	P	P	P	P	P	P	ENST00000569656
A_21_P0014313		170	109	162	145	199	121	210	211	-2.00	0.64	-0.62	0.90	-2.50	0.61	0.05	1.00	0.68	1.66	0.94	P	P	P	P	P	P	P	P	ENST00000573315
A_23_P125596	RPS6KA6	162	289	183	254	108	170	151	165	2.60	1.78	1.91	1.39	2.30	1.57	0.80	1.09	1.13	0.69	0.89	P	P	P	P	P	P	P	P	NM_014496
A_24_P879740	MAP1B	158	142	535	383	414	264	452	491	-0.50	0.90	-2.44	0.72	-2.25	0.64	1.09	1.09	0.89	1.70	0.71	P	P	P	P	P	P	P	P	NM_005909
A_23_P35684	INPP5F	154	158	202	175	164	247	178	167	0.12	1.03	-0.81	0.87	2.07	1.50	-0.56	0.94	1.73	0.63	1.46	P	P	P	P	P	P	P	P	NM_014937
A_24_P191664	GOLIM4	129	371	124	277	191	383	133	199	4.77	2.88	4.65	2.23	3.52	2.00	3.67	1.49	0.90	0.74	0.70	P	P	P	P	P	P	P	P	NM_014498
A_33_P3540143	IL17RA	120	161	138	184	128	202	182	196	1.35	1.35	1.69	1.34	2.30	1.57	0.69	1.08	1.18	0.68	1.17	P	P	P	P	P	P	P	P	NM_014339
A_21_P0014276		118	195	151	265	148	327	167	206	2.25	1.65	3.27	1.76	4.01	2.21	1.96	1.24	1.26	0.56	1.34	P	P	P	P	P	P	P	P	
A_23_P147805	UPP1	118	229	153	284	121	250	105	111	3.00	1.94	3.60	1.86	3.68	2.07	0.21	1.06	1.11	0.51	1.07	P	P	P	P	P	P	P	P	NM_001287428
A_23_P118042	LRRC36	115	232	217	454	246	403	176	207	3.19	2.02	4.28	2.09	2.49	1.64	1.52	1.18	0.78	0.72	0.81	P	P	P	P	P	P	P	P	NM_018296
A_33_P3307955	ABCD3	114	230	231	639	162	353	207	254	3.19	2.02	5.91	2.77	3.94	2.18	1.90	1.23	0.79	0.56	1.08	P	P	P	P	P	P	P	P	NM_001122674
A_33_P3719214		108	113	162	153	124	189	132	119	0.18	1.04	-0.33	0.94	2.16	1.53	-0.89	0.91	1.62	0.59	1.47	P	P	P	P	P	P	P	P	ENST00000456627
A_24_P497186	IRF2BP2	108	103	175	150	238	153	185	168	-0.20	0.96	-0.89	0.86	-2.21	0.64	-0.87	0.91	0.75	1.41	0.67	P	P	P	P	P	P	P	P	NM_182972

Supplemental Table S4B 25D3 target genes not mediate by VDR (8).

ProbeID	GeneSymbol	siCT_V1_signal	siCT_1,25D3_signal	siCT_V2_signal	siCT_25D3_signal	siCYP27B1_V_signal	siCYP27B1_25D3_signal	siCYP27B1_siVDR_V_signal	siCYP27B1_siVDR_25D3_signal	siCT_1,25D3/siCT_V1_Zscore	siCT_1,25D3/siCT_V1_ratio	siCT_25D3/siCT_V2_Zscore	siCT_25D3/siCT_V2_ratio	siCYP27B1_25D3/siCYP27B1_V_Zscore	siCYP27B1_25D3/siCYP27B1_ratio	siCYP27B1_siVDR_25D3/siCYP27B1_siVDR_V_Zscore	siCYP27B1_siVDR_25D3/siCYP27B1_siVDR_V_ratio	siCYP27B1_25/V/siCT_25D3/V2_ratio	siCYP27B1_siVDR_25D3/V/siCYP27B1_25D3/V_ratio	siC27B1_25D3/V/siCT_1,25D3/V1_ratio	siCT_V1_flag	siCT_1,25D3_flag	siCT_V2_flag	siCT_25D3_flag	siCYP27B1_V_flag	siCYP27B1_25D3_flag	siCYP27B1_siVDR_V_flag	siCYP27B1_siVDR_25D3_flag	PrimaryAccession
A_23_P16523	GDF15	40397	108529	141974	269139	102700	185340	60947	93882	12.33	2.69	9.24	1.90	8.54	1.80	5.54	1.54	0.95	0.85	0.67	P	P	P	P	P	P	P	P	NM_004864
A_24_P252497	TRIB1	5552	11989	6847	14438	5608	11955	4526	6993	6.94	2.16	7.50	2.11	7.24	2.13	6.94	1.54	1.01	0.72	0.99	P	P	P	P	P	P	P	P	NM_025195
A_23_P15174	MT1F	3336	812	1243	532	2966	1733	1724	1077	-10.17	0.24	-6.22	0.43	-3.93	0.58	-6.06	0.62	1.37	1.07	2.40	P	P	P	P	P	P	P	P	NM_005949
A_23_P29067	TMPRSS2	900	21619	469	11703	478	8022	430	1637	22.88	24.01	24.63	24.96	20.59	16.77	17.24	3.81	0.67	0.23	0.70	P	P	P	P	P	P	P	P	NM_005656
A_23_P500353	KCNN2	401	4810	388	6010	660	8110	689	3314	16.73	12.00	20.09	15.50	18.32	12.29	20.25	4.81	0.79	0.39	1.02	P	P	P	P	P	P	P	P	NM_021614
A_24_P366777	NOTCH2NL	311	603	249	364	290	489	227	341	4.44	1.94	2.20	1.46	3.70	1.69	3.76	1.50	1.16	0.89	0.87	P	P	P	P	P	P	P	P	ENST00000344859
A_23_P71170	TRPV6	270	1294	398	2149	499	2375	342	570	10.54	4.79	12.37	5.40	11.02	4.76	6.60	1.67	0.88	0.35	0.99	P	P	P	P	P	P	P	P	NM_018646
A_21_P0012089	FAR2P1	213	518	278	585	183	382	138	223	4.01	2.43	4.31	2.10	3.73	2.09	4.44	1.62	0.99	0.77	0.86	P	P	P	P	P	P	P	P	NR_026758

Supplemental Table S5 1,25D3 target genes in siCT cells (264).

ProbeID	GeneSymbol	siCT_V1_signal	siCT_1,25D3_signal	siCT_V2_signal	siCT_25D3_signal	siCYP27B1_V_signal	siCYP27B1_25D3_signal	siCYP27B1_siVDR_V_signal
A_23_P16523	GDF15	40397	108529	141974	269139	102700	185340	60947
A_33_P3285545	CLDN4	31397	48717	50313	80442	38373	64904	38181
A_23_P106844	MT2A	27944	7668	29692	14177	30083	25807	33511
A_23_P62932	ATP1B1	19425	12073	16975	12417	17797	12283	14177
A_23_P139786	OASL	13441	21698	2060	3090	1354	1560	359
A_24_P208345	SLC45A3	11280	16965	11201	20087	9303	17392	7790
A_21_P0012591	UGT2B11	11142	5801	5880	4425	5956	3639	6228
A_33_P3233645	MT1G	10806	2088	6493	2983	9896	7785	8926
A_23_P7342	UGT2B10	10174	5702	5738	4724	5874	3842	6199
A_33_P3220837	MAFB	9972	16102	14410	22495	22148	32617	16495
A_33_P3349414	ZBTB43	9850	15747	7058	9545	7621	8851	5776
A_23_P427703	MT1L	8823	1995	5265	2586	6471	5358	6418
A_23_P11800	CAMK2N1	8656	14358	12737	16180	24250	26364	27391
A_23_P37983	MT1B	8565	2176	6045	2895	7709	6365	7465
A_23_P31896	ST3GAL1	8397	16301	11883	17673	13013	17993	11851
A_23_P113111	AR	7199	19386	7131	19811	7680	22300	8545
A_33_P3315314	MT1HL1	7062	2098	5552	2867	7058	5971	6508
A_23_P206724	MT1E	7058	1617	4099	1958	5511	4716	4990
A_33_P3350056	MT1X	5959	2087	3706	2150	4575	4111	4709
A_24_P252497	TRIB1	5552	11989	6847	14438	5608	11955	4526
A_23_P382188	STAP2	4876	7721	4088	7511	5905	9139	4463
A_23_P401568	MAT2A	4795	7941	4686	8200	5471	8029	5492
A_23_P125383	TMEFF2	4716	7137	6375	8617	5355	5760	6621
A_23_P212968	UGT2B11	4593	2949	2832	2562	2569	1863	2843
A_21_P0012331		4424	11175	2797	7283	1774	7627	2929
A_33_P3288364	SPATA2L	4122	6216	3066	4634	2556	3735	2749
A_23_P87560	BTG1	3886	9903	3652	8522	6035	9194	5010
A_23_P74359	CSRP1	3806	2060	2849	2080	2651	2323	3277
A_33_P3239347	NKX3-1	3733	6664	1639	3527	2511	4842	2007
A_23_P31143	TPD52L1	3689	5680	6533	9348	6636	8926	5657
A_33_P3237634	TSC22D3	3595	7923	3169	7926	2443	6807	1953
A_21_P0012330		3582	9087	2722	7522	1818	7421	2916
A_33_P3214209	GUSBP1	3507	5461	4090	6327	3851	5965	4051
A_23_P15174	MT1F	3336	812	1243	532	2966	1733	1724
A_23_P57370	CECR5	2828	4750	2319	3953	2120	3933	2490
A_23_P60499	ZNF462	2681	4321	2644	3306	3205	4136	2378
A_23_P106002	NFKBIA	2662	4056	3563	5183	6684	8163	4030
A_23_P57856	BCL6	2577	6016	3369	7683	2704	5870	2519
A_23_P24004	IFIT2	2536	4071	1165	1464	952	969	399
A_23_P25150	HOXC9	2489	6931	2633	6541	2942	6730	2514
A_33_P3679768		2433	3844	1113	1618	841	1258	1034
A_23_P255663	MANEA	2352	4124	2158	3440	3824	5352	2248
A_33_P3246804	STARD3	2351	3739	3006	3805	3521	4329	3504
A_23_P138665	GLUD1	2311	4669	2458	5116	2405	4143	2427
A_23_P69100	ARHGEF26	2236	3751	1954	3260	2946	4686	3376
A_33_P3369844	CD24	2209	3428	2501	3865	5080	7853	3061
A_21_P0012332		2176	5874	1577	4326	917	3948	1762
A_24_P268786	MYNN	2161	3244	1773	2324	1205	1241	1834
A_23_P110212	ACSL1	2142	4416	1343	2444	1589	2355	1861
A_23_P384517	GYG1	2063	3196	2331	2909	2033	2428	2294
A_23_P132669	GLT8D1	2001	3015	1856	2599	1802	2209	1924
A_23_P138435	ZMIZ1	1979	3268	2530	3893	2797	4315	2238
A_24_P381962	AP1G1	1950	3373	2317	3890	2365	4146	2310
A_24_P125871	RIPK4	1890	3197	2334	3701	2458	3691	2206
A_23_P135239	TLE1	1866	1042	4519	2176	5807	3478	3665
A_24_P943393	AHNAK	1812	1166	2549	1603	3016	2339	2372
A_24_P108311	NEDD4L	1778	2716	3495	4627	2705	3418	2531
A_23_P103588	HMGCS2	1695	677	1957	939	1617	740	1153
A_32_P47107		1603	2744	4604	7632	6179	9008	3421
A_23_P136671	UGT2B7	1601	560	344	176	331	161	458
A_24_P71244	PIK3CD	1569	2466	1272	1764	1188	1596	1505
A_33_P3409210	LOC100128340	1544	965	1084	858	823	618	826
A_23_P14062	NUP107	1539	2428	1835	2673	2189	2853	2250
A_23_P152055	EFTUD1	1498	3669	1936	4639	2011	4493	1939
A_21_P0013960	LOC101930246	1494	2242	4882	6803	3002	4218	3922
A_21_P0012871	LOC100128340	1483	938	1151	945	951	721	890
A_24_P20630	LEF1	1460	767	3361	1610	3217	1697	2461
A_24_P149713	ZBTB43	1452	2634	943	1275	892	1088	717
A_23_P93141	GSTA5	1440	923	1155	937	1508	1375	1136
A_33_P3236868	MT1X	1423	750	1046	779	1241	1373	1379
A_21_P0012868	LOC100128340	1391	854	965	737	778	593	770
A_23_P1782	CD82	1361	2268	1046	1457	815	1191	691
A_21_P0005618		1351	2562	5443	9053	6931	10666	3909
A_23_P147431	LYN	1345	2082	1667	2477	1532	1791	1501
A_19_P00809119	CASC15	1191	782	595	370	1352	1189	589
A_23_P395172	ABHD2	1165	1999	1267	2007	1176	1923	1145
A_23_P133470	PJA2	1128	2049	866	1548	581	810	746
A_23_P45361	GLUD2	1125	2124	1198	2110	942	1590	987
A_23_P7172	PGM2	1091	2050	1968	3638	1863	3271	1802
A_24_P55496	OSR2	1033	1711	561	790	415	624	365
A_33_P3316539	SLC7A2	1007	656	1430	1007	2480	1999	2239
A_24_P46130	ACPP	985	1541	1466	1822	1784	1975	1799
A_23_P381102	CCDC74B	934	1695	155	290	102	194	115
A_33_P3368785	DLGAP1-AS1	913	1562	992	2083	1046	2120	721
A_23_P29067	TMPRSS2	900	21619	469	11703	478	8022	430
A_24_P46953	SGK3	893	323	484	470	470	469	578
A_23_P210581	KCNG1	875	2016	491	1035	876	1671	884
A_33_P3409159	SLC22A23	863	1634	718	1191	707	1217	617
A_32_P126311	SLC38A9	855	1482	1194	1797	1427	1924	1664
A_23_P435941	SAMD1	836	468	394	338	326	309	392
A_24_P62530	RHOU	833	550	880	679	909	676	617
A_33_P3314386	TOR1AIP1	806	1496	937	1434	957	1699	719
A_33_P103011	RAB36	802	449	789	489	568	335	616
A_33_P3262495	ZNF503	780	1234	738	914	809	803	638
A_23_P215913	CLU	768	465	1338	1001	1034	835	1113
A_23_P31407	AGR2	764	294	268	134	278	156	350
A_33_P3292028	DPP9	759	1563	769	1352	681	1214	759
A_23_P65307	SLITRK6	758	333	1314	660	1265	752	1200
A_23_P93269	ZNF165	750	2237	828	2020	914	2085	917
A_23_P33791	SSBP2	734	450	998	758	1076	702	930
A_23_P139418	GALNT18	689	2079	1218	3488	1373	3558	1139
A_21_P0012258	MAD1L1	678	2240	528	1145	464	1082	426
A_33_P3569068	MAD1L1	672	2182	549	1140	443	1108	431
A_33_P3403044	MAT2A	652	1073	495	881	510	776	520
A_21_P0014553		652	997	530	761	633	972	760
A_23_P320578	RGS16	644	318	611	471	743	594	652
A_23_P92860	CCNO	641	348	276	148	213	113	231
A_24_P658584	SASH1	636	384	333	330	420	397	408
A_23_P502957	CDH26	626	968	289	523	250	482	204
A_32_P115505	LTN1	620	388	365	236	303	157	280
A_24_P570583	ZNF542P	602	910	429	542	455	514	491
A_32_P117170	NAPEPLD	597	1184	981	1645	1557	2072	1196
A_21_P0011196		596	1447	408	1095	248	954	405
A_23_P335981	HOXC12	588	884	195	251	130	171	112
A_23_P254888	ZYX	580	1034	673	1072	575	1186	467
A_23_P377141	VPS13A	574	865	572	713	557	574	589
A_21_P0000925	LINC01341	572	1399	419	1080	384	1024	266
A_23_P128215	SOCS2	570	283	806	488	1049	539	938
A_23_P77731	CRYM	568	340	445	314	452	324	353
A_23_P34093	G6PD	567	897	847	2130	740	1958	1006
A_21_P0000924	LINC01341	565	1390	464	1199	409	1018	268
A_23_P105923	DIO3	553	324	4509	3582	4579	3453	947
A_33_P3301445		550	953	508	990	447	762	470
A_23_P152406	CAPNS2	540	858	490	702	297	471	346
A_33_P3333054	SYBU	539	816	657	743	596	631	746
A_23_P64808	HOXC13	534	1405	450	1150	570	1181	421
A_33_P3216890	PAG1	532	942	502	841	357	711	413
A_23_P145529	PKIB	517	832	649	1085	853	1172	553
A_23_P105002	ROM1	517	963	887	2156	679	2924	769
A_33_P3307660	KLHDC4	514	329	430	393	389	370	407
A_33_P3357651	KRTAP10-12	513	817	532	1054	528	806	367
A_32_P174572	HTR7P1	505	771	547	738	507	825	566
A_33_P3231156		499	149	179	107	215	205	235
A_33_P3390357	SPIRE1	489	964	863	1008	770	851	693
A_23_P23815	SLC30A1	489	228	501	305	614	609	529
A_33_P3267186	ATP2B1	483	748	581	770	960	1120	582
A_24_P396650	RPS6KA1	468	722	623	929	577	770	620
A_33_P3230244	CSMD1	467	843	678	1089	626	885	502
A_23_P409623	PPFIBP2	464	1182	319	691	281	609	276
A_23_P126677	DNASE2B	455	277	525	388	478	343	463
A_33_P3258223	MCM7	453	142	326	238	213	208	346
A_23_P105562	VWF	445	915	555	1012	313	417	457
A_23_P217755	SHROOM2	444	724	243	600	256	628	232
A_23_P103756	OVGP1	440	226	413	192	445	205	403
A_23_P402610	PFAS	438	203	211	214	142	186	260
A_21_P0010925		422	129	167	185	139	154	183
A_23_P143662	PISD	421	649	341	551	392	589	346
A_33_P3296862	C16orf89	421	234	1125	636	1806	1008	939
A_23_P47924	PTPRR	408	754	1075	1292	994	1014	1059
A_23_P8452	LFNG	408	239	383	169	443	282	368
A_32_P61684	PAG1	408	693	691	1177	557	985	540
A_23_P500353	KCNN2	401	4810	388	6010	660	8110	689
A_21_P0000128	MKRN2OS	400	246	413	342	431	372	436
A_23_P320242	KIAA1324L	399	657	456	663	547	693	426
A_23_P143817	MYLK	397	682	442	533	616	658	584
A_32_P215318	ACACA	394	239	371	341	287	293	376
A_23_P418234	PHLPP2	393	590	425	607	407	618	441
A_23_P58266	S100P	378	593	267	296	1321	1225	443
A_23_P65466	RAB2B	377	580	346	371	213	227	331
A_33_P3284951	MCM5	362	195	465	366	316	292	659
A_33_P3314176	FAM46C	351	560	414	658	585	930	446
A_23_P57760	PXYLP1	345	174	383	240	467	363	359
A_33_P3364205	SBDSP1	345	536	427	417	347	318	409
A_32_P19840	LOC100507507	343	976	704	1416	721	2176	435
A_24_P921897	HOOK1	341	1062	282	850	315	966	236
A_23_P57118	TGM3	339	204	284	166	173	118	266
A_19_P00315651	LINC00659	338	202	166	195	149	166	146
A_33_P3241190	XRCC2	335	208	173	150	155	163	214
A_33_P3331641	ZNF503-AS2	333	508	243	355	338	359	276
A_21_P0000053	C16orf52	331	911	433	1310	556	1667	514
A_21_P0003446	FLJ36777	330	172	237	301	271	327	248
A_23_P55518	SMAD7	329	556	269	468	287	461	271
A_33_P3310189	ADRB1	328	194	692	620	575	516	484
A_21_P0011001	PCSK7	327	188	231	221	193	205	256
A_32_P78783	ZNF778	321	114	139	160	148	148	145
A_24_P366777	NOTCH2NL	311	603	249	364	290	489	227
A_21_P0014204	LOC101928915	309	547	674	1053	616	988	1051
A_23_P66481	RTN4RL1	306	193	245	188	287	229	240
A_24_P97836	RAD17	301	193	153	164	137	147	176
A_33_P3409844	HOOK1	301	706	177	430	190	461	175
A_33_P3214463	MESP2	300	484	269	228	167	209	248
A_21_P0002324	SCHLAP1	289	162	269	185	326	221	255
A_33_P3303104	MYNN	289	499	288	340	213	223	269
A_33_P3300975	HOXC4	282	540	391	424	609	669	388
A_33_P3414669	RLIM	280	112	162	150	162	168	190
A_23_P169003	SH2D4A	278	649	259	524	181	350	199
A_23_P29422	GYG1	278	455	541	733	377	549	454
A_24_P144620	TMEM242	275	152	196	203	167	138	157
A_23_P369899	TMEM158	275	170	334	173	552	250	369
A_23_P126836	TNFSF4	274	779	355	860	158	357	192
A_19_P00317897	LOC101928673	271	504	249	492	346	585	316
A_23_P71170	TRPV6	270	1294	398	2149	499	2375	342
A_33_P3246985	PDE4DIP	262	120	312	214	267	172	366
A_33_P3364854	TRPV6	261	1250	286	1814	399	2066	301
A_33_P3398331	MMP24	251	409	304	454	354	556	288
A_21_P0014094		251	138	164	159	169	176	150
A_33_P3409154	SLC22A23	250	390	405	631	364	484	287
A_33_P3250953	SLC35B4	248	529	283	524	248	426	257
A_23_P130359	ARHGAP28	247	140	270	208	257	183	255
A_23_P414273	SMIM3	244	428	349	561	288	524	289
A_24_P187921	MANEA	243	385	275	414	354	548	244
A_33_P3333982	PDE4DIP	241	104	526	323	330	201	371
A_21_P0000651	HERC2P7	240	409	242	387	370	521	332
A_33_P3290562	GLI3	237	468	418	590	754	938	517
A_33_P3360675	PRKAR1B	235	115	156	149	129	122	154
A_23_P217968	SUV420H1	234	144	121	130	145	127	141
A_23_P131899	SDCBP2	233	126	473	165	2093	960	533
A_23_P170733	ANTXR2	232	104	152	123	350	316	246
A_24_P191417	NAB1	232	136	161	164	208	178	192
A_23_P19624	BMP6	230	477	176	905	389	1473	155
A_33_P3215906	NBPF9	227	431	203	302	250	376	196
A_33_P3415843		226	133	179	144	165	153	230
A_21_P0011335	EFTUD1	223	545	197	425	163	405	158
A_19_P00804596	C17orf51	221	141	311	304	377	359	418
A_23_P336198	GLCCI1	220	405	329	388	245	303	308
A_32_P302205	SI	220	137	167	146	267	206	244
A_33_P3365805	CLN5	216	351	452	650	526	723	448
A_21_P0012089	FAR2P1	213	518	278	585	183	382	138
A_33_P3209869	TCP11L1	208	350	219	203	155	146	229
A_23_P58390	C4orf32	208	415	479	760	479	644	447
A_21_P0010662	LOC100506023	204	371	257	532	204	339	200
A_24_P140608	HBEGF	202	427	248	624	154	370	137
A_24_P199500	RNF2	200	394	237	212	269	223	242
A_23_P170901	PACRG	199	122	296	218	202	131	361
A_33_P3368800	NBPF12	197	362	154	240	197	251	133
A_32_P172141	CDON	196	332	197	294	191	352	184
A_33_P3210986	TYW1	195	321	260	257	208	204	255
A_23_P213085	SLC10A7	193	413	161	245	124	159	106
A_24_P305570	RIN2	189	370	124	206	195	317	137
A_23_P119778	SLC39A10	189	540	174	283	163	181	163
A_23_P205098	PDS5B	185	306	316	405	364	471	341
A_24_P795594	ALG9	181	373	293	254	327	268	343
A_33_P3354464	LOXL1	181	104	208	180	170	131	140
A_23_P53193	SYTL2	181	110	363	239	403	223	245
A_24_P286935	ARL3	179	296	151	131	114	118	142
A_24_P398147	NEBL	178	108	320	204	285	264	213
A_33_P3419165	TFPT	176	292	268	231	193	162	204
A_33_P3393088	RAB6C-AS1	176	357	267	464	216	353	193
A_33_P3228072	NPHP3	174	271	222	243	310	294	232
A_23_P339053		172	598	157	695	205	800	241
A_23_P91350	GPCPD1	168	290	203	223	199	198	163
A_23_P208400	ADCK4	168	102	169	137	154	156	162
A_21_P0013518	GYG1	167	261	366	488	274	312	324
A_23_P125596	RPS6KA6	162	289	183	254	108	170	151
A_33_P3243717	ZNF248	157	269	166	182	214	225	192
A_23_P21425	GPR119	153	250	120	142	113	153	118
A_21_P0010748	SEPT14	145	238	132	150	141	174	144
A_23_P153855	ZNF492	139	241	175	172	184	172	198
A_24_P191664	GOLIM4	129	371	124	277	191	383	133
A_24_P68819	PPP1R21	128	219	130	122	101	123	127
A_23_P208198	ZNF577	124	228	148	181	181	232	165
A_24_P349117	GPR158	123	207	232	376	214	250	269
A_24_P926783	ZNF880	121	209	117	122	137	110	123
A_23_P69179	LEPREL1	120	214	304	319	625	639	515
A_23_P315836	BAIAP2	118	345	128	139	144	120	147
A_21_P0014276		118	195	151	265	148	327	167
A_23_P147805	UPP1	118	229	153	284	121	250	105
A_23_P118042	LRRC36	115	232	217	454	246	403	176
A_33_P3307955	ABCD3	114	230	231	639	162	353	207
A_24_P379165	FOXO4	112	184	131	150	123	149	104
A_24_P37589	ACPP	107	187	114	139	122	123	116
A_19_P00319646	LINC00673	102	192	154	258	213	269	149
A_21_P0008578	LOC101928725	101	237	165	174	206	184	175

GeneSymbol	siCYP27B1_siVDR_25D3_signal	siCT_1,25D3/siCT_V1_Zscore	siCT_1,25D3/siCT_V1_ratio	siCT_25D3/siCT_V2_Zscore	siCT_25D3/siCT_V2_ratio	siCYP27B1_25D3/siCYP27B1_V_Zscore	siCYP27B1_25D3/siCYP27B1_ratio	siCYP27B1_siVDR_25D3/siCYP27B1_siVDR_V_Zscore	siCYP27B1_siVDR_25D3/siCYP27B1_siVDR_V_ratio	siCYP27B1_25/V/siCT_25D3/V2_ratio	siCYP27B1_siVDR_25D3/V/siCYP27B1_25D3/V_ratio	siC27B1_25D3/V/siCT_1,25D3/V1_ratio	siCT_V1_flag	siCT_1,25D3_flag	siCT_V2_flag	siCT_25D3_flag	siCYP27B1_V_flag	siCYP27B1_25D3_flag	siCYP27B1_siVDR_V_flag	siCYP27B1_siVDR_25D3_flag	PrimaryAccession
GDF15	93882	12.33	2.69	9.24	1.90	8.54	1.80	5.54	1.54	0.95	0.85	0.67	P	P	P	P	P	P	P	P	NM_004864
CLDN4	45207	5.47	1.55	5.94	1.60	6.16	1.69	2.15	1.18	1.06	0.70	1.09	P	P	P	P	P	P	P	P	NM_001305
MT2A	24943	-11.66	0.27	-7.40	0.48	-1.81	0.86	-3.83	0.74	1.80	0.87	3.13	P	P	P	P	P	P	P	P	NM_005953
ATP1B1	13470	-4.29	0.62	-3.12	0.73	-3.52	0.69	-0.26	0.95	0.94	1.38	1.11	P	P	P	P	P	P	P	P	NM_001677
OASL	409	4.32	1.61	3.10	1.50	1.03	1.15	1.21	1.14	0.77	0.99	0.71	P	P	P	P	P	P	P	P	NM_003733
SLC45A3	8784	3.68	1.50	5.87	1.79	5.98	1.87	0.69	1.13	1.04	0.60	1.24	P	P	P	P	P	P	P	P	NM_033102
UGT2B11	5752	-5.88	0.52	-2.18	0.75	-3.60	0.61	-1.28	0.92	0.81	1.51	1.17	P	P	P	P	P	P	P	P	NM_001073
MT1G	6027	-11.83	0.19	-5.96	0.46	-2.27	0.79	-2.16	0.68	1.71	0.86	4.07	P	P	P	P	P	P	P	P	NM_005950
UGT2B10	5511	-5.22	0.56	-1.49	0.82	-3.11	0.65	-1.88	0.89	0.79	1.36	1.17	P	P	P	P	P	P	P	P	NM_001075
MAFB	17917	4.32	1.61	4.48	1.56	4.53	1.47	0.48	1.09	0.94	0.74	0.91	P	P	P	P	P	P	P	P	NM_005461
ZBTB43	6807	4.23	1.60	3.04	1.35	1.44	1.16	0.93	1.18	0.86	1.01	0.73	P	P	P	P	P	P	P	P	NM_014007
MT1L	4653	-10.70	0.23	-5.45	0.49	-1.78	0.83	-5.14	0.73	1.69	0.88	3.66	P	P	P	P	P	P	P	P	NR_001447
CAMK2N1	29821	4.56	1.66	2.42	1.27	0.97	1.09	1.07	1.09	0.86	1.00	0.66	P	P	P	P	P	P	P	P	NM_018584
MT1B	5245	-9.86	0.25	-5.64	0.48	-1.81	0.83	-1.94	0.70	1.72	0.85	3.25	P	P	P	P	P	P	P	P	NM_005947
ST3GAL1	14212	5.98	1.94	4.00	1.49	3.11	1.38	1.03	1.20	0.93	0.87	0.71	P	P	P	P	P	P	P	P	NM_003033
AR	12200	8.93	2.69	10.26	2.78	10.18	2.90	2.00	1.43	1.05	0.49	1.08	P	P	P	P	P	P	P	P	NM_000044
MT1HL1	4742	-8.73	0.30	-5.07	0.52	-1.58	0.85	-5.06	0.73	1.64	0.86	2.85	P	P	P	P	P	P	P	P	NM_001276687
MT1E	3578	-10.60	0.23	-5.66	0.48	-1.14	0.86	-5.31	0.72	1.79	0.84	3.73	P	P	P	P	P	P	P	P	NM_175617
MT1X	3490	-7.55	0.35	-4.18	0.58	-0.79	0.90	-4.79	0.74	1.55	0.82	2.57	P	P	P	P	P	P	P	P	NM_005952
TRIB1	6993	6.94	2.16	7.50	2.11	7.24	2.13	6.94	1.54	1.01	0.72	0.99	P	P	P	P	P	P	P	P	NM_025195
STAP2	4569	4.15	1.58	4.65	1.84	4.18	1.55	0.37	1.02	0.84	0.66	0.98	P	P	P	P	P	P	P	P	NM_001013841
MAT2A	5608	4.55	1.66	5.63	1.75	3.68	1.47	0.33	1.02	0.84	0.70	0.89	P	P	P	P	P	P	P	P	NM_005911
TMEFF2	6633	2.98	1.51	3.04	1.35	0.71	1.08	0.03	1.00	0.80	0.93	0.71	P	P	P	P	P	P	P	P	NM_016192

Supplemental Table S6 Gene list of 1,25D3 target genes in siCT cells and 25D3 target genes in siCYP27B1 cells.

Supplemental Table S6A 1,25D3 target genes in siCT cells (76).

ProbeID	GeneSymbol	siCT_V1_signal	siCT_1,25D3_signal	siCT_V2_signal	siCT_25D3_signal	siCYP27B1_V_signal	siCYP27B1_25D3_signal	siCYP27B1_siVDR_V_signal	siCYP27B1_siVDR_25D3_signal	siCT_1,25D3/siCT_V1_Zscore	siCT_1,25D3/siCT_V1_ratio	siCT_25D3/siCT_V2_Zscore	siCT_25D3/siCT_V2_ratio	siCYP27B1_25D3/siCYP27B1_V_Zscore	siCYP27B1_25D3/siCYP27B1_ratio	siCYP27B1_siVDR_25D3/siCYP27B1_siVDR_V_Zscore	siCYP27B1_siVDR_25D3/siCYP27B1_siVDR_V_ratio	siCYP27B1_25/V/siCT_25D3/V2_ratio	siCYP27B1_siVDR_25D3/V/siCYP27B1_25D3/V_ratio	siC27B1_25D3/V/siCT_1,25D3/V1_ratio	siCT_V1_flag	siCT_1,25D3_flag	siCT_V2_flag	siCT_25D3_flag	siCYP27B1_V_flag	siCYP27B1_25D3_flag	siCYP27B1_siVDR_V_flag	siCYP27B1_siVDR_25D3_flag	PrimaryAccession
A_23_P16523	GDF15	40397	108529	141974	269139	102700	185340	60947	93882	12.33	2.69	9.24	1.90	8.54	1.80	5.54	1.54	0.95	0.85	0.67	P	P	P	P	P	P	P	P	NM_004864
A_23_P106844	MT2A	27944	7668	29692	14177	30083	25807	33511	24943	-11.66	0.27	-7.40	0.48	-1.81	0.86	-3.83	0.74	1.80	0.87	3.13	P	P	P	P	P	P	P	P	NM_005953
A_33_P3233645	MT1G	10806	2088	6493	2983	9896	7785	8926	6027	-11.83	0.19	-5.96	0.46	-2.27	0.79	-2.16	0.68	1.71	0.86	4.07	P	P	P	P	P	P	P	P	NM_005950
A_23_P427703	MT1L	8823	1995	5265	2586	6471	5358	6418	4653	-10.70	0.23	-5.45	0.49	-1.78	0.83	-5.14	0.73	1.69	0.88	3.66	P	P	P	P	P	P	P	P	NR_001447
A_23_P37983	MT1B	8565	2176	6045	2895	7709	6365	7465	5245	-9.86	0.25	-5.64	0.48	-1.81	0.83	-1.94	0.70	1.72	0.85	3.25	P	P	P	P	P	P	P	P	NM_005947
A_23_P113111	AR	7199	19386	7131	19811	7680	22300	8545	12200	8.93	2.69	10.26	2.78	10.18	2.90	2.00	1.43	1.05	0.49	1.08	P	P	P	P	P	P	P	P	NM_000044
A_33_P3315314	MT1HL1	7062	2098	5552	2867	7058	5971	6508	4742	-8.73	0.30	-5.07	0.52	-1.58	0.85	-5.06	0.73	1.64	0.86	2.85	P	P	P	P	P	P	P	P	NM_001276687
A_23_P206724	MT1E	7058	1617	4099	1958	5511	4716	4990	3578	-10.60	0.23	-5.66	0.48	-1.14	0.86	-5.31	0.72	1.79	0.84	3.73	P	P	P	P	P	P	P	P	NM_175617
A_33_P3350056	MT1X	5959	2087	3706	2150	4575	4111	4709	3490	-7.55	0.35	-4.18	0.58	-0.79	0.90	-4.79	0.74	1.55	0.82	2.57	P	P	P	P	P	P	P	P	NM_005952
A_24_P252497	TRIB1	5552	11989	6847	14438	5608	11955	4526	6993	6.94	2.16	7.50	2.11	7.24	2.13	6.94	1.54	1.01	0.72	0.99	P	P	P	P	P	P	P	P	NM_025195
A_21_P0012331		4424	11175	2797	7283	1774	7627	2929	3406	8.36	2.53	7.32	2.60	10.65	4.30	2.40	1.16	1.65	0.27	1.70	P	P	P	P	P	P	P	P	ENST00000546734
A_23_P87560	BTG1	3886	9903	3652	8522	6035	9194	5010	6221	8.44	2.55	6.48	2.33	4.03	1.52	3.45	1.24	0.65	0.82	0.60	P	P	P	P	P	P	P	P	NM_001731
A_33_P3237634	TSC22D3	3595	7923	3169	7926	2443	6807	1953	2526	5.69	2.20	7.01	2.50	7.48	2.79	4.11	1.29	1.11	0.46	1.26	P	P	P	P	P	P	P	P	NM_004089
A_21_P0012330		3582	9087	2722	7522	1818	7421	2916	3540	6.70	2.54	7.78	2.76	10.27	4.08	3.09	1.21	1.48	0.30	1.61	P	P	P	P	P	P	P	P	ENST00000546734
A_23_P15174	MT1F	3336	812	1243	532	2966	1733	1724	1077	-10.17	0.24	-6.22	0.43	-3.93	0.58	-6.06	0.62	1.37	1.07	2.40	P	P	P	P	P	P	P	P	NM_005949
A_23_P57856	BCL6	2577	6016	3369	7683	2704	5870	2519	3373	6.10	2.33	6.31	2.28	5.66	2.17	4.65	1.34	0.95	0.62	0.93	P	P	P	P	P	P	P	P	NM_001130845
A_23_P25150	HOXC9	2489	6931	2633	6541	2942	6730	2514	3516	7.37	2.78	6.96	2.48	6.04	2.29	5.35	1.40	0.92	0.61	0.82	P	P	P	P	P	P	P	P	NM_006897
A_23_P138665	GLUD1	2311	4669	2458	5116	2405	4143	2427	2811	5.06	2.02	5.61	2.08	3.97	1.72	2.34	1.16	0.83	0.67	0.85	P	P	P	P	P	P	P	P	NM_005271
A_21_P0012332		2176	5874	1577	4326	917	3948	1762	1874	7.15	2.70	7.72	2.74	10.66	4.30	0.98	1.06	1.57	0.25	1.59	P	P	P	P	P	P	P	P	ENST00000453395
A_23_P110212	ACSL1	2142	4416	1343	2444	1589	2355	1861	2152	5.21	2.06	4.58	1.82	2.87	1.48	2.31	1.16	0.81	0.78	0.72	P	P	P	P	P	P	P	P	NM_001995
A_23_P103588	HMGCS2	1695	677	1957	939	1617	740	1153	920	-6.19	0.40	-5.38	0.48	-5.50	0.46	-2.91	0.80	0.95	1.74	1.15	P	P	P	P	P	P	P	P	NM_005518
A_23_P136671	UGT2B7	1601	560	344	176	331	161	458	323	-7.07	0.35	-3.88	0.51	-3.61	0.49	-3.21	0.71	0.95	1.45	1.39	P	P	P	P	P	P	P	P	NM_001074
A_23_P152055	EFTUD1	1498	3669	1936	4639	2011	4493	1939	2260	6.45	2.45	6.69	2.40	5.87	2.23	2.44	1.17	0.93	0.52	0.91	P	P	P	P	P	P	P	P	NM_024580
A_23_P29067	TMPRSS2	900	21619	469	11703	478	8022	430	1637	22.88	24.01	24.63	24.96	20.59	16.77	17.24	3.81	0.67	0.23	0.70	P	P	P	P	P	P	P	P	NM_005656
A_24_P46953	SGK3	893	323	484	470	470	469	578	591	-6.86	0.36	-0.21	0.97	0.00	1.00	0.29	1.02	1.03	1.02	2.76	P	P	P	P	P	P	P	P	NM_013257
A_23_P210581	KCNG1	875	2016	491	1035	876	1671	884	1149	5.62	2.30	5.47	2.11	4.57	1.91	3.39	1.30	0.90	0.68	0.83	P	P	P	P	P	P	P	P	NM_002237
A_23_P31407	AGR2	764	294	268	134	278	156	350	296	-6.44	0.38	-3.99	0.50	-2.92	0.56	-1.53	0.85	1.11	1.52	1.45	P	P	P	P	P	P	P	P	NM_006408
A_33_P3292028	DPP9	759	1563	769	1352	681	1214	759	862	4.85	2.06	4.14	1.76	4.09	1.78	1.65	1.14	1.01	0.64	0.87	P	P	P	P	P	P	P	P	NM_139159
A_23_P65307	SLITRK6	758	333	1314	660	1265	752	1200	1055	-5.55	0.44	-5.04	0.50	-3.66	0.59	-1.65	0.88	1.18	1.48	1.35	P	P	P	P	P	P	P	P	NM_032229
A_23_P93269	ZNF165	750	2237	828	2020	914	2085	917	1026	7.36	2.98	6.55	2.44	6.02	2.28	1.45	1.12	0.93	0.49	0.76	P	P	P	P	P	P	P	P	NM_003447
A_23_P139418	GALNT18	689	2079	1218	3488	1373	3558	1139	1520	7.43	3.02	8.05	2.86	6.95	2.59	3.72	1.33	0.91	0.51	0.86	P	P	P	P	P	P	P	P	NM_198516
A_21_P0012258	MAD1L1	678	2240	528	1145	464	1082	426	555	8.04	3.30	5.68	2.17	5.98	2.33	3.42	1.30	1.08	0.56	0.71	P	P	P	P	P	P	P	P	NM_003550
A_33_P3569068	MAD1L1	672	2182	549	1140	443	1108	431	591	7.92	3.25	5.36	2.08	6.48	2.50	4.09	1.37	1.20	0.55	0.77	P	P	P	P	P	P	P	P	NM_003550
A_23_P320578	RGS16	644	318	611	471	743	594	652	539	-4.75	0.49	-1.90	0.77	-1.56	0.80	-2.44	0.83	1.04	1.03	1.62	P	P	P	P	P	P	P	P	NM_002928
A_21_P0011196		596	1447	408	1095	248	954	405	493	5.97	2.43	7.24	2.68	9.52	3.85	2.54	1.22	1.43	0.32	1.59	P	P	P	P	P	P	P	P	ENST00000550892
A_21_P0000925	LINC01341	572	1399	419	1080	384	1024	266	343	6.02	2.45	6.95	2.58	6.92	2.66	2.36	1.29	1.03	0.48	1.09	P	P	P	P	P	P	P	P	AK022825
A_23_P128215	SOCS2	570	283	806	488	1049	539	938	875	-3.17	0.50	-3.67	0.61	-4.68	0.51	-0.89	0.93	0.85	1.81	1.04	P	P	P	P	P	P	P	P	NM_003877
A_21_P0000924	LINC01341	565	1390	464	1199	409	1018	268	363	6.06	2.46	6.96	2.58	6.44	2.49	2.82	1.36	0.96	0.55	1.01	P	P	P	P	P	P	P	P	AK022825
A_23_P64808	HOXC13	534	1405	450	1150	570	1181	421	538	6.51	2.63	6.89	2.56	5.16	2.07	3.18	1.28	0.81	0.62	0.79	P	P	P	P	P	P	P	P	NM_017410
A_33_P3231156		499	149	179	107	215	205	235	186	-5.48	0.30	-2.96	0.60	-0.24	0.95	-2.16	0.79	1.59	0.83	3.20	P	P	P	P	P	P	P	P	
A_23_P23815	SLC30A1	489	228	501	305	614	609	529	471	-3.44	0.47	-2.87	0.61	-0.04	0.99	-1.49	0.89	1.63	0.90	2.12	P	P	P	P	P	P	P	P	NM_021194
A_23_P409623	PPFIBP2	464	1182	319	691	281	609	276	321	6.30	2.55	5.67	2.17	5.46	2.16	1.41	1.16	1.00	0.54	0.85	P	P	P	P	P	P	P	P	NM_003621
A_33_P3258223	MCM7	453	142	326	238	213	208	346	311	-5.25	0.31	-1.83	0.73	-0.11	0.98	-0.98	0.90	1.34	0.92	3.12	P	P	P	P	P	P	P	P	NM_005916
A_23_P105562	VWF	445	915	555	1012	313	417	457	507	4.85	2.06	4.40	1.82	2.03	1.33	1.35	1.11	0.73	0.83	0.65	P	P	P	P	P	P	P	P	NM_000552
A_23_P402610	PFAS	438	203	211	214	142	186	260	221	-3.47	0.46	0.09	1.02	1.37	1.31	-1.49	0.85	1.29	0.65	2.82	P	P	P	P	P	P	P	P	NM_012393
A_21_P0010925		422	129	167	185	139	154	183	163	-5.38	0.30	0.62	1.11	0.52	1.11	-1.09	0.89	0.99	0.80	3.63	P	P	P	P	P	P	P	P	ENST00000439559
A_23_P500353	KCNN2	401	4810	388	6010	660	8110	689	3314	16.73	12.00	20.09	15.50	18.32	12.29	20.25	4.81	0.79	0.39	1.02	P	P	P	P	P	P	P	P	NM_021614
A_32_P19840	LOC100507507	343	976	704	1416	721	2176	435	607	7.04	2.85	5.13	2.01	7.80	3.02	4.30	1.40	1.50	0.46	1.06	P	P	P	P	P	P	P	P	XM_006710194
A_24_P921897	HOOK1	341	1062	282	850	315	966	236	318	7.64	3.11	8.08	3.01	7.91	3.07	2.77	1.35	1.02	0.44	0.98	P	P	P	P	P	P	P	P	NM_015888
A_21_P0000053	C16orf52	331	911	433	1310	556	1667	514	591	6.80	2.75	8.11	3.02	7.76	3.00	1.82	1.15	0.99	0.38	1.09	P	P	P	P	P	P	P	P	NM_001164579
A_32_P78783	ZNF778	321	114	139	160	148	148	145	139	-4.68	0.35	0.82	1.15	0.01	1.00	-0.43	0.95	0.87	0.95	2.82	P	P	P	P	P	P	P	P	NM_001201407
A_33_P3409844	HOOK1	301	706	177	430	190	461	175	160	5.74	2.35	5.13	2.42	4.47	2.42	-0.85	0.91	1.00	0.38	1.03	P	P	P	P	P	P	P	P	NM_015888
A_33_P3414669	RLIM	280	112	162	150	162	168	190	185	-4.13	0.40	-0.42	0.93	0.19	1.04	-0.24	0.97	1.11	0.94	2.58	P	P	P	P	P	P	P	P	NM_183353
A_23_P169003	SH2D4A	278	649	259	524	181	350	199	252	5.69	2.33	4.08	2.02	3.35	1.94	2.20	1.27	0.96	0.65	0.83	P	P	P	P	P	P	P	P	NM_022071
A_23_P126836	TNFSF4	274	779	355	860	158	357	192	255	7.04	2.85	6.48	2.42	4.13	2.26	2.64	1.33	0.94	0.59	0.79	P	P	P	P	P	P	P	P	NM_003326
A_23_P71170	TRPV6	270	1294	398	2149	499	2375	342	570	10.54	4.79	12.37	5.40	11.02	4.76	6.60	1.67	0.88	0.35	0.99	P	P	P	P	P	P	P	P	NM_018646
A_33_P3246985	PDE4DIP	262	120	312	214	267	172	366	340	-3.53	0.46	-2.18	0.69	-2.20	0.64	-0.67	0.93	0.94	1.44	1.40	P	P	P	P	P	P	P	P	NM_022359
A_33_P3364854	TRPV6	261	1250	286	1814	399	2066	301	432	10.55	4.79	13.56	6.35	11.61	5.18	3.35	1.44	0.82	0.28	1.08	P	P	P	P	P	P	P	P	NM_018646
A_33_P3250953	SLC35B4	248	529	283	524	248	426	257	323	3.42	2.13	3.56	1.85	2.74	1.72	2.11	1.26	0.93	0.73	0.81	P	P	P	P	P	P	P	P	NM_032826
A_33_P3333982	PDE4DIP	241	104	526	323	330	201	371	300	-3.79	0.43	-2.82	0.61	-2.47	0.61	-1.94	0.81	0.99	1.33	1.41	P	P	P	P	P	P	P	P	NM_022359
A_33_P3360675	PRKAR1B	235	115	156	149	129	122	154	152	-3.24	0.49	-0.25	0.96	-0.25	0.95	-0.14	0.98	0.99	1.04	1.94	P	P	P	P	P	P	P	P	NM_001164761
A_23_P170733	ANTXR2	232	104	152	123	350	316	246	228	-3.63	0.45	-1.23	0.81	-0.50	0.90	-0.69	0.93	1.12	1.03	2.02	P	P	P	P	P	P	P	P	NM_058172
A_23_P19624	BMP6	230	477	176	905	389	1473	155	175	3.31	2.08	9.47	5.13	9.40	3.79	1.14	1.13	0.74	0.30	1.82	P	P	P	P	P	P	P	P	NM_001718
A_21_P0011335	EFTUD1	223	545	197	425	163	405	158	171	4.04	2.44	4.47	2.16	4.59	2.48	0.76	1.09	1.15	0.44	1.02	P	P	P	P	P	P	P	P	NM_024580
A_21_P0012089	FAR2P1	213	518	278	585	183	382	138	223	4.01	2.43	4.31	2.10	3.73	2.09	4.44	1.62	0.99	0.77	0.86	P	P	P	P	P	P	P	P	NR_026758
A_24_P140608	HBEGF	202	427	248	624	154	370	137	177	3.38	2.11	5.36	2.52	4.43	2.40	2.41	1.30	0.95	0.54	1.14	P	P	P	P	P	P	P	P	NM_001945
A_23_P213085	SLC10A7	193	413	161	245	124	159	106	137	3.45	2.15	2.46	1.53	1.28	1.29	2.37	1.29	0.84	1.01	0.60	P	P	P	P	P	P	P	P	NM_001029998
A_23_P119778	SLC39A10	189	540	174	283	163	181	163	180	4.75	2.86	2.80	1.62	0.53	1.11	0.88	1.10	0.68	0.99	0.39	P	P	P	P	P	P	P	P	NM_020342
A_24_P795594	ALG9	181	373	293	254	327	268	343	327	3.25	2.05	-0.83	0.87	-0.99	0.82	-0.42	0.95	0.95	1.16	0.40	P	P	P	P	P	P	P	P	NM_001077691
A_33_P3393088	RAB6C-AS1	176	357	267	464	216	353	193	206	3.21	2.03	3.21	1.74	2.48	1.63	0.62	1.07	0.94	0.65	0.80	P	P	P	P	P	P	P	P	BC044241
A_23_P339053		172	598	157	695	205	800	241	274	5.63	3.48	8.63	4.43	9.61	3.90	1.17	1.13	0.88	0.29	1.12	P	P	P	P	P	P	P	P	ENST00000569656
A_24_P191664	GOLIM4	129	371	124	277	191	383	133	199	4.77	2.88	4.65	2.23	3.52	2.00	3.67	1.49	0.90	0.74	0.70	P	P	P	P	P	P	P	P	NM_014498
A_23_P315836	BAIAP2	118	345	128	139	144	120	147	131	4.83	2.92	0.46	1.08	-0.91	0.83	-1.05	0.89	0.77	1.07	0.29	P	P	P	P	P	P	P	P	NM_017451
A_23_P118042	LRRC36	115	232	217	454	246	403	176	207	3.19	2.02	4.28	2.09	2.49	1.64	1.52	1.18	0.78	0.72	0.81	P	P	P	P	P	P	P	P	NM_018296
A_33_P3307955	ABCD3	114	230	231	639	162	353	207	254	3.19	2.02	5.91	2.77	3.94	2.18	1.90	1.23	0.79	0.56	1.08	P	P	P	P	P	P	P	P	NM_001122674
A_21_P0008578	LOC101928725	101	237	165	174	206	184	175	171	3.85	2.34	0.30	1.05	-0.56	0.89	-0.21	0.98	0.85	1.09	0.38	P	P	P	P	P	P	P	P	ENST00000500929

Supplemental Table S6B 25D3 target genes in siCYP27B1 cells (46).

ProbeID	GeneSymbol	siCT_V1_signal	siCT_1,25D3_signal	siCT_V2_signal	siCT_25D3_signal	siCYP27B1_V_signal	siCYP27B1_25D3_signal	siCYP27B1_siVDR_V_signal	siCYP27B1_siVDR_25D3_signal	siCT_1,25D3/siCT_V1_Zscore	siCT_1,25D3/siCT_V1_ratio	siCT_25D3/siCT_V2_Zscore	siCT_25D3/siCT_V2_ratio	siCYP27B1_25D3/siCYP27B1_V_Zscore	siCYP27B1_25D3/siCYP27B1_ratio	siCYP27B1_siVDR_25D3/siCYP27B1_siVDR_V_Zscore	siCYP27B1_siVDR_25D3/siCYP27B1_siVDR_V_ratio	siCYP27B1_25/V/siCT_25D3/V2_ratio	siCYP27B1_siVDR_25D3/V/siCYP27B1_25D3/V_ratio	siC27B1_25D3/V/siCT_1,25D3/V1_ratio	siCT_V1_flag	siCT_1,25D3_flag	siCT_V2_flag	siCT_25D3_flag	siCYP27B1_V_flag	siCYP27B1_25D3_flag	siCYP27B1_siVDR_V_flag	siCYP27B1_siVDR_25D3_flag	PrimaryAccession
A_33_P3368785	DLGAP1-AS1	913	1562	992	2083	1046	2120	721	1002	3.61	1.71	5.44	2.10	5.16	2.03	4.24	1.39	0.97	0.68	1.19	P	P	P	P	P	P	P	P	NR_024101
A_23_P29067	TMPRSS2	900	21619	469	11703	478	8022	430	1637	22.88	24.01	24.63	24.96	20.59	16.77	17.24	3.81	0.67	0.23	0.70	P	P	P	P	P	P	P	P	NM_005656
A_23_P93269	ZNF165	750	2237	828	2020	914	2085	917	1026	7.36	2.98	6.55	2.44	6.02	2.28	1.45	1.12	0.93	0.49	0.76	P	P	P	P	P	P	P	P	NM_003447
A_23_P139418	GALNT18	689	2079	1218	3488	1373	3558	1139	1520	7.43	3.02	8.05	2.86	6.95	2.59	3.72	1.33	0.91	0.51	0.86	P	P	P	P	P	P	P	P	NM_198516
A_21_P0012258	MAD1L1	678	2240	528	1145	464	1082	426	555	8.04	3.30	5.68	2.17	5.98	2.33	3.42	1.30	1.08	0.56	0.71	P	P	P	P	P	P	P	P	NM_003550
A_33_P3569068	MAD1L1	672	2182	549	1140	443	1108	431	591	7.92	3.25	5.36	2.08	6.48	2.50	4.09	1.37	1.20	0.55	0.77	P	P	P	P	P	P	P	P	NM_003550
A_21_P0011196		596	1447	408	1095	248	954	405	493	5.97	2.43	7.24	2.68	9.52	3.85	2.54	1.22	1.43	0.32	1.59	P	P	P	P	P	P	P	P	ENST00000550892
A_23_P254888	ZYX	580	1034	673	1072	575	1186	467	552	3.89	1.78	3.42	1.59	5.12	2.06	2.18	1.18	1.29	0.57	1.16	P	P	P	P	P	P	P	P	NM_003461
A_21_P0000925	LINC01341	572	1399	419	1080	384	1024	266	343	6.02	2.45	6.95	2.58	6.92	2.66	2.36	1.29	1.03	0.48	1.09	P	P	P	P	P	P	P	P	AK022825
A_23_P34093	G6PD	567	897	847	2130	740	1958	1006	1338	3.09	1.58	6.77	2.52	6.88	2.65	3.68	1.33	1.05	0.50	1.67	P	P	P	P	P	P	P	P	NM_000402
A_21_P0000924	LINC01341	565	1390	464	1199	409	1018	268	363	6.06	2.46	6.96	2.58	6.44	2.49	2.82	1.36	0.96	0.55	1.01	P	P	P	P	P	P	P	P	AK022825
A_23_P64808	HOXC13	534	1405	450	1150	570	1181	421	538	6.51	2.63	6.89	2.56	5.16	2.07	3.18	1.28	0.81	0.62	0.79	P	P	P	P	P	P	P	P	NM_017410
A_23_P105002	ROM1	517	963	887	2156	679	2924	769	1056	4.19	1.86	6.52	2.43	10.66	4.30	4.09	1.37	1.77	0.32	2.31	P	P	P	P	P	P	P	P	NM_000327
A_23_P409623	PPFIBP2	464	1182	319	691	281	609	276	321	6.30	2.55	5.67	2.17	5.46	2.16	1.41	1.16	1.00	0.54	0.85	P	P	P	P	P	P	P	P	NM_003621
A_23_P217755	SHROOM2	444	724	243	600	256	628	232	331	3.29	1.63	5.25	2.48	6.34	2.45	3.31	1.43	0.99	0.58	1.50	P	P	P	P	P	P	P	P	NM_001649
A_23_P103756	OVGP1	440	226	413	192	445	205	403	309	-3.02	0.51	-4.41	0.47	-3.89	0.46	-2.45	0.77	0.99	1.67	0.90	P	P	P	P	P	P	P	P	NM_002557
A_23_P500353	KCNN2	401	4810	388	6010	660	8110	689	3314	16.73	12.00	20.09	15.50	18.32	12.29	20.25	4.81	0.79	0.39	1.02	P	P	P	P	P	P	P	P	NM_021614
A_32_P19840	LOC100507507	343	976	704	1416	721	2176	435	607	7.04	2.85	5.13	2.01	7.80	3.02	4.30	1.40	1.50	0.46	1.06	P	P	P	P	P	P	P	P	XM_006710194
A_24_P921897	HOOK1	341	1062	282	850	315	966	236	318	7.64	3.11	8.08	3.01	7.91	3.07	2.77	1.35	1.02	0.44	0.98	P	P	P	P	P	P	P	P	NM_015888
A_21_P0000053	C16orf52	331	911	433	1310	556	1667	514	591	6.80	2.75	8.11	3.02	7.76	3.00	1.82	1.15	0.99	0.38	1.09	P	P	P	P	P	P	P	P	NM_001164579
A_33_P3409844	HOOK1	301	706	177	430	190	461	175	160	5.74	2.35	5.13	2.42	4.47	2.42	-0.85	0.91	1.00	0.38	1.03	P	P	P	P	P	P	P	P	NM_015888
A_33_P3369899	TMEM158	275	170	334	173	552	250	369	301	-2.16	0.62	-3.80	0.52	-3.58	0.45	-1.89	0.81	0.87	1.80	0.73	P	P	P	P	P	P	P	P	NM_015444
A_23_P126836	TNFSF4	274	779	355	860	158	357	192	255	7.04	2.85	6.48	2.42	4.13	2.26	2.64	1.33	0.94	0.59	0.79	P	P	P	P	P	P	P	P	NM_003326
A_23_P71170	TRPV6	270	1294	398	2149	499	2375	342	570	10.54	4.79	12.37	5.40	11.02	4.76	6.60	1.67	0.88	0.35	0.99	P	P	P	P	P	P	P	P	NM_018646
A_33_P3364854	TRPV6	261	1250	286	1814	399	2066	301	432	10.55	4.79	13.56	6.35	11.61	5.18	3.35	1.44	0.82	0.28	1.08	P	P	P	P	P	P	P	P	NM_018646
A_33_P131899	SDCBP2	233	126	473	165	2093	960	533	402	-2.79	0.54	-6.11	0.35	-5.70	0.46	-3.63	0.75	1.32	1.65	0.85	P	P	P	P	P	P	P	P	NM_080489
A_23_P19624	BMP6	230	477	176	905	389	1473	155	175	3.31	2.08	9.47	5.13	9.40	3.79	1.14	1.13	0.74	0.30	1.82	P	P	P	P	P	P	P	P	NM_001718
A_21_P0011335	EFTUD1	223	545	197	425	163	405	158	171	4.04	2.44	4.47	2.16	4.59	2.48	0.76	1.09	1.15	0.44	1.02	P	P	P	P	P	P	P	P	NM_024580
A_21_P0012089	FAR2P1	213	518	278	585	183	382	138	223	4.01	2.43	4.31	2.10	3.73	2.09	4.44	1.62	0.99	0.77	0.86	P	P	P	P	P	P	P	P	NR_026758
A_24_P140608	HBEGF	202	427	248	624	154	370	137	177	3.38	2.11	5.36	2.52	4.43	2.40	2.41	1.30	0.95	0.54	1.14	P	P	P	P	P	P	P	P	NM_001945
A_23_P339053		172	598	157	695	205	800	241	274	5.63	3.48	8.63	4.43	9.61	3.90	1.17	1.13	0.88	0.29	1.12	P	P	P	P	P	P	P	P	ENST00000569656
A_24_P191664	GOLIM4	129	371	124	277	191	383	133	199	4.77	2.88	4.65	2.23	3.52	2.00	3.67	1.49	0.90	0.74	0.70	P	P	P	P	P	P	P	P	NM_014498
A_21_P0014276		118	195	151	265	148	327	167	206	2.25	1.65	3.27	1.76	4.01	2.21	1.96	1.24	1.26	0.56	1.34	P	P	P	P	P	P	P	P	
A_23_P147805	UPP1	118	229	153	284	121	250	105	111	3.00	1.94	3.60	1.86	3.68	2.07	0.21	1.06	1.11	0.51	1.07	P	P	P	P	P	P	P	P	NM_001287428
A_33_P3307955	ABCD3	114	230	231	639	162	353	207	254	3.19	2.02	5.91	2.77	3.94	2.18	1.90	1.23	0.79	0.56	1.08	P	P	P	P	P	P	P	P	NM_001122674

Supplemental Table S6C Common target genes between 1,25D3 in siCT and 25D3 in siCYP27B1 (36).

ProbeID	GeneSymbol	siCT_V1_signal	siCT_1,25D3_signal	siCT_V2_signal	siCT_25D3_signal	siCYP27B1_V_signal	siCYP27B1_25D3_signal	siCYP27B1_siVDR_V_signal	siCYP27B1_siVDR_25D3_signal	siCT_1,25D3/siCT_V1_Zscore	siCT_1,25D3/siCT_V1_ratio	siCT_25D3/siCT_V2_Zscore	siCT_25D3/siCT_V2_ratio	siCYP27B1_25D3/siCYP27B1_V_Zscore	siCYP27B1_25D3/siCYP27B1_ratio	siCYP27B1_siVDR_25D3/siCYP27B1_siVDR_V_Zscore	siCYP27B1_siVDR_25D3/siCYP27B1_siVDR_V_ratio	siCYP27B1_25/V/siCT_25D3/V2_ratio	siCYP27B1_siVDR_25D3/V/siCYP27B1_25D3/V_ratio	siC27B1_25D3/V/siCT_1,25D3/V1_ratio	siCT_V1_flag	siCT_1,25D3_flag	siCT_V2_flag	siCT_25D3_flag	siCYP27B1_V_flag	siCYP27B1_25D3_flag	siCYP27B1_siVDR_V_flag	siCYP27B1_siVDR_25D3_flag	PrimaryAccession
A_23_P113111	AR	7199	19386	7131	19811	7680	22300	8545	12200	8.93	2.69	10.26	2.78	10.18	2.90	2.00	1.43	1.05	0.49	1.08	P	P	P	P	P	P	P	P	NM_000044
A_24_P252497	TRIB1	5552	11989	6847	14438	5608	11955	4526	6993	6.94	2.16	7.50	2.11	7.24	2.13	6.94	1.54	1.01	0.72	0.99	P	P	P	P	P	P	P	P	NM_025195
A_21_P0012331		4424	11175	2797	7283	1774	7627	2929	3406	8.36	2.53	7.32	2.60	10.65	4.30	2.40	1.16	1.65	0.27	1.70	P	P	P	P	P	P	P	P	ENST00000546734
A_33_P3237634	TSC22D3	3595	7923	3169	7926	2443	6807	1953	2526	5.69	2.20	7.01	2.50	7.48	2.79	4.11	1.29	1.11	0.46	1.26	P	P	P	P	P	P	P	P	NM_004089
A_21_P0012330		3582	9087	2722	7522	1818	7421	2916	3540	6.70	2.54	7.78	2.76	10.27	4.08	3.09	1.21	1.48	0.30	1.61	P	P	P	P	P	P	P	P	ENST00000546734
A_23_P57856	BCL6	2577	6016	3369	7683	2704	5870	2519	3373	6.10	2.33	6.31	2.28	5.66	2.17	4.65	1.34	0.95	0.62	0.93	P	P	P	P	P	P	P	P	NM_001130845
A_23_P25150	HOXC9	2489	6931	2633	6541	2942	6730	2514	3516	7.37	2.78	6.96	2.48	6.04	2.29	5.35	1.40	0.92	0.61	0.82	P	P	P	P	P	P	P	P	NM_006897
A_21_P0012332		2176	5874	1577	4326	917	3948	1762	1874	7.15	2.70	7.72	2.74	10.66	4.30	0.98	1.06	1.57	0.25	1.59	P	P	P	P	P	P	P	P	ENST00000453395
A_23_P103588	HMGCS2	1695	677	1957	939	1617	740	1153	920	-6.19	0.40	-5.38	0.48	-5.50	0.46	-2.91	0.80	0.95	1.74	1.15	P	P	P	P	P	P	P	P	NM_005518
A_23_P136671	UGT2B7	1601	560	344	176	331	161	458	323	-7.07	0.35	-3.88	0.51	-3.61	0.49	-3.21	0.71	0.95	1.45	1.39	P	P	P	P	P	P	P	P	NM_001074
A_23_P152055	EFTUD1	1498	3669	1936	4639	2011	4493	1939	2260	6.45	2.45	6.69	2.40	5.87	2.23	2.44	1.17	0.93	0.52	0.91	P	P	P	P	P	P	P	P	NM_024580
A_23_P29067	TMPRSS2	900	21619	469	11703	478	8022	430	1637	22.88	24.01	24.63	24.96	20.59	16.77	17.24	3.81	0.67	0.23	0.70	P	P	P	P	P	P	P	P	NM_005656
A_23_P93269	ZNF165	750	2237	828	2020	914	2085	917	1026	7.36	2.98	6.55	2.44	6.02	2.28	1.45	1.12	0.93	0.49	0.76	P	P	P	P	P	P	P	P	NM_003447
A_23_P139418	GALNT18	689	2079	1218	3488	1373	3558	1139	1520	7.43	3.02	8.05	2.86	6.95	2.59	3.72	1.33	0.91	0.51	0.86	P	P	P	P	P	P	P	P	NM_198516
A_21_P0012258	MAD1L1	678	2240	528	1145	464	1082	426	555	8.04	3.30	5.68	2.17	5.98	2.33	3.42	1.30	1.08	0.56	0.71	P	P	P	P	P	P	P	P	NM_003550
A_33_P3569068	MAD1L1	672	2182	549	1140	443	1108	431	591	7.92	3.25	5.36	2.08	6.48	2.50	4.09	1.37	1.20	0.55	0.77	P	P	P	P	P	P	P	P	NM_003550
A_21_P0011196		596	1447	408	1095	248	954	405	493	5.97	2.43	7.24	2.68	9.52	3.85	2.54	1.22	1.43	0.32	1.59	P	P	P	P	P	P	P	P	ENST00000550892
A_21_P0000925	LINC01341	572	1399	419	1080	384	1024	266	343	6.02	2.45	6.95	2.58	6.92	2.66	2.36	1.29	1.03	0.48	1.09	P	P	P	P	P	P	P	P	AK022825
A_21_P0000924	LINC01341	565	1390	464	1199	409	1018	268	363	6.06	2.46	6.96	2.58	6.44	2.49	2.82	1.36	0.96	0.55	1.01	P	P	P	P	P	P	P	P	AK022825
A_23_P64808	HOXC13	534	1405	450	1150	570	1181	421	538	6.51	2.63	6.89	2.56	5.16	2.07	3.18	1.28	0.81	0.62	0.79	P	P	P	P	P	P	P	P	NM_017410
A_23_P409623	PPFIBP2	464	1182	319	691	281	609	276	321	6.30	2.55	5.67	2.17	5.46	2.16	1.41	1.16	1.00	0.54	0.85	P	P	P	P	P	P	P	P	NM_003621
A_23_P500353	KCNN2	401	4810	388	6010	660	8110	689	3314	16.73	12.00	20.09	15.50	18.32	12.29	20.25	4.81	0.79	0.39	1.02	P	P	P	P	P	P	P	P	NM_021614
A_32_P19840	LOC100507507	343	976	704	1416	721	2176	435	607	7.04	2.85	5.13	2.01	7.80	3.02	4.30	1.40	1.50	0.46	1.06	P	P	P	P	P	P	P	P	XM_006710194
A_24_P921897	HOOK1	341	1062	282	850	315	966	236	318	7.64	3.11	8.08	3.01	7.91	3.07	2.77	1.35	1.02	0.44	0.98	P	P	P	P	P	P	P	P	NM_015888
A_21_P0000053	C16orf52	331	911	433	1310	556	1667	514	591	6.80	2.75	8.11	3.02	7.76	3.00	1.82	1.15	0.99	0.38	1.09	P	P	P	P	P	P	P	P	NM_001164579
A_33_P3409844	HOOK1	301	706	177	430	190	461	175	160	5.74	2.35	5.13	2.42	4.47	2.42	-0.85	0.91	1.00	0.38	1.03	P	P	P	P	P	P	P	P	NM_015888
A_23_P126836	TNFSF4	274	779	355	860	158	357	192	255	7.04	2.85	6.48	2.42	4.13	2.26	2.64	1.33	0.94	0.59	0.79	P	P	P	P	P	P	P	P	NM_003326
A_23_P71170	TRPV6	270	1294	398	2149	499	2375	342	570	10.54	4.79	12.37	5.40	11.02	4.76	6.60	1.67	0.88	0.35	0.99	P	P	P	P	P	P	P	P	NM_018646
A_33_P3364854	TRPV6	261	1250	286	1814	399	2066	301	432	10.55	4.79	13.56	6.35	11.61	5.18	3.35	1.44	0.82	0.28	1.08	P	P	P	P	P	P	P	P	NM_018646
A_23_P19624	BMP6	230	477	176	905	389	1473	155	175	3.31	2.08	9.47	5.13	9.40	3.79	1.14	1.13	0.74	0.30	1.82	P	P	P	P	P	P	P	P	NM_001718
A_21_P0011335	EFTUD1	223	545	197	425	163	405	158	171	4.04	2.44	4.47	2.16	4.59	2.48	0.76	1.09	1.15	0.44	1.02	P	P	P	P	P	P	P	P	NM_024580
A_21_P0012089	FAR2P1	213	518	278	585	183	382	138	223	4.01	2.43	4.31	2.10	3.73	2.09	4.44	1.62	0.99	0.77	0.86	P	P	P	P	P	P	P	P	NR_026758
A_24_P140608	HBEGF	202	427	248	624	154	370	137	177	3.38	2.11	5.36	2.52	4.43	2.40	2.41	1.30	0.95	0.54	1.14	P	P	P	P	P	P	P	P	NM_001945
A_23_P339053		172	598	157	695	205	800	241	274	5.63	3.48	8.63	4.43	9.61	3.90	1.17	1.13	0.88	0.29	1.12	P	P	P	P	P	P	P	P	ENST00000569656
A_24_P191664	GOLIM4	129	371	124	277	191	383	133	199	4.77	2.88	4.65	2.23	3.52	2.00	3.67	1.49	0.90	0.74	0.70	P	P	P	P	P	P	P	P	NM_014498
A_33_P3307955	ABCD3	114	230	231	639	162	353	207	254	3.19	2.02	5.91	2.77	3.94	2.18	1.90	1.23	0.79	0.56	1.08	P	P	P	P	P	P	P	P	NM_001122674

Supplemental Table S6D Specific target genes for 1,25D3 in siCT cells (40).

ProbeID	GeneSymbol	siCT_V1_signal	siCT_1,25D3_signal	siCT_V2_signal	siCT_25D3_signal	siCYP27B1_V_signal	siCYP27B1_25D3_signal	siCYP27B1_siVDR_V_signal	siCYP27B1_siVDR_25D3_signal	siCT_1,25D3/siCT_V1_Zscore	siCT_1,25D3/siCT_V1_ratio	siCT_25D3/siCT_V2_Zscore	siCT_25D3/siCT_V2_ratio	siCYP27B1_25D3/siCYP27B1_V_Zscore	siCYP27B1_25D3/siCYP27B1_ratio	siCYP27B1_siVDR_25D3/siCYP27B1_siVDR_V_Zscore	siCYP27B1_siVDR_25D3/siCYP27B1_siVDR_V_ratio	siCYP27B1_25/V/siCT_25D3/V2_ratio	siCYP27B1_siVDR_25D3/V/siCYP27B1_25D3/V_ratio	siC27B1_25D3/V/siCT_1,25D3/V1_ratio	siCT_V1_flag	siCT_1,25D3_flag	siCT_V2_flag	siCT_25D3_flag	siCYP27B1_V_flag	siCYP27B1_25D3_flag	siCYP27B1_siVDR_V_flag	siCYP27B1_siVDR_25D3_flag	PrimaryAccession
A_23_P16523	GDF15	40397	108529	141974	269139	102700	185340	60947	93882	12.33	2.69	9.24	1.90	8.54	1.80	5.54	1.54	0.95	0.85	0.67	P	P	P	P	P	P	P	P	NM_004864
A_23_P106844	MT2A	27944	7668	29692	14177	30083	25807	33511	24943	-11.66	0.27	-7.40	0.48	-1.81	0.86	-3.83	0.74	1.80	0.87	3.13	P	P	P	P	P	P	P	P	NM_005953
A_33_P3233645	MT1G	10806	2088	6493	2983	9896	7785	8926	6027	-11.83	0.19	-5.96	0.46	-2.27	0.79	-2.16	0.68	1.71	0.86	4.07	P	P	P	P	P	P	P	P	NM_005950
A_23_P427703	MT1L	8823	1995	5265	2586	6471	5358	6418	4653	-10.70	0.23	-5.45	0.49	-1.78	0.83	-5.14	0.73	1.69	0.88	3.66	P	P	P	P	P	P	P	P	NR_001447
A_23_P37983	MT1B	8565	2176	6045	2895	7709	6365	7465	5245	-9.86	0.25	-5.64	0.48	-1.81	0.83	-1.94	0.70	1.72	0.85	3.25	P	P	P	P	P	P	P	P	NM_005947
A_33_P3315314	MT1HL1	7062	2098	5552	2867	7058	5971	6508	4742	-8.73	0.30	-5.07	0.52	-1.58	0.85	-5.06	0.73	1.64	0.86	2.85	P	P	P	P	P	P	P	P	NM_001276687
A_23_P206724	MT1E	7058	1617	4099	1958	5511	4716	4990	3578	-10.60	0.23	-5.66	0.48	-1.14	0.86	-5.31	0.72	1.79	0.84	3.73	P	P	P	P	P	P	P	P	NM_175617
A_33_P3350056	MT1X	5959	2087	3706	2150	4575	4111	4709	3490	-7.55	0.35	-4.18	0.58	-0.79	0.90	-4.79	0.74	1.55	0.82	2.57	P	P	P	P	P	P	P	P	NM_005952
A_23_P87560	BTG1	3886	9903	3652	8522	6035	9194	5010	6221	8.44	2.55	6.48	2.33	4.03	1.52	3.45	1.24	0.65	0.82	0.60	P	P	P	P	P	P	P	P	NM_001731
A_23_P15174	MT1F	3336	812	1243	532	2966	1733	1724	1077	-10.17	0.24	-6.22	0.43	-3.93	0.58	-6.06	0.62	1.37	1.07	2.40	P	P	P	P	P	P	P	P	NM_005949
A_23_P138665	GLUD1	2311	4669	2458	5116	2405	4143	2427	2811	5.06	2.02	5.61	2.08	3.97	1.72	2.34	1.16	0.83	0.67	0.85	P	P	P	P	P	P	P	P	NM_005271
A_23_P110212	ACSL1	2142	4416	1343	2444	1589	2355	1861	2152	5.21	2.06	4.58	1.82	2.87	1.48	2.31	1.16	0.81	0.78	0.72	P	P	P	P	P	P	P	P	NM_001995
A_24_P46953	SGK3	893	323	484	470	470	469	578	591	-6.86	0.36	-0.21	0.97	0.00	1.00	0.29	1.02	1.03	1.02	2.76	P	P	P	P	P	P	P	P	NM_013257
A_23_P210581	KCNG1	875	2016	491	1035	876	1671	884	1149	5.62	2.30	5.47	2.11	4.57	1.91	3.39	1.30	0.90	0.68	0.83	P	P	P	P	P	P	P	P	NM_002237
A_23_P31407	AGR2	764	294	268	134	278	156	350	296	-6.44	0.38	-3.99	0.50	-2.92	0.56	-1.53	0.85	1.11	1.52	1.45	P	P	P	P	P	P	P	P	NM_006408
A_33_P3292028	DPP9	759	1563	769	1352	681	1214	759	862	4.85	2.06	4.14	1.76	4.09	1.78	1.65	1.14	1.01	0.64	0.87	P	P	P	P	P	P	P	P	NM_139159
A_23_P65307	SLITRK6	758	333	1314	660	1265	752	1200	1055	-5.55	0.44	-5.04	0.50	-3.66	0.59	-1.65	0.88	1.18	1.48	1.35	P	P	P	P	P	P	P	P	NM_032229
A_23_P320578	RGS16	644	318	611	471	743	594	652	539	-4.75	0.49	-1.90	0.77	-1.56	0.80	-2.44	0.83	1.04	1.03	1.62	P	P	P	P	P	P	P	P	NM_002928
A_23_P128215	SOCS2	570	283	806	488	1049	539	938	875	-3.17	0.50	-3.67	0.61	-4.68	0.51	-0.89	0.93	0.85	1.81	1.04	P	P	P	P	P	P	P	P	NM_003877
A_33_P3231156		499	149	179	107	215	205	235	186	-5.48	0.30	-2.96	0.60	-0.24	0.95	-2.16	0.79	1.59	0.83	3.20	P	P	P	P	P	P	P	P	
A_23_P23815	SLC30A1	489	228	501	305	614	609	529	471	-3.44	0.47	-2.87	0.61	-0.04	0.99	-1.49	0.89	1.63	0.90	2.12	P	P	P	P	P	P	P	P	NM_021194
A_33_P3258223	MCM7	453	142	326	238	213	208	346	311	-5.25	0.31	-1.83	0.73	-0.11	0.98	-0.98	0.90	1.34	0.92	3.12	P	P	P	P	P	P	P	P	NM_005916
A_23_P105562	VWF	445	915	555	1012	313	417	457	507	4.85	2.06	4.40	1.82	2.03	1.33	1.35	1.11	0.73	0.83	0.65	P	P	P	P	P	P	P	P	NM_000552
A_23_P402610	PFAS	438	203	211	214	142	186	260	221	-3.47	0.46	0.09	1.02	1.37	1.31	-1.49	0.85	1.29	0.65	2.82	P	P	P	P	P	P	P	P	NM_012393
A_21_P0010925		422	129	167	185	139	154	183	163	-5.38	0.30	0.62	1.11	0.52	1.11	-1.09	0.89	0.99	0.80	3.63	P	P	P	P	P	P	P	P	ENST00000439559
A_32_P78783	ZNF778	321	114	139	160	148	148	145	139	-4.68	0.35	0.82	1.15	0.01	1.00	-0.43	0.95	0.87	0.95	2.82	P	P	P	P	P	P	P	P	NM_001201407
A_33_P3414669	RLIM	280	112	162	150	162	168	190	185	-4.13	0.40	-0.42	0.93	0.19	1.04	-0.24	0.97	1.11	0.94	2.58	P	P	P	P	P	P	P	P	NM_183353
A_23_P169003	SH2D4A	278	649	259	524	181	350	199	252	5.69	2.33	4.08	2.02	3.35	1.94	2.20	1.27	0.96	0.65	0.83	P	P	P	P	P	P	P	P	NM_022071
A_33_P3246985	PDE4DIP	262	120	312	214	267	172	366	340	-3.53	0.46	-2.18	0.69	-2.20	0.64	-0.67	0.93	0.94	1.44	1.40	P	P	P	P	P	P	P	P	NM_022359
A_33_P3250953	SLC35B4	248	529	283	524	248	426	257	323	3.42	2.13	3.56	1.85	2.74	1.72	2.11	1.26	0.93	0.73	0.81	P	P	P	P	P	P	P	P	NM_032826
A_33_P3333982	PDE4DIP	241	104	526	323	330	201	371	300	-3.79	0.43	-2.82	0.61	-2.47	0.61	-1.94	0.81	0.99	1.33	1.41	P	P	P	P	P	P	P	P	NM_022359
A_33_P3360675	PRKAR1B	235	115	156	149	129	122	154	152	-3.24	0.49	-0.25	0.96	-0.25	0.95	-0.14	0.98	0.99	1.04	1.94	P	P	P	P	P	P	P	P	NM_001164761
A_23_P170733	ANTXR2	232	104	152	123	350	316	246	228	-3.63	0.45	-1.23	0.81	-0.50	0.90	-0.69	0.93	1.12	1.03	2.02	P	P	P	P	P	P	P	P	NM_058172
A_23_P213085	SLC10A7	193	413	161	245	124	159	106	137	3.45	2.15	2.46	1.53	1.28	1.29	2.37	1.29	0.84	1.01	0.60	P	P	P	P	P	P	P	P	NM_001029998
A_23_P119778	SLC39A10	189	540	174	283	163	181	163	180	4.75	2.86	2.80	1.62	0.53	1.11	0.88	1.10	0.68	0.99	0.39	P	P	P	P	P	P	P	P	NM_020342
A_24_P795594	ALG9	181	373	293	254	327	268	343	327	3.25	2.05	-0.83	0.87	-0.99	0.82	-0.42	0.95	0.95	1.16	0.40	P	P	P	P	P	P	P	P	NM_001077691
A_33_P3393088	RAB6C-AS1	176	357	267	464	216	353	193	206	3.21	2.03	3.21	1.74	2.48	1.63	0.62	1.07	0.94	0.65	0.80	P	P	P	P	P	P	P	P	BC044241
A_23_P315836	BAIAP2	118	345	128	139	144	120	147	131	4.83	2.92	0.46	1.08	-0.91	0.83	-1.05	0.89	0.77	1.07	0.29	P	P	P	P	P	P	P	P	NM_017451
A_23_P118042	LRRC36	115	232	217	454	246	403	176	207	3.19	2.02	4.28	2.09	2.49	1.64	1.52	1.18	0.78	0.72	0.81	P	P	P	P	P	P	P	P	NM_018296
A_21_P0008578	LOC101928725	101	237	165	174	206	184	175	171	3.85	2.34	0.30	1.05	-0.56	0.89	-0.21	0.98	0.85	1.09	0.38	P	P	P	P	P	P	P	P	ENST00000500929

Supplemental Table S6E Specific target genes for 25D3 in siCYP27B1 cells (10).

ProbeID	GeneSymbol	siCT_V1_signal	siCT_1,25D3_signal	siCT_V2_signal	siCT_25D3_signal	siCYP27B1_V_signal	siCYP27B1_25D3_signal	siCYP27B1_siVDR_V_signal	siCYP27B1_siVDR_25D3_signal	siCT_1,25D3/siCT_V1_Zscore	siCT_1,25D3/siCT_V1_ratio	siCT_25D3/siCT_V2_Zscore	siCT_25D3/siCT_V2_ratio	siCYP27B1_25D3/siCYP27B1_V_Zscore	siCYP27B1_25D3/siCYP27B1_ratio	siCYP27B1_siVDR_25D3/siCYP27B1_siVDR_V_Zscore	siCYP27B1_siVDR_25D3/siCYP27B1_siVDR_V_ratio	siCYP27B1_25/V/siCT_25D3/V2_ratio	siCYP27B1_siVDR_25D3/V/siCYP27B1_25D3/V_ratio	siC27B1_25D3/V/siCT_1,25D3/V1_ratio	siCT_V1_flag	siCT_1,25D3_flag	siCT_V2_flag	siCT_25D3_flag	siCYP27B1_V_flag	siCYP27B1_25D3_flag	siCYP27B1_siVDR_V_flag	siCYP27B1_siVDR_25D3_flag	PrimaryAccession
A_33_P3368785	DLGAP1-AS1	913	1562	992	2083	1046	2120	721	1002	3.61	1.71	5.44	2.10	5.16	2.03	4.24	1.39	0.97	0.68	1.19	P	P	P	P	P	P	P	P	NR_024101
A_23_P254888	ZYX	580	1034	673	1072	575	1186	467	552	3.89	1.78	3.42	1.59	5.12	2.06	2.18	1.18	1.29	0.57	1.16	P	P	P	P	P	P	P	P	NM_003461
A_23_P34093	G6PD	567	897	847	2130	740	1958	1006	1338	3.09	1.58	6.77	2.52	6.88	2.65	3.68	1.33	1.05	0.50	1.67	P	P	P	P	P	P	P	P	NM_000402
A_23_P105002	ROM1	517	963	887	2156	679	2924	769	1056	4.19	1.86	6.52	2.43	10.66	4.30	4.09	1.37	1.77	0.32	2.31	P	P	P	P	P	P	P	P	NM_000327
A_23_P217755	SHROOM2	444	724	243	600	256	628	232	331	3.29	1.63	5.25	2.48	6.34	2.45	3.31	1.43	0.99	0.58	1.50	P	P	P	P	P	P	P	P	NM_001649
A_23_P103756	OVGP1	440	226	413	192	445	205	403	309	-3.02	0.51	-4.41	0.47	-3.89	0.46	-2.45	0.77	0.99	1.67	0.90	P	P	P	P	P	P	P	P	NM_002557
A_23_P369899	TMEM158	275	170	334	173	552	250	369	301	-2.16	0.62	-3.80	0.52	-3.58	0.45	-1.89	0.81	0.87	1.80	0.73	P	P	P	P	P	P	P	P	NM_015444
A_23_P131899	SDCBP2	233	126	473	165	2093	960	533	402	-2.79	0.54	-6.11	0.35	-5.70	0.46	-3.63	0.75	1.32	1.65	0.85	P	P	P	P	P	P	P	P	NM_080489
A_21_P0014276		118	195	151	265	148	327	167	206	2.25	1.65	3.27	1.76	4.01	2.21	1.96	1.24	1.26	0.56	1.34	P	P	P	P	P	P	P	P	
A_23_P147805	UPP1	118	229	153	284	121	250	105	111	3.00	1.94	3.60	1.86	3.68	2.07	0.21	1.06	1.11	0.51	1.07	P	P	P	P	P	P	P	P	NM_001287428

Supplemental Table S7 Gene list of Metallothionein genes.

ProbeID	GeneSymbol	siCT_V1_signal	siCT_1,25D3_signal	siCT_V2_signal	siCT_25D3_signal	siCYP27B1_V_signal	siCYP27B1_25D3_signal	siCYP27B1_siVDR_V_signal	siCYP27B1_siVDR_25D3_signal
A_23_P106844	MT2A	27944	7668	29692	14177	30083	25807	33511	24943
A_23_P54840	MT1A	22372	15066	14483	10245	17034	15390	16894	13789
A_33_P3233645	MT1G	10806	2088	6493	2983	9896	7785	8926	6027
A_23_P427703	MT1L	8823	1995	5265	2586	6471	5358	6418	4653
A_23_P37983	MT1B	8565	2176	6045	2895	7709	6365	7465	5245
A_33_P3315314	MT1HL1	7062	2098	5552	2867	7058	5971	6508	4742
A_23_P206724	MT1E	7058	1617	4099	1958	5511	4716	4990	3578
A_33_P3350056	MT1X	5959	2087	3706	2150	4575	4111	4709	3490
A_23_P15174	MT1F	3336	812	1243	532	2966	1733	1724	1077
A_23_P129629	MT3	3024	2847	1320	1334	1390	1570	1239	1296
A_33_P3236868	MT1X	1423	750	1046	779	1241	1373	1379	1231
A_23_P161507	MTL5	1092	1088	1196	1303	888	807	850	931
A_33_P3368313	MT1H	334	49	93	46	120	130	125	79
A_24_P25234	MTL5	146	143	95	72	94	98	101	114
A_33_P3780505	MT1DP	109	71	59	42	50	40	71	56
A_33_P3367293	MT1IP	16	13	7	9	16	9	7	4
A_23_P66241	MT1M	5	5	4	5	5	5	5	5
A_23_P420942	MT1E	5	5	4	5	4	5	28	4
A_21_P0000671	MT1E	4	11	6	4	4	4	8	14
A_23_P152398	MT4	4	4	5	8	4	4	4	8

ProbeID	siCT_1,25D3/siCT_V1_Zscore	siCT_1,25D3/siCT_V1_ratio	siCT_25D3/siCT_V2_Zscore	siCT_25D3/siCT_V2_ratio	siCYP27B1_25D3/siCYP27B1_V_Zscore	siCYP27B1_25D3/siCYP27B1_ratio	siCYP27B1_siVDR_25D3/siCYP27B1_siVDR_V_Zscore	siCYP27B1_siVDR_25D3/siCYP27B1_siVDR_V_ratio	siCYP27B1_25/V/siCT_25D3/V2_ratio	siCYP27B1_siVDR_25D3/V/siCYP27B1_25D3/V_ratio	siC27B1_25D3/V/siCT_1,25D3/V1_ratio
A_23_P106844	−11.66	0.27	−7.40	0.48	−1.81	0.86	−3.83	0.74	1.80	0.87	3.13
A_23_P54840	−3.56	0.67	−3.46	0.71	−0.95	0.90	−1.11	0.82	1.28	0.90	1.34
A_33_P3233645	−11.83	0.19	−5.96	0.46	−2.27	0.79	−2.16	0.68	1.71	0.86	4.07
A_23_P427703	−10.70	0.23	−5.45	0.49	−1.78	0.83	−5.14	0.73	1.69	0.88	3.66
A_23_P37983	−9.86	0.25	−5.64	0.48	−1.81	0.83	−1.94	0.70	1.72	0.85	3.25
A_33_P3315314	−8.73	0.30	−5.07	0.52	−1.58	0.85	−5.06	0.73	1.64	0.86	2.85
A_23_P206724	−10.60	0.23	−5.66	0.48	−1.14	0.86	−5.31	0.72	1.79	0.84	3.73
A_33_P3350056	−7.55	0.35	−4.18	0.58	−0.79	0.90	−4.79	0.74	1.55	0.82	2.57
A_23_P15174	−10.17	0.24	−6.22	0.43	−3.93	0.58	−6.06	0.62	1.37	1.07	2.40
A_23_P129629	−0.43	0.94	0.09	1.01	0.89	1.13	0.59	1.05	1.12	0.93	1.20
A_33_P3236868	−4.32	0.53	−2.15	0.74	0.72	1.11	−1.45	0.89	1.48	0.81	2.10
A_23_P161507	−0.03	1.00	0.63	1.09	−0.66	0.91	1.19	1.10	0.83	1.21	0.91
A_33_P3368313	−8.65	0.15	−2.60	0.49	0.42	1.08	−1.81	0.64	2.21	0.59	7.35
A_24_P25234	−0.11	0.98	−1.01	0.76	0.24	1.05	0.48	1.13	1.38	1.08	1.07
A_33_P3780505	−0.70	0.65	−1.26	0.71	−0.64	0.80	−0.95	0.79	1.13	0.99	1.23
A_33_P3367293	NULL	NULL	NULL	NULL	NULL	NULL	NULL	NULL	#VALUE!	#VALUE!	#VALUE!
A_23_P66241	NULL	NULL	NULL	NULL	NULL	NULL	NULL	NULL	#VALUE!	#VALUE!	#VALUE!
A_23_P420942	NULL	NULL	NULL	NULL	NULL	NULL	−2.14	0.16	#VALUE!	#VALUE!	#VALUE!
A_21_P0000671	NULL	NULL	NULL	NULL	NULL	NULL	NULL	NULL	#VALUE!	#VALUE!	#VALUE!
A_23_P152398	NULL	NULL	NULL	NULL	NULL	NULL	NULL	NULL	#VALUE!	#VALUE!	#VALUE!

ProbeID	siCT_V1_flag	siCT_1,25D3_flag	siCT_V2_flag	siCT_25D3_flag	siCYP27B1_V_flag	siCYP27B1_25D3_flag	siCYP27B1_siVDR_V_flag	siCYP27B1_siVDR_25D3_flag	PrimaryAccession
A_23_P106844	P	P	P	P	P	P	P	P	NM_005953
A_23_P54840	P	P	P	P	P	P	P	P	NM_005946
A_33_P3233645	P	P	P	P	P	P	P	P	NM_005950
A_23_P427703	P	P	P	P	P	P	P	P	NR_001447
A_23_P37983	P	P	P	P	P	P	P	P	NM_005947
A_33_P3315314	P	P	P	P	P	P	P	P	NM_001276687
A_23_P206724	P	P	P	P	P	P	P	P	NM_175617
A_33_P3350056	P	P	P	P	P	P	P	P	NM_005952
A_23_P15174	P	P	P	P	P	P	P	P	NM_005949
A_23_P129629	P	P	P	P	P	P	P	P	NM_005954
A_33_P3236868	P	P	P	P	P	P	P	P	NM_005952
A_23_P161507	P	P	P	P	P	P	P	P	NM_004923
A_33_P3368313	P	P	P	P	P	P	P	P	NM_005951
A_24_P25234	P	P	P	P	P	P	P	P	NM_001039656
A_33_P3780505	P	P	P	P	P	P	P	P	NR_003658
A_33_P3367293	A	A	A	A	A	A	A	A	NR_003669
A_23_P66241	A	A	A	A	A	A	A	A	NM_176870
A_23_P420942	A	A	A	A	A	A	P	A	XM_005255956
A_21_P0000671	A	A	A	A	A	A	A	A	NM_175617
A_23_P152398	A	A	A	A	A	A	A	A	NM_032935

Figure S1 Susa et al.

25D3 did not show the anti-proliferative function to LNCaP cells

For cell proliferation assay, the cells were treated with each steroid hormone in 10 % CS-FBS-medium for another 144 h, then the number of cells was counted. Relative cell numbers are presented with respect to vehicle treatment. *$P < 0.05$ compared with each vehicle. Data are presented as means ± SD (n=4).

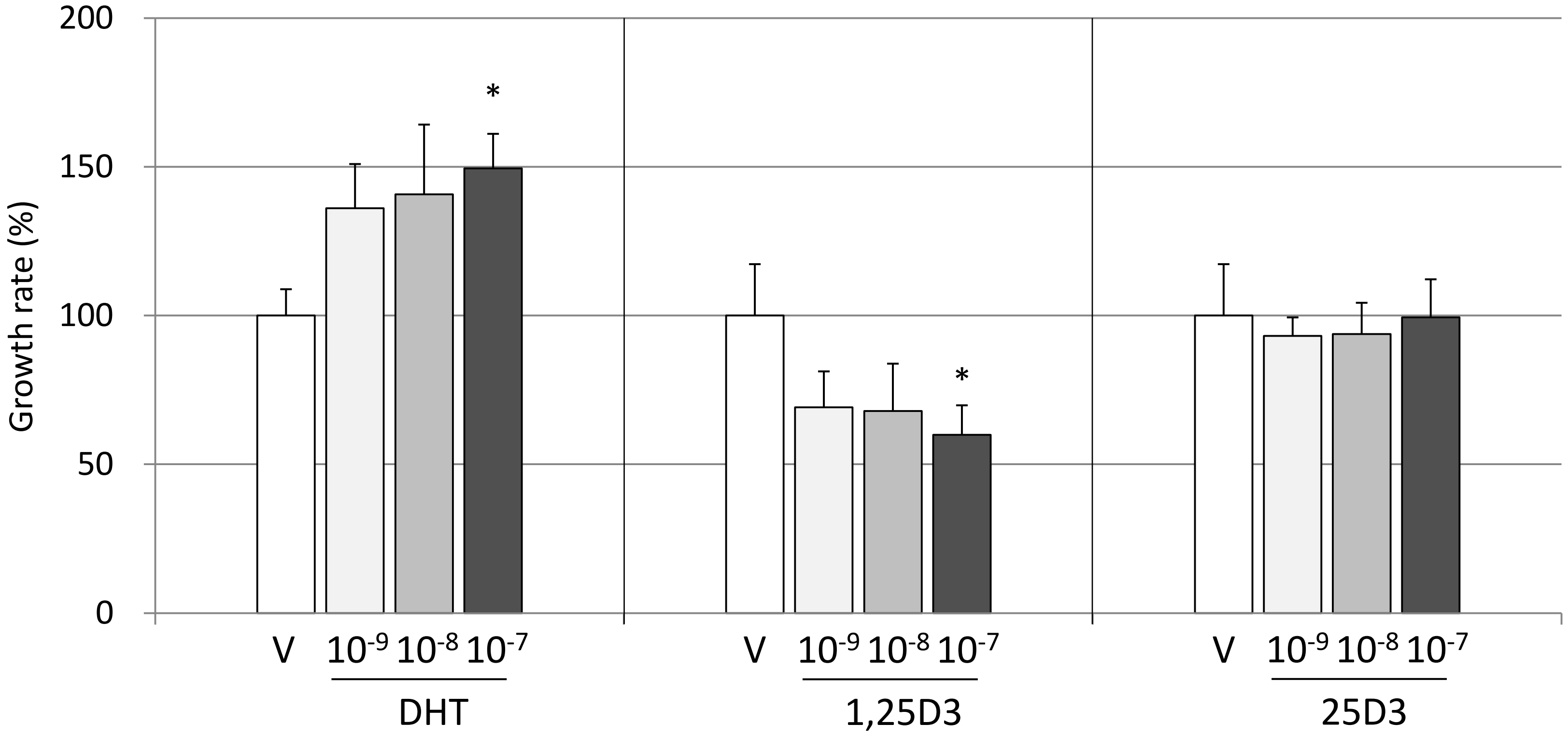

Supplemental Figure S2 Susa et al.

Uncropped blots used in Figure 1D.

Short exposure (a) and long exposure (b) of the same immunoblots.

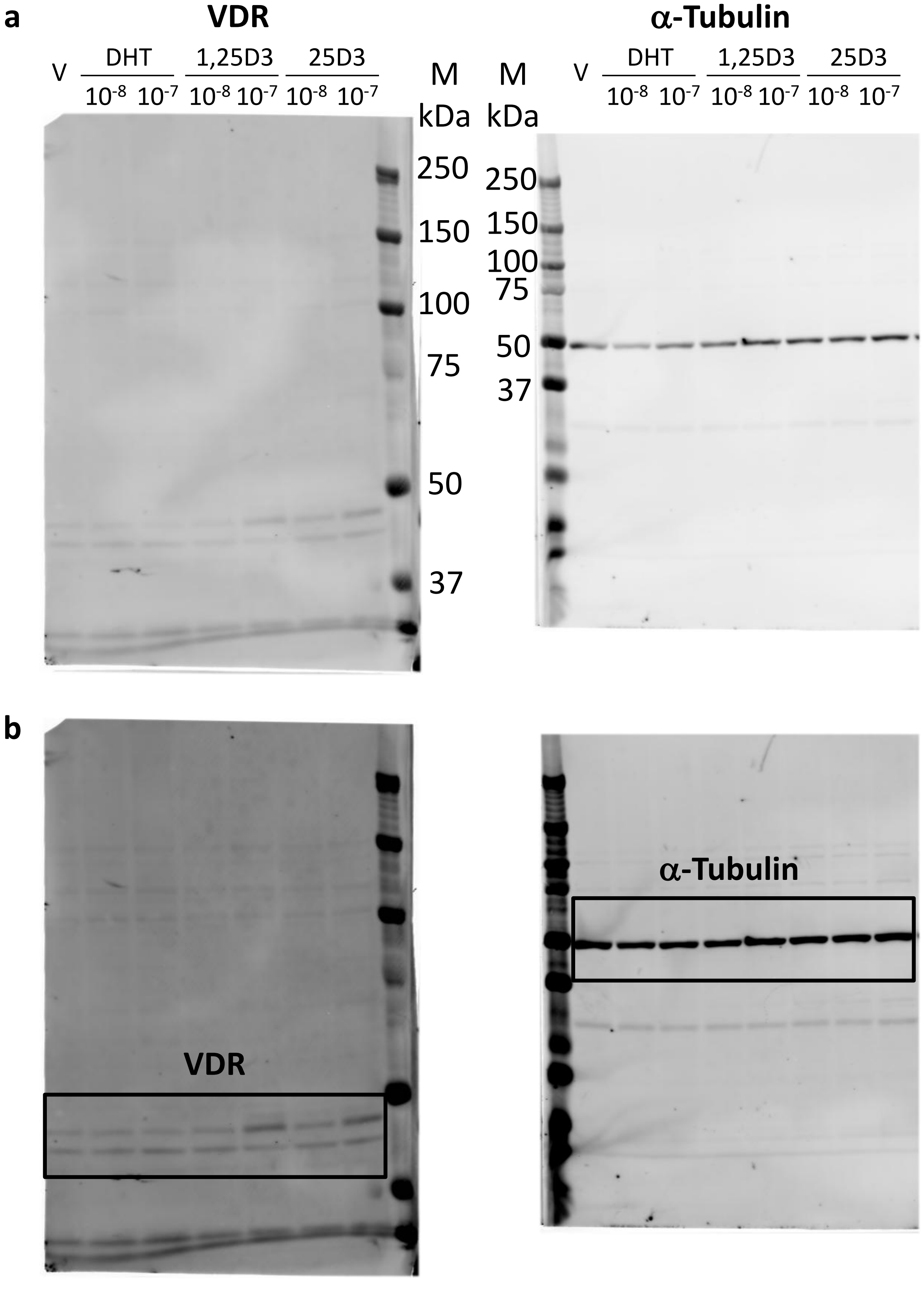

Supplement: Supplementary file 1 — Supplementary Information [file 41598_2018_27441_MOESM1_ESM.pdf]
